# Sustainability of knowledge translation interventions in healthcare decision-making: a scoping review

**DOI:** 10.1186/s13012-016-0421-7

**Published:** 2016-04-21

**Authors:** Andrea C. Tricco, Huda M. Ashoor, Roberta Cardoso, Heather MacDonald, Elise Cogo, Monika Kastner, Laure Perrier, Ann McKibbon, Jeremy M. Grimshaw, Sharon E. Straus

**Affiliations:** 1 Knowledge Translation Program, Li Ka Shing Knowledge Institute, St. Michael’s Hospital, 209 Victoria Street, East Building, Toronto, ON M5B 1T8 Canada; 2 Epidemiology Division, Dalla Lana School of Public Health, University of Toronto, 155 College Street, 6th floor, Toronto, ON M5T 3M7 Canada; 3 Institute of Health Management, Policy and Evaluation, University of Toronto, 155 College Street, Toronto, ON M5T 3M6 Canada; 4 Department of Clinical Epidemiology and Biostatistics, Health Information Research Unit, McMaster University Faculty of Health Sciences, 1200 Main Street West, Hamilton, ON Canada; 5 Ottawa Hospital Research Institute, Center for Practice Changing Research Building, The Ottawa Hospital-General Campus, 501 Smyth Road, PO Box 201B, Ottawa, ON K1H 8L6 Canada; 6 Department of Medicine, University of Ottawa, 451 Smyth Road, Ottawa, ON K1H 8M5 Canada; 7 Department of Geriatric Medicine, University of Toronto, 27 Kings College Circle, Toronto, ON M5S 1A1 Canada

**Keywords:** Knowledge translation, Implementation, Sustainability, Long-term, Maintenance, Adherence, Quality improvement, Patient education, Self-management, Healthcare utilization

## Abstract

**Background:**

Knowledge translation (KT, also known as research utilization, and sometimes referring to implementation science) is a dynamic and iterative process that includes the synthesis, dissemination, exchange, and ethically sound application of knowledge to improve health. A KT intervention is one which facilitates the uptake of research. The long-term sustainability of KT interventions is unclear. We aimed to characterize KT interventions to manage chronic diseases that have been used for healthcare outcomes beyond 1 year or beyond the termination of initial grant funding.

**Methods:**

We conducted a scoping review by searching MEDLINE, Embase, Cochrane Central Register of Controlled Trials (CENTRAL), Cumulative Index to Nursing and Allied Health Literature (CINAHL), and Campbell from inception until February 2013. We included experimental, quasi-experimental, and observational studies providing information on the sustainability of KT interventions for managing chronic diseases in adults and focusing on end-users including patients, clinicians, public health officials, health service managers, and policy-makers. Articles were screened and abstracted by two reviewers, independently. The data were charted and results described narratively.

**Results:**

We included 62 studies reported in 103 publications (total 260,688 patients) plus 41 companion reports after screening 12,328 titles and abstracts and 464 full-text articles. More than half of the studies were randomized controlled trials (RCTs). The duration of the KT intervention ranged from 61 to 522 weeks. Nine chronic conditions were examined across the studies, such as diabetes (34 %), cardiovascular disease (28 %), and hypertension (16 %). Thirteen KT interventions were reported across the studies. Patient education was the most commonly examined (20 %), followed by self-management (17 %). Most studies (61 %) focused on patient-level outcomes (e.g. disease severity), while 31 % included system-level outcomes (e.g. number of eye examinations), and 8 % used both. The interventions were aimed at the patient (58 %), health system (28 %), and healthcare personnel (14 %) levels.

**Conclusions:**

We found few studies focusing on the sustainability of KT interventions. Most of the included studies focused on patient-level outcomes and patient-level KT interventions. A future systematic review can be conducted of the RCTs to examine the impact of sustainable KT interventions on health outcomes.

**Electronic supplementary material:**

The online version of this article (doi:10.1186/s13012-016-0421-7) contains supplementary material, which is available to authorized users.

## Background

Evidence from systematic reviews suggests that numerous knowledge translation (KT) interventions are effective [1, 2]. A KT intervention is one which facilitates the uptake of research into practice and/or policy and can also be referred to as research utilization. When KT interventions are aimed at the clinician, organization, or health system level, these can also be considered implementation science interventions. In order to increase the uptake of KT interventions, researchers within the KT field have focused on surmounting barriers to their initial implementation [3, 4]. However, less research has been done to examine the long-term sustainability of KT interventions [5–8], which can be defined as the extent to which a KT intervention continues after adoption has been secured [9].

The sustainability of KT interventions is paramount to ensure the long-term quality of care for patients [10–13]. It has been suggested that KT interventions that are not sustained in the long-term may result in worse patient outcomes [10, 11, 14], such as decreased quality of care and quality of life. As such, evaluating sustainability is increasingly important in the field of KT [5–8].

Sustainability of interventions is particularly critical in the management of patients with chronic diseases. Half of all US adults (117 million people) have at least 1 chronic condition; 26 % of US adults have ≥2 chronic conditions (including diabetes, hypertension, cancer, arthritis, vascular disease, depression, chronic obstructive pulmonary disease [COPD], and dementia) [15]. More chronic conditions translate to increased risks of functional limitations and admission to acute and long-term care hospitals. In 2006, 84 % of all US healthcare spending was for the 50 % of the population who have ≥2 chronic conditions [16]. This situation is not unique to the US. Chronic diseases are increasing rapidly in prevalence and are recognized by the World Health Organization as the major challenge facing health systems worldwide [17]. Our decision-maker partners [18] have identified sustainability of KT interventions to be a particular challenge in chronic disease management, as most research initiatives and pilot project focus on short-term implementation, yet this does not reflect the needs of the healthcare system [18]. For example, in a recent systematic review of effective KT strategies for coordination of care to reduce use of healthcare services by those who are identified as “frequent users of healthcare” (i.e. those with chronic disease), the majority of the 36 included studies lasted less than 12 months; with just 1 study extending to 3 years [19]. Yet, these patients have chronic disease, implying the intervention should extend beyond 1 year to reflect the course of their disease.

Frameworks for implementing sustainability interventions as well as for measuring sustainability have been proposed [6, 7, 20, 21]. Chambers and colleagues developed a “Dynamic Sustainability Framework” for sustainability involving “continued learning and problem solving, ongoing adaptation of interventions with a primary focus on fit between interventions and multi-level contexts, and expectations for ongoing improvement as opposed to diminishing outcomes over time” [6]. Doyle and colleagues conducted a formative evaluation of the National Health Service Institute for Innovation and Improvement Sustainability Model (SM), which provides information on 10 factors that may improve sustainability for teams who are implementing new practice in their organization [7]. Schell et al. developed a sustainability framework specific to public health interventions that includes nine domains that are essential for success [20]. Simpson et al. describe their model that was developed to sustain oral health interventions including addressing barriers and considering contextual factors [21]. These frameworks are likely useful for empirical research to develop, implement, or measure sustainability of KT interventions. However, this has not been formally evaluated.

We aimed to conduct a scoping review of KT intervention research to characterize KT interventions to manage chronic disease that have been used for healthcare outcomes beyond 1 year or beyond the termination of funding. We also aimed to determine the uptake of frameworks that focus on the sustainability of KT interventions in the included studies.

## Methods

### Protocol

A protocol for our scoping review was developed using the methods of Arksey and O’Malley [22] and others [23] and published in a peer-reviewed journal [24]. A scoping review “maps the concepts underpinning a research area and identifies the main sources and types of evidence available. Scoping reviews can be used to identify gaps in knowledge, establish research agendas, and discuss implications for decision-making” [25]. Since the full methods have been published, they are only described briefly in this paper.

### Eligibility criteria

We included studies that targeted adults with chronic disease (excluding mental illness) who received a KT intervention (which may have targeted the patient, their healthcare provider, or the health system). The list of chronic diseases is presented in Additional file 1. Studies including patients with chronic diseases but without specifying the conditions were included. All comparators were eligible for inclusion, such as other KT interventions or usual care. The study designs included were experimental (randomized controlled trials (RCTs), quasi-RCTs, non-RCTs), quasi-experimental (controlled before-after studies, interrupted time series), and observational studies with a comparator group(s) (i.e. comparative cohort and case control studies).

There is a lack of clarity and agreement on the definition (and the term) for sustainability [8]. Given our focus on chronic disease management, the evidence from the systematic review on KT interventions for those who are identified as “frequent users of the healthcare system,” [19] and in discussion with our knowledge users, it was felt that we should focus on studies that extended beyond 1 year of initial implementation to reflect the critical health system challenge. Moreover, given that a common concern of funders is what happens when research funding for a KT intervention ends [26], it was decided to also include those studies that looked at sustainability after the termination of research funds. As such, studies lasting more than 1 year after implementation or the termination of the study funding across all clinical settings were included.

### Information sources and literature search

Comprehensive literature searches were conducted from inception until February 2013 in the MEDLINE, Embase, Cochrane Central Register of Controlled Trials (CENTRAL), Cumulative Index to Nursing and Allied Health Literature (CINAHL) and the Campbell databases. The MEDLINE search strategy was peer-reviewed by another librarian using the PRESS checklist [27] and is available in our protocol publication [24]. Search terms included durability, fidelity, sustainability, institutionalization, routinization, longitudinal and long-term. The search strategies for the other databases are available from the corresponding author upon request. References from 15 relevant review articles [11, 28–41] were searched to identify any additional studies.

### Study selection process

The team calibrated the eligibility criteria using a random sample of 50 titles and abstracts screened independently by each reviewer. Two calibration exercises (using 50 records each time) were necessary for the team to reach 90 % agreement after clarifications on eligibility criteria were discussed amongst the team. Subsequently, pairs of team members independently screened the titles and abstracts for inclusion. Disagreements were resolved by discussion amongst pairs of reviewers or with a third member, if required. The same process was followed for full-text screening, except that 2 calibration exercises of 15 random full-text articles occurred prior to achieving 90 % agreement.

### Data items and data abstraction process

The abstracted data included terminology used to describe sustainability, study characteristics (e.g. type of study design, year of study conduct, funding source, KT duration), patient characteristics (e.g. number of patients, number of clusters, type of chronic condition), outcomes examined, and interventions (e.g. frequency, duration, provider, target). A post hoc analysis was conducted to determine whether sustainability frameworks were used to inform the included studies. Using a random sample of five included studies, the pre-specified data abstraction form was calibrated amongst the team. Three such exercises were necessary prior to embarking on full data abstraction, which was undertaken by pairs of team members independently. A third team member verified all of the abstracted data by comparing the data abstraction with the original papers, to ensure accuracy. The KT interventions were coded independently by a clinician (SES) and a methodologist (ACT) on our team using a pre-existing taxonomy originally developed by the Cochrane EPOC group, revised by members of the Agency for Healthcare Research and Quality, and used in subsequent publications (Additional file 2) [42]. Conflicts in the KT intervention codes were resolved through discussion. Companion reports were identified by matching the authors, KT intervention, and timeframe for the study conduct. The main report was the one with the longest duration of follow-up; companion reports were used for supplementary material only.

### Methodological quality appraisal

We did not appraise methodological quality or risk of bias of the included articles because this is a scoping review. This approach is consistent with scoping reviews of clinical topics [43].

### Synthesis

The abstracted data from the included studies were charted using frequencies for the following variables: year of publication, study period, geographic region of conduct, study design, source of funding, duration of KT intervention, setting, duration of follow-up, number of patients, age range, percent female, type of chronic disease, number of conditions, type of KT intervention, target of KT intervention, level of KT intervention, fidelity of the KT intervention (defined as “the consistency and quality of targeted organizational members’ use of the specific innovation” [44]), whether the KT intervention was adapted for the setting, types of outcomes examined, and who the target was for the outcomes. Gaps in the literature were identified, as well as areas for future systematic reviews. Word clouds were drawn using the online program Wordle [45] for the terms used to describe sustainability (as described by study authors).

## Results

### Literature search

**Fig. 1:**
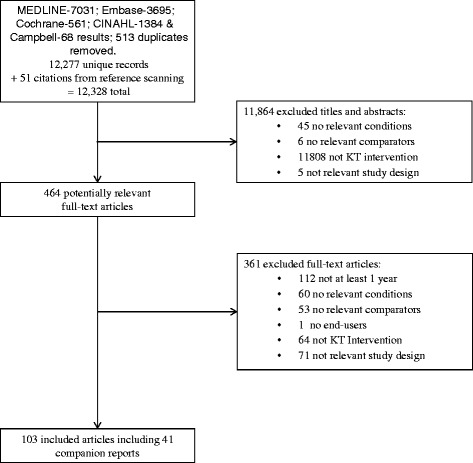
Study flow. Details the flow of information through the different phases of the review; maps out the number of records identified, included and excluded, and the reasons for their exclusion

After screening 12,328 citations and 464 full-text papers, 62 studies (plus 41 companion reports) were included. Details of search results per database and the duplicates removed are presented in Fig. 1. The full list of citations for the included studies can be found in Additional file 3.

### Terminology for sustainability

**Fig. 2:**
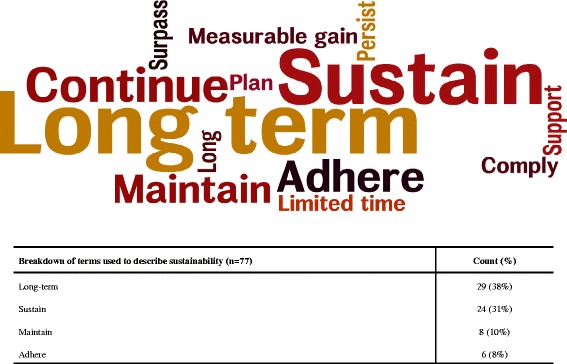
Word cloud displaying sustainability terminology. The most commonly used terminology in the 103 included studies, with the size of the terms in the word cloud corresponding to the frequency of their use

Only 15 % (8/62) of the included studies defined or described sustainability. The first study to provide a definition was published in 1996, where the authors described this as occurring when patient results identified earlier remain when the treatment groups have returned to routine care [46]. Six of the eight studies describing sustainability were published after 2005. Most focused on sustainability of interventions or outcomes or treatment goals. In addition, 79 % (49/62) used a term to describe sustainability. Of the studies providing a term for sustainability, the most commonly used term for sustainability was long-term (29/77, 38 %), which was followed by sustain (24/77, 31 %), maintain (8/77, 10 %), and adhere (6/77, 8 %) (Fig. 2). The use of the terms long-term and sustain were consistent over time. Figure 2 presents a word cloud of the sustainability terminology used in the included studies, with larger words representing more frequent usage. Further details of the nine definitions identified and the other terms used are presented in Additional file 4.

### Study characteristics

The year of publication ranged from 1979 to 2012, while the study conduct period ranged from 1974 to 2010 (Table 1). More than half of the studies were published after 2003, suggesting that KT sustainability is a relatively new concept for KT research. The studies were most commonly conducted in North America (39/62, 63 %) and Europe (16/62, 26 %). More than half of the included studies were RCTs. The funding source was most commonly a governmental organization (23 %) or not reported (24 %). The duration of the KT intervention was 61 to 104 weeks in 61 % of included studies (range 61–522 weeks) and the total duration of follow-up was 61 to 104 weeks in 55 % of studies. Most of the study settings were multi-site (58 %), and most had two study arms (82 %). Further details, including specific study site, study setting, and organizational context, can be found in Additional file 4.

**Table 1:** Summary of study characteristics

Characteristic	Number (% out of 62)
Year of publication	
1979 to 1986	5 (8 %)
1987 to 1994	2 (3 %)
1995 to 2002	15 (24 %)
2003 to 2010	32 (52 %)
2011	4 (6 %)
2012	4 (6 %)
Study period	
1970 to 1980	4 (6 %)
1981 to 1990	3 (5 %)
1991 to 2000	21 (34 %)
2001 to 2010	17 (27 %)
Not reported	17 (27 %)
Geographic region of conduct	
North America	39 (63 %)
Europe	16 (26 %)
Australia	4 (6 %)
Asia	2 (3 %)
South America	1 (2 %)
Study design	
Randomized controlled trial	31 (50 %)
Cohort	24 (39 %)
Cluster-randomized controlled trial	2 (3 %)
Non-randomized controlled trial	2 (3 %)
Case control	1 (2 %)
Controlled before-after study	1 (2 %)
Quasi-randomized controlled trial	1 (2 %)
Funding sources	
Not reported	19 (24 %)
Governmental organization	18 (23 %)
Research funding body	16 (21 %)
Commercial organization	11 (14 %)
Healthcare provider organization	6 (8 %)
Charitable trust	3 (4 %)
Voluntary body	2 (3 %)
Mixed	2 (3 %)
Other	1 (1 %)
Duration of knowledge translation intervention (in weeks)	
61 to 104	38 (61 %)
104 to 157	10 (16 %)
157 to 209	4 (6 %)
209 to 261	3 (5 %)
261 to 313	0 (0 %)
313 to 365	1 (2 %)
365 to 417	1 (2 %)
417 to 470	0 (0 %)
470 to 522	5 (8 %)
Setting	
Multi-site	36 (58 %)
Single-site	25 (40 %)
Not reported	1 (2 %)
Number of study arms	
2	51 (82 %)
3	4 (6 %)
4	6 (10 %)
8	1 (2 %)
Follow-up duration in weeks	
52 to 104	34 (55 %)
104 to 157	11 (18 %)
157 to 209	5 (8 %)
209 to 261	4 (6 %)
261 to 313	0 (0 %)
313 to 365	1 (2 %)
365 to 417	1 (2 %)
417 to 470	0 (0 %)
470 to 522	5 (8 %)
Not reported	1 (2 %)

### Patient characteristics

Across all studies, the total number of included patients was 260,688, with an average of 4495 patients per study. Their age ranged from 18 to 99 years and the average percent female was approximately 48. Most of the studies included patients with a single condition (87 %), with diabetes being the most common (34 %) (Table 2). Further data on the patient characteristics, including end-users, comorbidities, risk factors, history of treatment utilization, concomitant therapies, and eligibility criteria, can be found in Additional file 5.

**Table 2:** Summary of patient characteristics

Total number of patients 260,688, mean per study 4495	
Age range: 18 to 99	
Mean % female: 47.85 (11 NR)	
Patient characteristics	Number (% out of 62)
Chronic disease (multiple reported per study)	
Diabetes	26 (34 %)
Cardiovascular diseases	21 (28 %)
Hypertension	12 (16 %)
Chronic Obstructive Pulmonary Disease	7 (9 %)
Asthma	3 (4 %)
Cancer	3 (4 %)
Arthritis	2 (3 %)
Unspecified chronic illness	1 (1 %)
Renal disease	1 (1 %)
Number of conditions	
Single condition	54 (87 %)
Multiple conditions	8 (13 %)

### KT interventions

A total of 13 interventions were identified (Table 3). The interventions were delivered at the patient (58 %), health system (28 %), and healthcare personnel (14 %) levels, using the previous coding scheme for the different KT interventions. The most commonly examined type of KT intervention was patient education (83/409, 20 %), followed by self-management (70/409, 17 %) (Table 3). In contrast, the least commonly used KT interventions were continuous quality improvement (5/409, 1 %) and facilitated relay of clinical information (5/409, 1 %). Interventions were commonly targeted to patients (236/315, 75 %) and healthcare providers (49/315, 16 %). A detailed description of the interventions examined across the studies can be found in Additional files 6 and 7.

**Table 3:** Summary of interventions

Intervention characteristics	Number (%)
Knowledge translation interventions/control out of 409 total	
Patient education	83 (20 %)
Self-management	70 (17 %)
Control/usual care	58 (14 %)
Case management	38 (9 %)
Team change	38 (9 %)
Clinician education	26 (6 %)
Reminders	23 (6 %)
Social support	16 (4 %)
Motivational interviewing	15 (4 %)
Audit and feedback	13 (3 %)
Financial incentive	11 (3 %)
Electronic patient registry	8 (2 %)
Continuous quality improvement	5 (1 %)
Facilitated relay	5 (1 %)
Level of the intervention out of 339 total	
Patient	197 (58 %)
Health system	94 (28 %)
Healthcare personnel	48 (14 %)
Target of the intervention (multiple reported per study) out of 316 total	
Patient	236 (75 %)
Healthcare provider(s)	49 (16 %)
Family	11 (3 %)
Health centre(s)	10 (3 %)
Community	6 (2 %)
Not reported	2 (1 %)
Other	1 (0.3 %)
Fidelity of the intervention out of 62 studies	
No	59 (95 %)
Yes	3 (5 %)
Adaptation of the intervention out of 62 studies	
No	56 (90 %)
Yes	6 (10 %)

Most of the studies did not mention adaptation (i.e. whether the intervention was adapted or changed over time) (56/62, 90 %) or fidelity (59/62, 95 %) of the intervention.

### Outcome characteristics

The most commonly used outcome was healthcare utilization (142/628 outcomes reported across the studies, 23 %) (Table 4, Additional file 8). Most studies (61 %) focused on patient-level outcomes (e.g. disease severity), while 31 % included system-level outcomes (e.g. number of eye examinations) and 8 % used both. None of the studies reported the use of outcomes to assess for sustainability.

**Table 4:** Summary of outcomes

Outcome characteristics	Number (%)
Type of outcome out of 628 total	
Healthcare utilization	142 (23 %)
Blood pressure	65 (10 %)
Glycemic control	47 (7 %)
Function	38 (6 %)
Overall mortality and cause-specific mortality	38 (6 %)
Cholesterol	34 (5 %)
Compliance	34 (5 %)
Cardiovascular health	34 (5 %)
Body mass index	31 (5 %)
Pulmonary function	16 (3 %)
Renal function	15 (2 %)
Cost	13 (2 %)
Diet	11 (2 %)
Satisfaction	11 (2 %)
Mental health	10 (2 %)
Quality of life	8 (1 %)
Attitude	7 (1 %)
Behaviour	6 (1 %)
Health status	6 (1 %)
Knowledge	6 (1 %)
Safety	6 (1 %)
Smoking	5 (1 %)
Vision health	5 (1 %)
Cognition	4 (1 %)
Disease severity	4 (1 %)
Employment	4 (1 %)
Motivation	4 (1 %)
Vision	4 (1 %)
Alcohol intake	3 (0 %)
Barrier/facilitator	3 (0 %)
Nerve function	3 (0 %)
Self-efficacy	3 (0 %)
Level of care	2 (0 %)
Comorbidity	1 (0 %)
Discontinuation	1 (0.2 %)
Feasibility	1 (0.2 %)
Fidelity	1 (0.2 %)
Liver function	1 (0.2 %)
Pain	1 (0.2 %)
Level of outcome out of 616 total	
Patient level	377 (61 %)
System level	191 (31 %)
Both	48 (8 %)

### Use of frameworks on sustainability

Our post hoc analysis indicated that none of the included studies reported using a framework to develop, implement, or measure sustainability.

## Discussion

It has been postulated that while nearly $300 billion is spent on research globally, much of this is wasted because of poor implementation [47–50]. Sources of waste include lack of consideration of sustainability of effective interventions. This waste is a particular challenge when considering how to optimize care of patients with chronic diseases given the growing proportion of these patients and their impact on health systems. Our scoping review found limited studies on sustainability of KT interventions for people with chronic diseases. Similar to what was postulated in a consensus project on gaps in sustainability research [8], we found that there is a need for clarity on the terms and definitions used to describe sustainability, which would enhance our ability to find this literature.

In addition, we found few studies that tested KT interventions beyond 2 years. This could be due to various reasons, such as a lack of funding or the belief that KT sustainability is not the top priority. As well, we were unable to identify any studies that used a framework to develop, implement, or measure sustainability of KT interventions. This would allow individuals to test different models of sustainability to determine which ones are the most optimal. Our results suggest that KT sustainability is in its infancy in the literature.

To ensure longevity, it has been suggested that planning for sustainability should be done early, when KT interventions are being designed [51]. In particular, theories, process models, and frameworks should be considered when trying to develop, implement, or evaluate KT interventions and their sustainability. However, our review found no studies that reported use of a framework to consider sustainability of a KT intervention. Testing sustainability frameworks empirically is also an area of future research. Moreover, the studies focused on KT interventions focused on single chronic diseases rather than patients with multiple conditions, failing to reflect the complexities of real-world clinical practice and policy.

It is plausible to postulate that depending on the nature and target audience of the intervention, the type of sustainability effort may differ. For example, a simple KT intervention in a clinical setting targeting patients (such as patient reminders) might not require extensive sustainability endeavours. However, more complex KT interventions at the organization or health system level (i.e. implementation science), such as financial incentives, may require more extensive sustainability initiatives. This is an area for future empirical research.

Most of the included studies focused on KT interventions at the patient level, such as patient education and self-management. This finding might be explained by accessibility of the KT intervention; for example, patient-oriented interventions are often easier to employ than more resource-intensive interventions, such as team changes or case management. Across all of the chronic conditions examined by the included studies, the most common was diabetes.

Although we did not formally appraise the methodological quality of included studies, we identified some limitations worth noting. Most of the studies did not mention fidelity or adaptation of the intervention, which should be mentioned in future KT sustainability studies to increase transparency and quality of reporting; indeed, these elements have been suggested in the checklist proposed to enhance reporting of interventions (TIDieR) [52]. As well, the quality of reporting of these studies was low overall and could be improved. For example, the duration of the KT intervention period was difficult to discern across the included studies. In addition, our results found a significant gap in “sustainability” terminology, with only nine (15 %) included studies providing a definition, and the individual terms used were not consistent across studies.

There are some limitations to our scoping review process that are worth mentioning. Due to the large number of citations identified (>12,000), we were unable to search unpublished literature or include studies on mental illness because of resource restraints. Although this is a deviation from our protocol [24], only half of the published scoping reviews in the literature do an extensive search for grey literature [43]. As well, we had hoped to develop a framework for developing, implementing or evaluating sustainability of KT interventions for chronic disease management but were unable to do so due to the dearth of included studies. Since sustainability was poorly reported across studies, we were also unable to formally evaluate factors that influence sustainability of KT interventions. Our scoping review was resource- and time-intensive due to the large screening yield, as well as the unanticipated time required to independently categorize the 13 identified KT interventions, which appeared 464 times across the included papers. Although our literature search is outdated, the purpose of our scoping review was to chart the literature on sustainability initiatives and identify areas to inform the conduct of a future systematic review. We are currently in the process of updating the literature search from our scoping review, focusing on RCTs. We have identified 31 randomized trials through our scoping review and plan to statistically evaluate the impact of sustainable KT interventions on health outcomes through meta-analysis in our future systematic review.

## Conclusions

We found few studies that focused on sustainability of KT interventions. Most of the included studies focused on patient-level outcomes and patient-level KT interventions. A future systematic review can be conducted of the RCTs to examine the impact of sustainable KT interventions on health outcomes. Our results showed several gaps in the literature worth exploring in future research. In particular, our findings suggest that more work is needed on exploring sustainability of KT interventions for patients with chronic diseases.

## Additional files

Additional file 1: KT Sustainability Chronic Conditions of Interest. (PDF 77 kb)

Additional file 2: Taxonomy of Quality Improvement (QI) Strategies. (PDF 133 kb)

Additional file 3: Included Studies. (PDF 160 kb)

Additional file 4: Study Characteristics. (PDF 136 kb)

Additional file 5: Patient Characteristics. (PDF 200 kb)

Additional file 6: KT Interventions. (PDF 181 kb)

Additional file 7: Specific intervention details from included studies. (PDF 776 kb)

Additional file 8: Outcomes. (PDF 115 kb)

## Abbreviations

KT: knowledge translation
RCTs: randomized controlled trials
