# Supplementary material for: Sustainability of knowledge translation interventions in healthcare decision-making: a scoping review
Source: Implement Sci. 2016 Apr 21;11:55. doi: 10.1186/s13012-016-0421-7 (PMC4839064; doi:10.1186/s13012-016-0421-7)
Supplement: Supplementary file 1 — KT Sustainability Chronic Conditions of Interest. (PDF 77 kb) [file 13012_2016_421_MOESM1_ESM.pdf]

Appendix 1. KT Sustainability Chronic Conditions of Interest

Aneurysm
Angina
Arrhythmia
Arthritis
Asthma
Atherosclerosis
Atrial/ventricular fibrillation
Cancer
Cardiomegaly
Cardiomyopathy
Cardiovascular diseases
Cerebrovascular disorders
Chronic obstructive pulmonary disease
Crohn's disease
Diabetes
Embolism
Heart failure
Hypertension
Inflammatory bowel disease
Irritable bowel disease
Ischemia
Myocardial infarction
Osteoporosis
Peripheral vascular diseases
Stroke
Thrombophlebitis
Thrombosis
Ulcerative colitis
