# Supplementary material for: Sustainability of knowledge translation interventions in healthcare decision-making: a scoping review
Source: Implement Sci. 2016 Apr 21;11:55. doi: 10.1186/s13012-016-0421-7 (PMC4839064; doi:10.1186/s13012-016-0421-7)
Supplement: Supplementary file 2 — Taxonomy of Quality Improvement (QI) Strategies. (PDF 133 kb) [file 13012_2016_421_MOESM2_ESM.pdf]

Appendix 2. Taxonomy of Quality Improvement (QI) Strategies

Target	QI	Definition
Health systems	Case management	Any system for coordinating diagnosis, treatment, or routine management of patients (e.g., arrangement for referrals, follow-up of test results) by a person or multidisciplinary team in collaboration with, or supplementary to, the primary-care clinician. If the study called the intervention "case management" we classified it as such.
	Team changes	Changes to the structure or organisation of the primary health-care team (adding team member, multidisciplinary teams, expansion or revision of professional roles)
	Electronic patient registry	General electronic medical record system or electronic tracking. Do not include websites unless patients were tracked over time. To qualify, it had to be a part of the clinical trial as an intervention (i.e., not pre-existing infrastructure unless used more actively)
	Facilitated relay of information to clinicians	Clinical information collected from patients and transmitted to clinicians by means other than the existing medical record (excluding conventional means of correspondence between clinicians.)
	Continuous QI	Interventions explicitly identified as involving the techniques of continuous QI, total quality management, or plan-do-study-act, or any iterative process for assessing quality problems, developing solutions to those problems, testing their effects, and then reassessing the need for further action
Health-care providers	Audit and feedback	Summary of clinical performance of health care delivered by an individual clinician or clinic over a specified period, which was then transmitted back to the clinician. This strategy was strictly based on clinical data and excluded clinical skills. It could include the number of patients with missing tests and dropouts.
	Clinician education	Interventions designed to promote increased understanding of principles guiding clinical care or awareness of specific recommendations for a target disorder or population of patients. Includes conferences or workshops, distribution of educational materials (written, video, or other), and educational outreach visits.
	Reminders	Paper-based or electronic systems intended to prompt a health professional to recall patient-specific information (eg, most recent HbA1c value) or to do a specific task (eg, foot examination).
	Financial incentives	Interventions with positive or negative financial incentives directed at providers (e.g., linked to adherence to some process of care or achievement of some target outcome).

Patients	Education of patients	Interventions designed to promote greater understanding of a target disorder or to teach specific prevention or treatment strategies, or specific in-person education (e.g., individual or group sessions with diabetes nurse educator; distribution of printed or electronic educational materials).
	Promotion of self-management	Provision of equipment or access to resources to promote self-management. If the study called the intervention promotion of self-management, personalised goal-setting, or action-planning, we included it here. We generally thought this a more active strategy than education of patients
	Reminders	Any effort (e.g., postcards or telephone calls) to remind patients about upcoming appointments or important aspects of self-care.<br>If the intervention included case management, reminders to patients needed to be explicit.
	Financial incentives	Positive or negative financial incentives directed at patients
	Motivational interviewing	Motivational interviewing ("a directive and client-centered counselling style that relies upon identifying and mobilizing the client's intrinsic values and goals to stimulate behaviour change (Miller & Rollnick, 2002), thus encouraging client and family involvement in all aspects of care.")
	Social support	social group activities, community activities, support from peers or caregivers
NA	Usual care/Control	Usual/routine care, no treatment, historical cohort, and placebo
**Abbreviations:** NA – Not available		
