# Supplementary material for: Sustainability of knowledge translation interventions in healthcare decision-making: a scoping review
Source: Implement Sci. 2016 Apr 21;11:55. doi: 10.1186/s13012-016-0421-7 (PMC4839064; doi:10.1186/s13012-016-0421-7)
Supplement: Supplementary file 3 — Included Studies. (PDF 160 kb) [file 13012_2016_421_MOESM3_ESM.pdf]

Appendix 3. Included Studies

	Year	Reference
1.	2012	Belardinelli R, Georgiou D, Cianci G, Purcaro A. 10-year exercise training in chronic heart failure: a randomized controlled trial. *Journal of the American college of cardiology.* 2012; 60(16):1521-1528.
2.	2012	Cheng S-H, Lee T-T, Chen C-C. A longitudinal examination of a pay-for-performance program for diabetes care: evidence from a natural experiment. *Medical care.* 2012; 50(2):109-116.
3.	2012 (CR: Martin, 2011)	Rothschild SK, Martin MA, Swider SM, Lynas CT, Avery EF, Janssen I *et al*. The Mexican-American Trial of Community Health workers (MATCH): design and baseline characteristics of a randomized controlled trial testing a culturally tailored community diabetes self-management intervention. *Contemporary clinical trials.* 2012; 33(2):369-377.
4.	2012	Tamone C, Fonte G, Panico A, Molinatti PA, D'Amelio P, Isaia GC. Impact of a phone follow-up program on persistence with teriparatide or PTH (1–84) treatment. *Calcified tissue international.* 2012; 90(4):272-278.
5.	2012	Toobert DJ, Glasgow RE, Strycker LA, Barrera Jr M, King DK. Adapting and RE-AIMing a heart disease prevention program for older women with diabetes. Translational behavioral medicine. 2012;2(2):180-7.
6.	2011	Baker LC, Johnson SJ, Macaulay D, Birnbaum H. Integrated telehealth and care management program for Medicare beneficiaries with chronic disease linked to savings. *Health Affairs.* 2011; 30(9):1689-1697.
7.	2011 (CR: Toobert, 2012)	Barrera M, Toobert DJ, Strycker LA, Osuna D, King DK, Glasgow RE. Multiple-behavior–change interventions for women with Type 2 diabetes. *Diabetes Spectrum.* 2011; 24(2):75-80.
8.	2011	Gibson TB, Mahoney J, Ranghell K, Cherney BJ, McElwee N. Value-based insurance plus disease management increased medication use and produced savings. *Health Affairs.* 2011; 30(1):100-108.
9.	2011 (CR: Kim, 2008)	Kim MT, Han HR, Hedlin H, Kim J, Song HJ, Kim KB *et al*. Teletransmitted monitoring of blood pressure and bilingual nurse counseling–sustained improvements in blood pressure control during 12 months in hypertensive Korean Americans. *The Journal of Clinical Hypertension.* 2011; 13(8):605-612.
10.	2011	Martin MA, Swider SM, Olinger T, Avery E, Lynas CM, Carlson K et al. Recruitment of Mexican American adults for an intensive diabetes intervention trial. Ethnicity & disease. 2011;21(1):7-12.
11.	2010 (CR: Gilmore, 2007; Chung, 2003)	Chen JY, Kang N, Juarez DT, Hodges KA, Chung RS, Legorreta AP. Impact of a pay-for-performance program on low performing physicians. *J Healthc Qual.* 2010; 32(1):13-21; quiz 21-12.
12.	2010	Desouza CV, Rentschler L, Haynatzki G. The effect of group clinics in the control of diabetes. *Primary care diabetes.* 2010; 4(4):251-254.
13.	2010	Hughes SL, Seymour RB, Campbell RT, Desai P, Huber G, Chang HJ. Fit and Strong!: bolstering maintenance of physical activity among older adults with lower-extremity osteoarthritis. *American journal of health behavior.* 2010; 34(6):750.
14.	2010	Huizinga M, Gebretsadik T, Ulen CG, Shintani A, Michon S, Shackleford L *et al*. Preventing glycaemic relapse in recently controlled type 2 diabetes patients: a randomised controlled trial. *Diabetologia.* 2010; 53(5):832-839.
15.	2010	Wisse W, Rookhuizen MB, de Kruif MD, van Rossum J, Jordans I, ten Cate H *et al*. Prescription of physical activity is not sufficient to change sedentary behavior and improve glycemic control in type 2 diabetes patients. *Diabetes research and clinical practice.* 2010; 88(2):e10-e13.
16.	2010	Xian Y, Pan W, Peterson ED, Heidenreich PA, Cannon CP, Hernandez AF *et al*. Are quality improvements associated with the Get With the Guidelines-Coronary Artery Disease (GWTG-CAD) program sustained over time?: A longitudinal comparison of GWTG-CAD hospitals versus non–GWTG-CAD hospitals. *American heart journal.* 2010; 159(2):207-214.
17.	2009	Chavannes NH, Grijsen M, van den Akker M, Schepers H, Nijdam M, Tiep B et al. Integrated disease

		management improves one-year quality of life in primary care COPD patients: a controlled clinical trial. Primary care respiratory journal : journal of the General Practice Airways Group. 2009;18(3):171-6.
18.	2009	Jia H, Chuang H-C, Wu SS, Wang X, Chumbler NR. Long-term effect of home telehealth services on preventable hospitalization use. *J Rehabil Res Dev.* 2009; 46(5):557-566.
19.	2009	Olson KL, Delate T, Rasmussen J, Humphries TL, Merenich JA. Outcomes of patients discharged from pharmacy-managed cardiovascular disease management. *The American journal of managed care.* 2009; 15(8):497-503.
20.	2009	Radziewicz RM, Rose JH, Bowman KF, Berila RA, O'Toole EE, Given B. Establishing treatment fidelity in a coping and communication support telephone intervention for aging patients with advanced cancer and their family caregivers. *Cancer nursing.* 2009; 32(3):193-202.
21.	2009	Svetkey LP, Pollak KI, Yancy WS, Dolor RJ, Batch BC, Samsa G *et al*. Hypertension Improvement Project Randomized Trial of quality improvement for physicians and lifestyle modification for patients. *Hypertension.* 2009; 54(6):1226-1233.
22.	2009	van Wetering CR, Hoogendoorn M, Mol S, Rutten-van Mölken M, Schols A. Short-and long-term efficacy of a community-based COPD management programme in less advanced COPD: a randomised controlled trial. *Thorax.* 2010; 65(1):7-13.
23.	2008	Bocchi EA, Cruz F, Guimarães G, Moreira LFP, Issa VS, Ferreira SMA *et al*. Long-Term Prospective, Randomized, Controlled Study Using Repetitive Education at Six-Month Intervals and Monitoring for Adherence in Heart Failure Outpatients The REMADHE Trial. *Circulation: Heart Failure.* 2008; 1(2):115-124.
24.	2008	Giannuzzi P, Temporelli PL, Marchioli R, Maggioni AP, Balestroni G, Ceci V *et al*. Global secondary prevention strategies to limit event recurrence after myocardial infarction: results of the GOSPEL study, a multicenter, randomized controlled trial from the Italian Cardiac Rehabilitation Network. *Archives of Internal Medicine.* 2008; 168(20):2194-2204.
25.	2008	Kim MT, Kim EY, Han HR, Jeong S, Lee JE, Park HJ et al. Mail education is as effective as in-class education in hypertensive Korean patients. Journal of clinical hypertension (Greenwich, Conn). 2008;10(3):176-84.
26.	2008	Lawrence DB, Allison W, Chen JC, Demand M. Improving medication adherence with a targeted, technology-driven disease management intervention. *Disease Management.* 2008; 11(3):141-144.
27.	2008	Mildestvedt T, Meland E, Eide GE. How important are individual counselling, expectancy beliefs and autonomy for the maintenance of exercise after cardiac rehabilitation? *Scandinavian journal of public health.* 2008; 36(8):832-840.
28.	2007	Berg GD, Wadhwa S. Health services outcomes for a diabetes disease management program for the elderly. *Disease Management.* 2007; 10(4):226-234.
29.	2007	Chin MH, Drum ML, Guillen M, Rimington A, Levie JR, Kirchhoff AC *et al*. Improving and sustaining diabetes care in community health centers with the health disparities collaboratives. *Medical care.* 2007; 45(12):1135-1143.
30.	2007	Del Sindaco D, Pulignano G, Minardi G, Apostoli A, Guerrieri L, Rotoloni M *et al*. Two-year outcome of a prospective, controlled study of a disease management programme for elderly patients with heart failure. *Journal of Cardiovascular Medicine.* 2007; 8(5):324-329.
31.	2007	Dennison CR, Post WS, Kim MT, Bone LR, Cohen D, Blumenthal RS *et al*. Underserved Urban African American Men: Hypertension Trial Outcomes and Mortality During 5 Years*. *American journal of hypertension.* 2007; 20(2):164-171.
32.	2007	Gilmore AS, Zhao Y, Kang N, Ryskina KL, Legorreta AP, Taira DA et al. Patient outcomes and evidence-based medicine in a preferred provider organization setting: a six-year evaluation of a physician pay-for-performance program. Health services research. 2007;42(6 Pt 1):2140-59; discussion 294-323. doi:10.1111/j.1475-6773.2007.00725.x.
33.	2007	Hess PL, Reingold JS, Jones J, Fellman MA, Knowles P, Ravenell JE *et al*. Barbershops as hypertension detection, referral, and follow-up centers for black men. *Hypertension.* 2007; 49(5):1040-1046.
34.	2007	Jiraporn Getpreechaswas M, Boontorterm N. A model of health services for hypertension in primary care unit in Patumthani Province. *J Med Assoc Thai.* 2007; 90(1):129-136.
35.	2006	Bailie RS, Robinson G, Kondalsamy-Chennakesavan SN, Halpin S, Wang Z. Investigating the sustainability of outcomes in a chronic disease treatment programme. *Social Science & Medicine.*

		2006; 63(6):1661-1670.
36.	2006	Inglis SC, Pearson S, Treen S, Gallasch T, Horowitz JD, Stewart S. Extending the horizon in chronic heart failure effects of multidisciplinary, home-based intervention relative to usual care. *Circulation.* 2006; 114(23):2466-2473.
37.	2005	Maroto Montero JM, Artigao Ramirez R, Morales Duran MD, de Pablo Zarzosa C, Abraira V. [Cardiac rehabilitation in patients with myocardial infarction: a 10-year follow-up study]. *Rev Esp Cardiol.* 2005; 58(10):1181-1187.
38.	2005	Phillips LS, Ziemer DC, Doyle JP, Barnes CS, Kolm P, Branch WT *et al.* An Endocrinologist-Supported Intervention Aimed at Providers Improves Diabetes Management in a Primary Care Site Improving Primary Care of African Americans with Diabetes (IPCAAD) 7. *Diabetes Care.* 2005; 28(10):2352-2360.
39.	2004	Bakitas M, Stevens M, Ahles T, Kirn M, Skalla K, Kane N *et al*. Project ENABLE: A palliative care demonstration project for advanced cancer patients in three settings. *Journal of palliative medicine.* 2004; 7(2):363-372.
40.	2004	Fihn SD, McDonell MB, Diehr P, Anderson SM, Bradley KA, Au DH *et al*. Effects of sustained audit/feedback on self-reported health status of primary care patients. *The American journal of medicine.* 2004; 116(4):241-248.
41.	2004	Group CM-CTDS. Closing the Gap: Effect of Diabetes Case Management on Glycemic Control Among Low-Income Ethnic Minority Populations The California Medi-Cal Type 2 Diabetes Study. *Diabetes Care.* 2004; 27(1):95-103.
42.	2003	Behnke M, Jorres R, Kirsten D, Magnussen H. Clinical benefits of a combined hospital and home-based exercise programme over 18 months in patients with severe COPD. *Monaldi archives for chest disease.* 2003; 59(1):44-51.
43.	2003	Chung RS, Chernicoff HO, Nakao KA, Nickel RC, Legorreta AP. A quality-driven physician compensation model: four-year follow-up study. Journal for healthcare quality : official publication of the National Association for Healthcare Quality. 2003;25(6):31-7.
44.	2003	Gæde P, Vedel P, Larsen N, Jensen GV, Parving H-H, Pedersen O. Multifactorial intervention and cardiovascular disease in patients with type 2 diabetes. *New England Journal of Medicine.* 2003; 348(5):383-393.
45.	2003	Gary TL, Bone LR, Hill MN, Levine DM, McGuire M, Saudek C *et al*. Randomized controlled trial of the effects of nurse case manager and community health worker interventions on risk factors for diabetes-related complications in urban African Americans. *Preventive medicine.* 2003; 37(1):23-32.
46.	2002	Allen-Rameym FC, Diette GB, McDonald RC, Skinner EA, Steinwachs DM, Wu AW. Methods Aimed at Improving Asthma Care and Outcomes Management. *Disease Management & Health Outcomes.* 2002; 10(8):495-503.
47.	2002	Froehlich JB, Karavite D, Russman PL, Erdem N, Wise C, Zelenock G *et al*. American College of Cardiology/American Heart Association preoperative assessment guidelines reduce resource utilization before aortic surgery. *Journal of vascular surgery.* 2002; 36(4):758-e751.
48.	2001	Baker AM, Lafata JE, Ward RE, Whitehouse F, Divine G. A Web-based diabetes care management support system. *Joint Commission Journal on Quality and Patient Safety.* 2001; 27(4):179-190.
49.	2000	Coleman E, Eilertsen T, Kramer A, Magid D, Beck A, Conner D. Reducing emergency visits in older adults with chronic illness. A randomized, controlled trial of group visits. *Effective clinical practice: ECP.* 2000; 4(2):49-57.
50.	2000	Hedges JR, Feldman HA, Bittner V, Goldberg RJ, Zapka J, Osganian SK *et al*. Impact of Community Intervention to Reduce Patient Delay Time on Use of Reperfusion Therapy for Acute Myocardial Infarction Rapid Early Action for Coronary Treatment (REACT) Trial. *Academic Emergency Medicine.* 2000; 7(8):862-872.
51.	2000	Rowley KG, Daniel M, Skinner K, Skinner M, White GA, O'Dea K. Effectiveness of a community-directed healthy lifestyle'program in a remote Australian Aboriginal community. *Australian and New Zealand journal of public health.* 2000; 24(2):136-144.
52.	2000	Skinner CS, Arfken CL, Waterman B. Outcomes of the Learn, Share & Live breast cancer education program for older urban women. *American Journal of Public Health.* 2000; 90(8):1229.
53.	2000	Stroebel RJ, Broers JK, Houle SK, Scott CG, Naessens JM. Improving hypertension control: a team approach in a primary care setting. *Joint Commission Journal on Quality and Patient Safety.* 2000; 26(11):623-632.

54.	1999	Daniel M, Green LW, Marion SA, Gamble D, Herbert CP, Hertzman C *et al*. Effectiveness of community-directed diabetes prevention and control in a rural Aboriginal population in British Columbia, Canada. *Social science & medicine.* 1999; 48(6):815-832.
55.	1999	Grosbois J-M, Lamblin C, Lemaire B, Chekroud H, Dernis J-M, Douay B *et al*. Long-term benefits of exercise maintenance after outpatient rehabilitation program in patients with chronic obstructive pulmonary disease. *Journal of Cardiopulmonary Rehabilitation and Prevention.* 1999; 19(4):216-225.
56.	1999	Higginbotham N, Heading G, McElduff P, Dobson A, Heller R. Reducing coronary heart disease in the Australian Coalfields: evaluation of a 10-year community intervention. *Social science & medicine.* 1999; 48(5):683-692.
57.	1998	Pill R, Stott N, Rollnick S, Rees M. A randomized controlled trial of an intervention designed to improve the care given in general practice to type II diabetic patients: patient outcomes and professional ability to change behaviour. *Family Practice.* 1998; 15(3):229-235.
58.	1997	Corkery E, Palmer C, Foley ME, Schechter CB, Frisher L, Roman SH. Effect of a bicultural community health worker on completion of diabetes education in a Hispanic population. *Diabetes Care.* 1997; 20(3):254-257.
59.	1996	Kelso TM, Abou-Shala N, Heilker GM, Arheart KL, Portner TS, Self TH. Comprehensive long-term management program for asthma: effect on outcomes in adult African-Americans. *The American journal of the medical sciences.* 1996; 311(6):272-280.
60.	1996	Reichard P, Pihl M, Rosenqvist U, Sule J. Complications in IDDM are caused by elevated blood glucose level: the Stockholm Diabetes Intervention Study (SDIS) at 10-year follow up. *Diabetologia.* 1996; 39(12):1483-1488.
61.	1991	Erfurt JC, Foote A, Heirich MA. Worksite wellness programs: incremental comparison of screening and referral alone, health education, follow-up counseling, and plant organization. *American Journal of Health Promotion.* 1991; 5(6):438-448.
62.	1988	Perk J, Hedbäck B, Jutterdal S. Cardiac rehabilitation: evaluation of a long-term programme of physical training for out-patients. *Scandinavian journal of rehabilitation medicine.* 1988; 21(1):13-17.
63.	1984	Hopper SV, Miller JP, Birge C, Swift J. A randomized study of the impact of home health aides on diabetic control and utilization patterns. *American journal of public health.* 1984; 74(6):600-602.
64.	1984	Weiss LJ, Sklar BW. Project OPEN: A hospital-based long-term care demonstration program for the chronically ill elderly. *Home health care services quarterly.* 1984; 4(3-4):127-145.
65.	1983	Morisky DE, Levine DM, Green LW, Shapiro S, Russell RP, Smith CR. Five-year blood pressure control and mortality following health education for hypertensive patients. *American Journal of Public Health.* 1983; 73(2):153-162.
66.	1982	Ambrosio G, Zamboni S, Dal Palu C. Treatment of hypertension: the community approach. *Postgraduate medical journal.* 1982; 59:131-133.
67.	1979	Krishan I, Brennan Jr L, Nobrega F, Smoldt R, Smutka L, Labarthe D *et al*: The Mayo Three-Community Hypertension Control Program. II. Outcome of intervention in entire communities. In: *Mayo Clinic Proceedings: 1979*; 1979: 299-306.
**Abbreviations: CR** – Companion Report		
