# Supplementary material for: Sustainability of knowledge translation interventions in healthcare decision-making: a scoping review
Source: Implement Sci. 2016 Apr 21;11:55. doi: 10.1186/s13012-016-0421-7 (PMC4839064; doi:10.1186/s13012-016-0421-7)
Supplement: Supplementary file 4 — Study Characteristics. (PDF 136 kb) [file 13012_2016_421_MOESM4_ESM.pdf]

Appendix 4. Study Characteristics

First Author, Year	Country	Study Design	Funding	Study Period	KT Duration (Follow up)*	Site; Study setting	Organizational Context	Sample Size - Pxs overall (Drs)	No. of Arms	Defined Sustainability	Definition Cited	Sustainability Terminology [Root word]
Belardinelli, 2012	Italy	RCT	Research funding body	NR	522	single site; hospital, community	NR	123	2	No	NA	Long term, Sustain
Cheng, 2012	Taiwan	Cohort	Research funding body	2004 to 2009	209	multi-site; clinic	Nationwide compulsory universal health insurance program	189020 (3582)	2	No	NA	Long term, Continue
Rothschild, 2012 (CR: Martin, 2011)	USA	RCT	NR	NR	104	single site; clinic	University Medical Center	144	2	No	NA	Sustain, Adhere, Long term
Tamone, 2012	Italy	Cohort	Commercial organisation	2008 to 2010	78	multi-site; hospital	NR	780	2	No	NA	Persist, Long term
Baker, 2011	USA	Cohort	Commercial organisation	2006 to 2008	104	multi-site; home, clinic	3 Medical practices	3534	2	No	NA	NR
Barrera, 2011 (CR: Toobert, 2012)	USA	RCT	Research funding body	NR	104	single site; community	Health maintenance organization	279	2	Yes	sustainability of intervention effects	Maintain, Sustain, Support, Continue
Gibson, 2011	USA	Case-control	Commercial organisation	2006 to 2008	157	single site; community	Diabetes disease management program	2204	2	No	NA	Adhere
Kim, 2011 (CR: Kim, 2008)	USA	RCT	Governmental organisation, Other	NR	65	multi-site; community	Self-Help Intervention Program	397	2	Yes	sustainability of those intervention outcomes or long-term effects	Sustain, Long term
Chen, 2010 (CR: Gilmore, 2007; Chung, 2003)	USA	Cohort	Health-care provider organisation	1998 to 2007	209	multi-site; clinic	Commercial Preferred Provider Organization health plan	NR (NR)	2	No	NA	Sustain

Desouza, 2010	USA	Cohort	NR	2004 to 2008	104	single site; clinic	Shared medical appointment at diabetes clinic	56	2	No	NA	Maintain, Long term
Hughes, 2010	USA	RCT	Governmental organisation	NR	78	multi-site; hospital, home, community	Hospital and home	419	4	No	NA	Long term, Maintain
Huizinga, 2010	USA	RCT	Governmental organisation	2002 to 2006	104	single site; clinic	Urban area surrounding an academic medical centre	164	3	No	NA	Long term
Wisse, 2010	The Netherlands	RCT	NR	NR	104	single site; clinic	NR	61	2	No	NA	NR
Xian, 2010	USA	Cohort	Commercial organisation, Research funding body	2004 to 2007	157	multi-site; hospital	Continuous quality improvement program	NR (2878 hospitals)	2	Yes	sustained performance over time	Sustain
Chavannes, 2009	The Netherlands	Cohort	Mixed	NR	104	multi-site; clinic	2 primary health care centers	152	2	No	NA	Long term
Jia, 2009	USA	Cohort	Research funding body, Governmental organisation	NR	209	multi-site; clinic	4 Veterans Affairs medical centers	774	2	No	NA	Long term
Olson, 2009	USA	RCT	Research funding body	2005 to 2007	104	multi-site; clinic	Non-profit health maintenance organization	421	2	Yes	maintenance of treatment goals after discharge	Sustain, Maintain, Long term
Radziewicz, 2009	USA	RCT	NR	NR	78	NR	NR	NR	2	No	NA	Maintain, Adhere
Svetkey, 2009	USA	Cluster-RCT	Governmental organisation	2005 to 2007	78	multi-site; clinic	NR	574 (32)	4	No	NA	Sustain
van Wetering, 2009	Netherlands	RCT	Research funding body, Commercial organisation	2002 to 2006	104	multi-site; clinic, community	2 general hospitals	199	2	No	NA	Long term
Bocchi, 2008	Brazil	RCT	NR	1999 to 2006	129 (mean)	single site; hospital	Heart Institute	350	2	No	NA	Long term
Giannuzzi, 2008	Italy	RCT	Commercial organisation	2001 to 2005	157	multi-site; clinic	78 Italian cardiac rehabilitation centers	3241	2	No	NA	Maintain, Long term

Lawrence, 2008	US	Cohort	NR	2004 to 2006	74	multi-site; community	Longitudinal Adherence Treatment Evaluation program	155	2	No	NA	NR
Mildestvedt, 2008	Norway	RCT	Voluntary body	2000 to 2002	104	single site; chronic care	Rehabilitation Centre	176	2	No	NA	Long term
Berg, 2007	USA	Cohort	Commercial organisation	2000 to 2002	104	multi-site; clinic, home	Hospitalization or an emergency department visit	1220	2	No	NA	NR
Chin, 2007	USA	Cluster-RCT	Governmental organisation, Research funding body	1998 to 2002	52 to 104 (209)	multi-site; clinic, community	34 community health centers	2417 (34)	2	Yes	what happens over longer time periods [more than 1 year]- whether improvements in care can be sustained and if certain outcome goals may take more time to attain	
Del Sindaco, 2007	Italy	RCT	NR	2001 to 2004	104	multi-site; clinic, home	2 hospital heart failure clinics	173	2	No	NA	Sustain, Long term
Dennison, 2007	USA	RCT	Commercial organisation, Research funding body, Governmental organisation	NR	261	single site; clinic, home	High-risk, underserved young urban African American men	309	2	No	NA	NR
Getpreechaswas, 2007	Thailand	Cohort	Governmental organisation	2002 to 2006	157	multi-site; clinic	3 primary care units	465	3	Yes	NA	Sustain
Hess, 2007	USA	NRCT	Mixed	2003 to 2005	61	single site; community	Black-owned barbershops	308	2	No	NA	Sustain
Bailie, 2006	Australia	Cohort	Governmental organisation, Research	1995 to 2002	339	multi-site; clinic, communit	Community health centers	98	2	Yes	maintenance of health	Sustain, Maintain, Mesurable gain

			funding body			y					benefits/ou tcomes	
Inglis, 2006	Australia	RCT	Charitable trust	1995 to 1998	522	single site; clinic	Tertiary referral center	297	2	No	NA	Long term, Adhere
Montero, 2005	Spain	RCT	NR	NR	522	single site; hospital	Hospital	180	2	No	NA	Long term
Phillips, 2005	USA	RCT	Governmental organisation	1999 to 2002	157	single site; clinic	Multispecialt y group	4138	4	No	NA	NR
Bakitas, 2004	USA	Cohort	Voluntary body	1998 to 2001	78	multi-site; hospital	3 Medical practices	380	2	No	NA	Continue, Plan
Fihn, 2004	USA	RCT	Governmental organisation	1997 to 1999	104	multi-site; clinic	7 Veterans Affairs medical centers	9019	2	No	NA	Sustain
Jovanovic, 2004	USA	RCT	Health-care provider organisation, Governmental organisation	1995 to 1999	157	multi-site; clinic	3 clinical sites	358	2	No	NA	NR
Behnke, 2003	Germany	RCT	Governmental organisation, Research funding body	NR	78	multi-site; hospital, home	NR	26	2	No	NA	Long term, Sustain
Gaede, 2003	Denmark	RCT	NR	1993 to 2001	407	single site; clinic	Project team at Diabetes Center	160	2	No	NA	Long term
Gary, 2003	USA	RCT	Research funding body, Health-care provider organisation	1995 to 1999	104	single site; clinic, home	Predominatel y African American, inner-city community	186	4	No	NA	Surpass
Allen-Ramey, 2002	USA	Cohort	Health-care provider organisation	1993 to 1995	104	multi-site; clinic, communit y	16 managed care organizations	3013	2	No	NA	Maintain
Froehlich, 2002	USA	Cohort	NR	1993 to 1998	60 to 104	multi-site; hospital	NR	300	3	No	NA	Sustain, Long term
Baker, 2001	USA	Cohort	NR	1998 to 1999	95	multi-site; clinic	Multispecialt y group practice	13325 (190)	2	No	NA	Sustain
Coleman, 2001	USA	RCT	Research funding body, Commercial organisation	1995 to 1996	104	multi-site; clinic	Group-model health maintenance organization	295	2	No	NA	NR
Hedges, 2000	USA	RCT	Governmental organisation, Commercial organisation	NR	78	multi-site; communit y	44 hospitals	3013	2	No	NA	Sustain

Rowley, 2000	Australia	Cohort	Governmental organisation	1993 to 1997	104	single site; community	Looma Healthy Lifestyle (diabetes programme)	49	2	No	NA	NR
Skinner, 2000	USA	Quasi-RCT	Research funding body	1995 to 1997	157	multi-site	2 sites affiliated with the System to Assure Elder Services (STAES) program	152	2	No	NA	Sustain, Adhere
Stroebel, 2000	USA	Cohort	NR	1997 to 1999	117	single site; clinic	Multispecialty group practicie	867	2 (40)	No	NA	Sustain
Daniel, 1999	Canada	CBA	Governmental organisation, Charitable trust	1994 to 1996	70	multi-site; community	3 matched communities	207	2	No	NA	Limited time
Grosbois, 1999	France	Cohort	NR	NR	78	single site; clinic, home	Rehabilitation hospita	58	4	No	NA	Long term, Sustain
Higginbotham, 1999	Australia	Cohort	Governmental organisation	1984 to 1994	522	single site; community	Coalfields Healthy Heartbeat (CHHB) community action program	NR	2	Yes	sustainability of activities	Long term, Sustain
Pill, 1998	UK	RCT	Research funding body	1993 to 1996	104	multi-site; clinic	30 practices	190	2	No	NA	Sustain
Corkery, 1997	USA	RCT	NR	NR	4 to 70	single site; clinic	Nurse-managed diabetes management clinic (	64	2	No	NA	Sustain, Long
Kelso, 1996	USA	NRCT	NR	1992 to 1995	104	multi-site; clinic	From local teaching hospitals, referrals, and through local public service announcements	39	2	No	NA	Long term
Reichard, 1996	Sweden	RCT	NR	NR	522	single site; clinic	NR	102	2	Yes	clarify whether the results reported earlier remain	Long term

											when the treatment groups have returned to routine care	
Erfurt, 1990	USA	Cohort	Governmental organisation, Commercial organisation	1985 to 1988	157	multi-site; communit y	14 manufacturin g plalnts	7804	4	No	NA	Long term, Sustain
Perk, 1989	Sweden	Cohort	NR	1983 to 1985	104 (>209)	single site; hospital	District Hospital	40	2	No	NA	Long term
Hopper, 1984	USA	RCT	NR	1979 to 1980	78 (NR)	single site; clinic	NR	227	2	No	NA	NR
Weiss, 1984	USA	RCT	Health-care provider organisation	1978 to 1983	157	multi-site; hospital, chronic care	Long-Term Care Demonstratio n Program	338	2	No	NA	NR
Ambrosio, 1983	Italy	Cohort	NR	NR	261	single site; communit y	District Hospital	5856	2	No	NA	Long term
Morisky, 1983	USA	RCT	Research funding body	1974 to 1979	261	multi-site; clinic	Internal medical clinic	400	8	No	NA	Comply, Adhere
Krishan, 1979	USA	Cohort	Health-care provider organisation, Charitable trust	1974 to 1975	52 to 104	multi-site; communit y	3 non-contiguous rural communities	1392	3	No	NA	NR

Abbreviations: CR – Companion Report **NR** – Not reported; **RCT** – Randomised controlled trial; **NA** – Not available; **CBA** – Controlled before-after; **NRCT** – Non-randomized controlled trial
