# Supplementary material for: Sustainability of knowledge translation interventions in healthcare decision-making: a scoping review
Source: Implement Sci. 2016 Apr 21;11:55. doi: 10.1186/s13012-016-0421-7 (PMC4839064; doi:10.1186/s13012-016-0421-7)
Supplement: Supplementary file 5 — Patient Characteristics. (PDF 200 kb) [file 13012_2016_421_MOESM5_ESM.pdf]

Appendix 5. Patient Characteristics

First Author, Year	End users	Mean Age-Pxs (yrs)	Age Range	% Female	Chronic Disease	Co-morbidity/Risk Factor	History of Treatment Usage	Concomitant Therapies	Other Inclusion Criteria
Allen-Ramey, 2002	Patients/public; Clinicians	46.6	≥18	70.9	Asthma	NR	70-80% use inhaled corticosteroids	NR	age 18 years or older as of September 1, 1993; enrollment in the MCO at the time of sampling; enrollment in the MCO at the time of sampling
Ambrosio, 1983	Patients/public; Clinicians	NR	20-64	48	Hypertension	NR	NR	NR	NR
Bailie, 2006	Patients/public; Clinicians	NR	NR	NR	Cardiovascular disease and Renal disease	Diabetes, Hypertension, and Albuminuria	NR	NR	NR
Baker, 2001	Clinicians	60	NR	52	Diabetes	Cardiac comorbidity, Renal comorbidity, and Eye comorbidity	oral agent, insulin	NR	Adult patients older than 18 years were identi-fied for inclusion in rhe DCMSS if in rhe past 24 months rhey had had two encounters wirh a medical group physician for their diabetes care (two encounters with a corresponding ICD-9-CM diagnostic code of 250) or a claim for either insulin or an oral hypoglycemia drug. To ensure the most complete information possible for each patient, we limited rhe study population to those enrolled in the medical group's affiliated 1, 1998; this resulted in the identification of 17,328 patients with diabetes. We further limited the sample to patients who were continuously enrolled in the HMO from November 1, 1997, to October 31, 1999, thereby including only established HMO patients for whom complete information was available in both the baseline and study year. The resulting evaluation sample consisted of 13,325 adult HMO patients with diabetes who were aligned to 190 PCPs practicing in 31 ambulatory care clinics. Table 1 (p 182) presents the sociodemographic and clinical characteristics of the study cohort, along with the diabetes-related routine monitoring and screening service receipt among the population for the baseline year.
Baker, 2011	Patients/public; Clinicians	NR	NR	NR	Diabetes, COPD, and CHF	NR	NR	NR	A primary goal of CMS's selection algorithm was to identify patients who had evidence of congestive heart failure, chronic obstructive pulmonary disease, or diabetes mellitus—three conditions targeted by the CMS demonstration project. The selection algorithm required that intervention patients receive most of their care at the study clinics. It identified appropriate

									high-risk, high-cost patients while excluding others who would not be candidates for interacting with the program.
Bakitas, 2004	Patients/public	NR	NR	NR	Cancer	NR	NR	NR	1. Diagnosis of stage IV, metastatic, or recurrent breast cancer; stage IIIB/IV non-small–cell or extensive stage small cell lung cancer; unresectablex colon, pancreatic, biliary, hepatic cancer;<br>2. Able to speak and read or understand English;<br>3. Age 18 or older;<br>4. Receiving oncology care from the site
Barrera, 2011	Patients/public	61	39-74	100	Diabetes	NR	NR	NR	NR
Behnke, 2003	Patients/public	66.3	NR	23.1	COPD	NR	22 patients taking theophylline and/or oral corticosteroids	NR	NR
Belardinelli, 2012	Patients/public	59	included adults and elderly	21.9	CHF	NR	angiotensin-converting enzyme; angiotensin II antagonist; beta-blocker; digitalis; diuretic; statins	NR	clinical stability for 3 months before enrollment, left ventricular ejection fraction (LVEF) of less than 40%, and ability to exercise
Berg, 2007	Patients/public; Clinicians	73.9	≥65	62	Diabetes	Hypertension, Coronary artery disease, COPD, Diabetic retinopathy, and Peripheral vascular disease	ACE-inhibitors, beta-blockers, antihypertnesives, diuretics, blood boucose regulators	NR	Patients who opted out before having an initial assessment were included in the intervention group to reflect an intent to treat evaluation approach for the contacted members. Members of a MedicareChoice health plan residing in Ohio, Indiana, or Kentucky who had a hospitalization or an emergency de- partment visit for which diabetes was one of the diagnoses as determined by medical claims review in the past year;<br>• Members from the above who are not en- gaged in a local formal diabetes program;<br>• Members aged 65 years or older • Health plan enrollment at least 3 months prior to being contacted to be in the study and at least 3 months of health plan partici- pation after being contacted to be in the study . • Patients residing in a long-term skilled nurs- ing facility (SNF), participating in a hospice program, or who were identified as having end-stage renal disease, dialysis, transplants, AIDS, claims costs □$100,000, or malignant cancer were excluded. Long-term SNF stays, defined as longer than 30 days, were used as an exclusion criterion as a telephonic inter-

									vention was considered impractical.<br>• Patients who opted out before having an initial assessment were included in the intervention group to reflect an intent to treat evaluation approach for the contacted members.
Bocchi, 2008	Patients/public	50.7	≥18	31.2	HF	Diabetes, COPD, Alcoholic, Chagasic, and Hypertensive	Amiodarone, Amlodipine, ARA, Oral anticoagulant, ACEI, beta-Blocker, Spironolactone, Hydralazine, Nitrates, Digoxin, Diuretics, Furosemide, Statin, Oral antidiabetic therapy, Insulin, Oral thyroid hormone	Standard follow-up medical visits for the intervention and control groups were performed during the study period by the same ambulatory cardiology team, which was not informed of the randomization. The scheduled interval between routine ambulatory evaluations was 3 to 4 months. Also, the ambulatory team did not participate in any step of the study. The ambulatory cardiology team was oriented to follow Brazilian Guidelines and standard treatment of the Heart Failure Clinics in the management of patients. Complementary	irreversible chronic HF of at least 6 months' duration

								follow-up visits were carried out, depending on each patient's needs and on the decision of the attending cardiologist. When nurses detected noncompliance or worsening of the clinical condition, an unscheduled visit to the patient's attending cardiologist could be proposed.	
Chen, 2010	Clinicians	NR	NR	NR	HF, and diabetes	NR	NR	NR	NR
Cheng, 2012	Clinicians	63.9	≥18	56.7	Diabetes	NR	NR	NR	Patients with diabetes who had been enrolled in the nationwide P4P program in 2005 were defined as the intervention group (N = 20,934). The index date for each patient was defined as the date that they were first enrolled in the P4P program between January 1, 2005 and December 31, 2005. To examine the long-term effects of the P4P program, we created 2 sets of samples: one for all of the participants regardless of their enrollment status in the subsequent years and the other for those who participated and stayed in the program throughout the observation period from 2005 to 2009.
Chin, 2007	Patients/public; Clinicians	56.2	18-75	63	Diabetes	Hypertension, MI, Retinopathy, Neuropathy, and Peripheral vascular disease	ACE inhibitor, aspirin, etc.	NR	
Coleman, 2001	Patients/public; Clinicians	74.05	NR	59	Asthma, COPD, CHF, Diabetes, Heart disease, and Hypertension	NR	NR	NR	were patients of a participating physician. older adults with chronic illness and a history of frequent utilization of outpatient services, patients of a participating physician
Corkery, 1997	Patients/public; Clinicians	52.8	21 to 76	74	Diabetes	NR	NR	NR	NR

Daniel, 1999	Patients/public	47.1	18-80	65.7	Diabetes	Diabetes, and Family history of diabetes	NR	NR	NR
Del Sindaco, 2007	Patients/public; Clinicians	77.5	≥ 70	48	HF	Hypertension, Diabetes, MI, COPD, and Valvular heart disease	ACE-inhibitors, angiotensin receptor blockers, beta-blockers, furosemide, spironolactone, digoxin, amiodarone, nitrates anticoagulants	All patients were discharged on optimised therapy and received a detailed summary and a standardised educational programme focused on a low-salt diet and drug therapy, self-monitoring of blood pressure and symptoms, daily weight, smoke cessation, fluid intake and daily physical activity. An ad-hoc translated and validated local version of the European Heart Failure Self-Care Behaviour Scale was administered in order to evaluate the baseline level of self-care.	discharged home after a hospitalisation due to heart failure, defined as an admission in New York Heart Association (NYHA) functional class III – IV of at least 24 h requiring specific intravenous therapy.
Dennison, 2007	Patients/public; Clinicians	41	21-54	0	Hypertension	Smoker, High-salt diet, Diabetes, Hyperlipidemia, and Obesity	antihypertensives	NR	
Desouza, 2010	Patients/public	66.4	NR	NR	Diabetes	NR	85% insulin, 15% two or more oral medications	NR	Patients were included in the study if their A1c at discharge was <7 and had subsequent outcome data avail- able every 6 months for a period of 2 years.
Erfurt, 1990	Patients/public	39-43	39-43	NR	Hypertension	Obesity, and Smoker	NR	NR	NR

Fihn, 2004	Clinicians	65	≥65	3	Coronary artery disease, Hypertension, Diabetes, COPD	Hypertension, Diabetes, Angina, Depression, and COPD	NR	NR	All enrolled patients who visited their assigned primary care providers at least once during the prior year were eligible for the study.
Froehlich, 2002	Clinicians	67.3	NR	25	Cardiovascular diseases	Diabetes, CHF, MI, Occlusive aortic disease, and Aortic aneurysm	NR	NR	NR
Gaede, 2003	Patients/public	55.1	NR	26	T2DM, and Cardiovascular disease	T2DM, Microalbuminuria, Neuropathy, Hypertension, and Smoker	ACE inhibitor, Angiotensin II–receptor antagonist, Diuretic, Calcium-channel blocker, Beta-blocker	NR	NR
Gary, 2003	Patients/public; Clinicians	59	35-75	77	T2DM	Hypertension, Hyperlipidemia, and Smoker	insulin, oral hypoglycemic agents, blood pressure medication, cholesterol medication	NR	Eligibility for the trial was determined by medical chart review and two screening visits. Age (35–75), African American ancestry, presence of type 2 diabetes (as indicated by physician di- agnosis), and residence in one of seven East Baltimore zip codes were preliminary eligibility criteria. In addition, all participants attended either the Johns Hopkins Outpatient Center or the East Baltimore Medical Center for primary care within the previous year.
Getpreechaswas, 2007	Patients/public; Clinicians	NR	18-59	63.6	Hypertension	Smoking, and Alcohol consumption	NR	NR	NR
Giannuzzi, 2008	Patients/public; Clinicians	57.9	≤ 75	13.7	MI	Diabetes, Hypertension, Hypercholesterolemia, Overweight, and Smoker	aspirin, other anitplatelet agents, calcium channel blockers, beta blockers, nitrates, diuretics, ACE inhibitors, oral antidiabetic drugs, insulin, lipid-lowering agents, PUFA	NR	Patients enrolled in the GOSPEL Study who had had a recent MI (within 3 months) were routinely referred to a cardiac rehabilitation center, residential or ambulatory, irrespective of revascularization procedures received after the index event
Gibson, 2011	Patients/public; Clinicians	47.75	NR	41.2	Diabetes	NR	NR	NR	NR
Grosbois, 1999	Patients/public	62.2	NR	19	COPD	NR	NR	NR	dyspnea on exertion limiting activities of daily living, postbronchodilator forced expiratory volume in one second (FEV1) less than 70% of predicted values, respiratory stable condition with optimal drug management, and no evidence of ischemic heart disease or musculoskeletal disorders.
Hedges, 2000	Patients/public; Clinicians	NR	>30	36.3	AMI	Coronary heart disease	NR	NR	Patients transferred from a study hospital for further evaluation or treatment were included

									when data abstraction could be completed at the second hospital.
Hess, 2007	Patients/public	NR	NR	0	Hypertension	NR	NR	NR	NR
Higginbotham, 1999	Patients/public	NR	NR	NR	Coronary heart disease	Smoking, Physical inactivity, BMI, Diastolic blood pressure, and Systolic blood pressure	NR	NR	NR
Hopper, 1984	Patients/public; Clinicians	46.5	NR	73.5	Diabetes	NR	NR	NR	NR
Hughes, 2010	Patients/public	71.1	59-91	86.6	Osteoarthitis	Hypertension, diabetes, and Cardiovascular disease	NR	NR	NR
Huizinga, 2010	Patients/public	55.1	18-75	44	Diabetes	Obesity	insulin, metformin, sulfonylurea, thiazolidinedione, meglitinides	NR	Patients who had been referred to the diabetes DIP by their primary care physician or endocrinologist for poor glycaemic control (HbA1c >8%) and who demonstrated significant improvement (≥1% decline in HbA1c) during the DIP were recruited for the study. The DIP is a usual-care diabetes management programme offered within the academic medical centre and consists of a physician-led team, including a nurse practitioner, a registered dietitian and a diabetes nurse, and has been described previously. English speaking
Inglis, 2006	Patients/public; Clinicians	75	NR	44	Chronic heart failure	Hypertension, MI, Diabetes, and Chronic airways disease	NR	NR	discharged to home, and had a diagnosis of CHF (determined by a cardiologist) and a history of 1 admission for acute heart failure.
Jia, 2009	Patients/public	67.6	NR	1.7	Diabetes	CHF, COPD, Hypertension, Asthma, and Angina	NR	NR	NR
Jovanovic, 2004	Patients/public; Clinicians	57	≥18	71.9	Diabetes	Smoking , Ethnic minority, and Low education < high school 40%	NR	NR	individuals 18 years or older who had type 2 diabetes of at least 1 year duration
Kelso, 1996	Patients/public	38.2	≥18	79.5	asthma	African American	inhaled corticosteroid, oral corticosteroids, theophylline	NR	Inclusion criteria included being an African-American adult (>= 18 years of age), meeting NIH 1 criteria for moderate to severe asthma, and frequent ED visits (>= 5 in the previous 2 years or 3 in the previous 12 months) or hospitalizations (>= 2 during the previous 2 years), or at least 1 intensive care unit (ICU) admission in the previous 2 years.
Kim, 2011	Patients/public; Clinicians	51.9	38-64	52.9	Hypertension	No job , Other disease, Family history of	blood pressure medication	NR	SBP ≥140 mm Hg and/or DBP ≥90 mm Hg on two separate occasions or taking anti-hypertensive medication, and self-identification

						hypertension, and No medical insurance			as a Korean American.
Krishan, 1979	Patients/public; Clinicians	NR	30-69	NR	Hypertension	NR	NR	NR	referral for care from the cnetral and househld screening was recommended in persons whose levels were at or above 160mm Hg systolic or 95mm Hg diastolic, based on the average of the seconde and third readings. Patients who were already receiving treatement wer advised to contimue care with their local physicisans (or, in Wababsha, if they preferred, at the Community Hypertension Clinic).
Chavannes, 2009	Patients/public; Clinicians	63	NR	37	COPD	NR	NR	NR	NR
Lawrence, 2008	Patients/public; Clinicians	NR	18-65	51.6	Cardiovascular disease, and diabetes	NR	NR	NR	Eligibility for the DM programs was determined using med- ical and pharmacy claims data to identify patients with chronic conditions.
Montero, 2005	Patients/public; Clinicians	51.45	<65	0	AMI	Diabetes, Dyslipidemia, and Smoking	NR	NR	diag- nosed with AMI and admitted to the coronary care unit of our hospital. Enrolments were based on the Ameri- can Association of Cardiovascular and Pulmonary Re- habilitation inclusion criteria12: age <65 years, low- risk (hospital course without complications, absence of signs of myocardial ischemia, functional capacity >7 metabolic equivalent time [MET], ejection fraction >50%, and absence of severe ventricular arrhythmias).
Mildestvedt, 2008	Patients/public	56	NR	22	Coronary heart disease	Smoking	NR	NR	NR
Morisky, 1983	Patients/public	53.75	NR	70	Hypertension	Stroke or MI, Angina or CHF, Arteriosclerotic cardiovascular disease, Diabetes mellitus, and Hypertension	NR	NR	NR
Olson, 2009	Patients/public; Clinicians; Health-care managers	72	NR	26	MI	Hypertension	NR	NR	Patients identified for inclusion in the study were those who required only yearly follow-up per the CPCRS treatment protocol. Patients had to have at least 2 consecutive LDL-C and non–high-density lipoprotein cholesterol (non–HDLC) values at goal; the most recent value had to have been recorded within 6 months prior to study enrollment. There was no prespecified required time frame between the 2 FLPs for eligibility. The LDL-C and non–HDL-C goals were defined as <100 mg/dL and <130 mg/dL, respectively, for all patients except those with diabetes, multivessel coronary disease, at least 1 recurrent coronary

									event, or current smokers. For these patients, the LDL-C and non–HDL-C goals were <70 mg/dL and <100 mg/dL, respectively. Patients also were required to have controlled blood pressure within 6 months prior to study enrollment. The blood pressure goal was defined as <140/90 mm Hg for all patients except for those patients with diabetes or chronic kidney disease, for which the goal was <130/80 mm Hg.
Perk, 1989	Patients/public; Clinicians	58.4	45-64	10	MI	Hypertension, and Smoking	cadiovascular drugs	NR	Pts regularly attending LTP (defined as attending at least 3 out of 5 trainning sessions during a 5-month period)
Phillips, 2005	Clinicians	59	NR	67	T2DM	NR	NR	NR	NR
Pill, 1998	Patients/public; Clinicians	58.1	NR	50	Diabetes	Hypertension	tables for diabetes, hypertension medication	NR	aged 18-70, diagnoed > 1yr, glycosylated haemoglobin >9% at last reading, life expectancy range of at least 3 yrs.
Radziewicz, 2009	Patients/public; Clinicians	NR	NR	NR	Cancer	NR	NR	NR	NA
Reichard, 1996	Patients/public; Clinicians	31	NR	NR	Diabetes	Serious retinopathy, Visual deterioration, Nephropathy, and Smoking	subcutaneous insulin, anti-hypertensive medication	NR	NR
Rothschild, 2012	Patients/public; Clinicians	53.7	NR	67.4	T2DM	Hypertension	NR	NR	Eligible participants had to be Mexican-American, participants were required to be using at least one oral medication to control their diabetes, participants were required to have health insurance or to be enrolled as a patient at a free clinic or public facility at the time of randomization.
Rowley, 2000	Patients/public	46.9	NR	69.4	Diabetes	Obesity	NR	NR	NR
Skinner, 2000	Patients/public	73	NR	100	Cancer	NR	NR	NR	participants were women aged 45 to 69 at entry who had at least one risk factor for breast cancer (at least two during the Vanguard Phase) based on family history, age at first birth, and benign breast biopsies and who had a negative clinical breast exam and mammogram. All interested participants were screened to ensure that they were not already consuming a low-fat diet and that they were willing to make major dietary changes if randomized to the intervention group
Stroebel, 2000	Patients/public; Clinicians	67.3	51-80	60	Hypertension	NR	NR	NR	NR
Svetkey, 2009	Patients/public; Clinicians	60.5	adults at leats 25 years old	47	Hypertension	Obesity	NR	NR	NR

Tamone, 2012	Patients/public; Clinicians	73.6	65-82	92.3	Severe osteoporosis	NR	NR	calcium and vitamin D	Women and men who presented the criteria for prescription of teriparatide or PTH(1–84). All consenting patients who started teriparatide or PTH(1–84) from April 2008 to April 2010 were enrolled in the educational and follow-up program (intervention group).
van Wetering, 2009	Patients/public; Clinicians	66.5	57-76	29	COPD	Smoking, and Obesity	NR	NR	Patients were recruited when 1) having an impaired exercise capacity, defined as peak work load (Wmax) during incremental cycle ergometry of less than 70 percent of the predicted normal value, 2) having GOLD stage 2 or 3 COPD and 3) being able and willing to participate in a community-based program.
Weiss, 1984	Patients/public; Clinicians	80	66-99	69.5	Chronic ill patients (cardiovascular, gastrointestinal and liver, genitourinary, nervous system, bone and joint, respiratory, arthritis and rheumatic, others)	NR	NR	NR	any person 65 years of age or older who has Medicare A and B coverage only (not Medicaid coverage), resides within a certain geographic area of San Francisco, and has health or social needs that make it difficult to live or function independently.
Wisse, 2010	Patients/public	52.9	NR	38	Diabetes	Obesity, MI, and Cerebral vascular incident	Anti-hypertensive drug, Statin, Anticoagulants/aspirin, Exogenous insulin, Metformin	NR	NR
Xian, 2010	Patients/public; Clinicians; Health-Care managers	NR	NR	NR	Coronary artery disease	NR	NR	NR	they performed a data analysis using the Centers for Medicare and Medicaid Services (CMS) Hospital Compare database to compare the guideline adherence between GWTG-CAD hospitals and hospitals not participating in GWTG-CAD for 3 years and evaluated the independent effect of the GWTG-CAD program.
**Abbreviations: NR** – Not reported; **COPD** - Chronic obstructive pulmonary disease; **CHF** - Congestive heart failure; **HF** – Heart failure; **MI** – Myocardial infarction; **AMI** – Acute myocardial infarction; **T2DM** – Type 2 Diabetes									
