# Supplementary material for: Sustainability of knowledge translation interventions in healthcare decision-making: a scoping review
Source: Implement Sci. 2016 Apr 21;11:55. doi: 10.1186/s13012-016-0421-7 (PMC4839064; doi:10.1186/s13012-016-0421-7)
Supplement: Supplementary file 6 — KT Interventions. (PDF 181 kb) [file 13012_2016_421_MOESM6_ESM.pdf]

Appendix 6. KT Interventions

First Author, Year	Treatment	Interventions (QI)	Level	Target	Fidelity[a]	Adaptation[b]
Allen-Ramey, 2002	Managed Care Organization Programs	Clinician education, Audit and feedback, Financial incentives, Education of patients, Reminders, Case management, Promotion of self-management	Health care providers; Patients; Health system	Physician + Nurse; Patient	No	Yes
	Reference, other Managed Care Organization Programs	Clinician education, Audit and feedback, Financial incentives, Education of patients, Reminders, Case management, Promotion of self-management	Health care providers; Patients; Health system	Physician + Nurse; Patient		
Ambrosio, 1983	After intervention	Team changes, Electronic patient registry, Education of patients	Health system ; Patients	Community centre; General public	No	No
	Before intervention	Usual care/Control	NR	NR		
Bailie, 2006	Specialized chronic disease treatment program (Pre-handover)	Team changes, Case management, Electronic patient registry	Health system	Patient; Community Centre	No	Yes
	Incorporation of program activities into routine service delivery (Post-handover)	Reminders, Clinician education	Health care providers	Health centres		
Baker, 2001	Diabetes Care	Electronic patient registry, Audit	Health system; Health	Clinicians; Physicians	No	No

	Management Support System	and feedback, Clinician education	care providers			
	No-Diabetes Care Management Support System	Usual care/Control	NR	NR		
Baker, 2011	Health Buddy Program	Case management, Facilitated relay of information to clinicians, Promotion of self-management, Education of patients	Health system; Patients	Patient; Care manager	No	No
	Matched	Usual care/Control	NR	NR		
Bakitas, 2004	Demonstration project	Case management, Education of patients, Team changes, Promotion of self-management	Health system; Patients	Patient; Patient + Family	No	No
	Control	Usual care/Control	NR	NR		
Barrera, 2001	Mediterranean Lifestyle Program	Promotion of self-management, Education of patients, Financial incentives, Motivational interviewing, Reminders	Patients	Patient	No	No
	Usual care	Usual care/Control	NR	NR		
Behnke, 2003	Training	Usual care/Control, Promotion of self-management, Reminders	Patients	Patient	No	No
	Control	Usual care/Control, Reminders	Patients	Patient		
Belardinelli, 2012	Exercise training protocol	Education of patients	NR	Patient	No	No
	Control	Education of patients	NR	Patient		
Berg, 2007	Telephonic nursing disease management program	Case management, Promotion of self-management, Reminders, Education of patients	Health system; Health care providers; Patients	Patient; Physician	No	No
	Matched	Usual care/Control	NR			
Bocchi, 2008	Disease Program Management	Team changes, Education of patients, Case management, Promotion of self-management	Health system; Patients	Patients	No	No
	Usual care	Usual care/Control	NR	NR		
Chin, 2007	Standard intensity	Team changes, Continuous QI, Promotion of self-management, Audit and feedback, Electronic patient registry, Clinician	Health system; Health care providers; Patients	Community health centres; Patient	No	No

		education, Team changes				
	High intensity	Team changes, Continuous QI, Promotion of self-management, Audit and feedback, Electronic patient registry, Clinician education, Team changes	Health system; Health care providers; Patients	Community health centres; Patient		
Chen, 2010	Pay-for-Performance program	Audit and feedback, Financial incentives	Health care providers	Physician	No	No
	Comparison	Usual care/Control	NR	NR		
Cheng, 2012	Pay-for-Performance program	Financial incentives	Health care providers	Physician	No	No
	Matched	Usual care/Control	NR	NR		
Coleman, 2001	Intervention	Promotion of self-management, Team changes, Social support, Education of patients, Case management	Health system; Patients	Patient	No	No
	Control	Usual care/Control	NR	NR		
Corkery, 1997	Community health worker	Education of patients, Social support, Promotion of self-management, Reminders, Case management	Health system; Patients	Patient + Family; Patient	No	No
	No- Community health worker	Education of patients, Promotion of self-management,	Patients	Patient + Family; Patient		
Daniel, 1999	Intervention	Education of patients, Promotion of self-management, Social support	Patients	Community	No	Yes
	Control	Usual care/Control	NR	NR		
Del Sindaco, 2007	Disease management program	Education of patients, Team changes, Case management	Health system; Patients	Patient	No	No
	Usual care	Usual care/Control	NR	NR		
Dennison, 2007	More intensive comprehensive educational-behavioral-pharmacologic intervention by a nurse practitioner/community	Team changes, Social support, Education of patients, Reminders, Case management	Health system; Patients	Patient	No	No

	health worker/physician team					
	Less intensive education + referral intervention	Team changes, Social support, Education of patients, Reminders, Case management	Health system; Patients	Patient		
Desouza, 2010	Group diabetes clinic	Team changes, Education of patients, Case management	Health system; Patient	Patient	No	No
	Primary care provider	Usual care/Control	NR	NR		
Erfurt, 1990	Site 1: Control + Wellness Screening	Team changes	Health system	NR	No	No
	Site 2: Wellness Screening + Health education	Team changes, Education of patients	Health system; Patients	Employee; Patient		
	Site 3: Wellness Screening + Health education + Follow-up counseling	Team changes, Education of patients Case management, Promotion of self-management	Health system; Patients	Employee; Patient		
	Site 4: Wellness Screening + Health education + Follow-up counseling + Plant Organization	Team changes, Education of patients Case management, Promotion of self-management, Social support	Health system; Patients	Employee; Patient		
Fihn, 2004	Ambulatory Care Quality Improvement Project	Audit and feedback, Clinician education	Health care providers	Clinicians	No	Yes
	No-Ambulatory Care Quality Improvement Project	Usual care/Control	NR	Clinicians/Intervention provider		
Froehlich, 2002	Late post guideline implementation	Team changes, Clinician education	Health system; Health care providers	Clinicians	No	No
	Immediate post guideline implementation	Team changes, Clinician education	Health system; Health care providers	Clinicians		
	Control	Usual care/Control	NR	NR		
Gaede, 2003	Conventional therapy	Usual care/Control	NR	NR	No	No
	Intensive therapy	Education of patients, Promotion of self-management	Patients	Patient		
Gary, 2003	Usual care	Usual care/Control, Education of	Patients	Patient	No	No

		patients				
	Usual care + Nurse case manager	Usual care/Control, Education of patients, Case management, Promotion of self-management	Health system; Patients	Patient		
	Usual care + Community health worker	Usual care/Control, Education of patients, Case management, Promotion of self-management, Social support	Health system; Patients	Patient		
	Usual care + Nurse case manager + Community health worker team	Usual care/Control, Case management, Promotion of self-management, Social support	Health system; Patients	Patient		
Getpreechaswas, 2007	Carried out by health personnel + Village health volunteers + Family health leaders	Education of patients, Team changes, Promotion of self-management, Clinician education, Continuous QI	Health system; Health care providers; Patients	Patients; Health personnel	No	No
	Carried out by health personnel + Village health volunteers	Education of patients, Team changes, Promotion of self-management, Clinician education, Continuous QI	Health system; Health care providers; Patients	Patients; Health personnel		
	Carried out by health personnel	Education of patients	Patients	Patient		
Giannuzzi, 2008	Intervention	Team changes, Education of patients, Promotion of self-management	Health system; Patients	Patient	No	No
	Control	Usual care/Control	NR	NR		
Gibson, 2011	Disease Management Program	Education of patients, Case management, Promotion of self-management, Financial incentives	Health system; Patients	Patient; Provider of care	No	No
	No- Disease Management Program	Usual care/Control	NR	NR		
Grosbois, 1999	Rehabilitation program, continued structured (supervised) exercise maintenance 2x/week	Education of patients, Promotion of self-management	Patients	Patient	No	No
	Rehabilitation program, continued	Education of patients, Promotion of self-management	Patients	Patient		

	structured (supervised) exercise maintenance 1x/week					
	Rehabilitation program, daily exercise maintenance	Education of patients, Promotion of self-management	Patients	Patient		
	Rehabilitation program, stopped after the program	Education of patients, Promotion of self-management	Patients	Patient		
Hedges, 2000	Educational program	Education of patients, Social support, Clinician education, Promotion of self-management	Patients	Patient + General public; Patient; Health professionals	No	No
	Control	Usual care/Control	NR	NR		
Hess, 2007	Barber Intervention	Financial incentives, Education of patients, Audit and feedback, Team changes	Health system; Patients	Barber; Patient/customers	No	No
	Barber Control	Education of patients	Patients	Patient/customers		
Higginbotham, 1999	Healthy heart support	Promotion of self-management, Education of patients	Patients	Patient	No	No
	Control	Usual care/Control	NR	NR		
Hopper, 1984	Home health aides	Education of patients, Team changes	Health system; Patients	Patient	No	No
	Control	Usual care/Control	NR	NR		
Hughes, 2010	Exercise program, negotiated maintenance with telephone reinforcement	Education of patients, Promotion of self-management, Motivational interviewing, Case management, Reminders	Health system; Patients	Patient	No	No
	Exercise program, negotiated maintenance with no telephone reinforcement	Education of patients, Promotion of self-management, Motivational interviewing	Patients	Patient		
	Exercise program, mainstreamed to facility-based exercise program with telephone	Education of patients, Promotion of self-management, Motivational interviewing, Reminders	Health system; Patients	Patient		

	reinforcement					
	Exercise program, mainstreamed to facility-based exercise program with no telephone reinforcement	Education of patients, Motivational interviewing	Patients	Patient		
Huizinga, 2010	Routine follow-up	Usual care/Control	NR	NR	Yes	No
	Routine follow-up + quarterly telephone contact	Team changes, Promotion of self-management, Case management, Motivational interviewing	Health system; Patients	Patient		
	Routine follow-up + monthly telephone contact	Team changes, Promotion of self-management, Case management, Motivational interviewing	Health system; Patients	Patient		
Inglis, 2006	Home based intervention	Team changes, Case management, Education of patients, Promotion of self-management	Health system; Patients	Patient	No	No
	Usual care	Usual care/Control, Team changes	Health system; Patients	Patient		
Jia, 2009	Care Coordination Home Telehealth program	Case management, Facilitated relay of information to clinicians, Team changes, Reminders	Health system; Patients	Patient	No	No
	Control	Usual care/Control	NR	NR		
Jovanovic, 2004	Diabetes case management	Case management, Education of patients, Promotion of self-management, Facilitated relay of information to clinicians, Financial incentives	Health system; Patients	Patient; Primary care provider	No	No
	Traditional primary care treatment	Usual care/Control	NR			
Kelso, 1996	Educational Interventions	Education of patients, Promotion of self-management	Patients	Patient	No	No
	Control	Usual care/Control	NR	NR		

Kim, 2011a	Participants assigned to the in-class to receive High blood pressure-related education	Education of patients, Promotion of self-management	Patients	Patient	No	No
	Participants assigned to the mail education to receive High blood pressure-related education	Education of patients	Patients	Patient		
Kim, 2011b	Less-intensive telephone counseling	Facilitated relay of information to clinicians, Case management, Promotion of self-management	Health system; Patients	Patient + Nurse; Patient	NR	NR
	More-intensive counseling	Facilitated relay of information to clinicians, Case management, Promotion of self-management	Health system; Patients	Patient + Nurse; Patient		
Krishan, 1979	Wabasha	Team changes, Case management, Clinician education, Education of patients, Social support, Promotion of self-management	Health system; Health care providers; Patients	Patient; Physician + Nurse	No	No
	Owatoma	Education of patients, Social support	Health system; Health care providers; Patients	Patient; Physician + Nurse		
	Spring valley	Education of patients, Social support	Patients	Patient		
Chavannes, 2009	Picasso Bocholtz intervention	Team changes, Case management, Promotion of self-management, Education of patients	Health system; Patients	Patient	No	No
	Usual care	Usual care/Control	NR	NR		
Lawrence, 2008	Longitudinal adherence treatment evaluation program	Case management, Electronic patient registry, Reminders, Motivational interviewing, Clinician education, Financial incentives, Promotion of self-management	Health system; Health care providers; Patients	Patient; Care manager; Community centre	No	No
	Control	Usual care/Control	NR	NR		

Montero, 2005	Cardiac rehabilitation program	Education of patients, Team changes, Promotion of self-management, Case management	Health system; Patients	Patient + Family; Patient	No	No
	Control	Usual care/Control	NR	NR		
Mildestvedt, 2008	Intervention	Education of patients, Promotion of self-management, Team changes	Health system; Patients	Patient	No	No
	Standard rehabilitation	Usual care/Control	NR	NR		
Morisky, 1983	Educational Program Content (E1C2C3)	Motivational interviewing, Education of patients, Promotion of self-management, Usual care/Control	Patients	Patient	No	No
	Educational Program Content (C1E2C3)	Usual care/Control, Social support	Patients	Patient		
	Educational Program Content (C1C2E3)	Usual care/Control, Education of patients, Promotion of self-management	Patients	Patient		
	Educational Program Content (E1E2C3)	Motivational interviewing, Education of patients, Promotion of self-management, Usual care/Control, Social support	Patients	Patient		
	Educational Program Content (E1C2E3)	Motivational interviewing, Education of patients, Promotion of self-management, Usual care/Control	Patients	Patient		
	Educational Program Content (C1E2E3)	Education of patients, Promotion of self-management, Usual care/Control, Social support	Patients	Patient		
	Educational Program Content (E1E2E3)	Motivational interviewing, Education of patients, Promotion of self-management, Social support	Patients	Patient		
	Control (C1C2C3)	Usual care/Control	NR	NR		
Olson, 2009	Continue to receive Clinical Pharmacy Cardiac Risk Service	Team changes, Promotion of self-management, Case management, Education of patients, Electronic patient registry, Reminders, Clinician	Health system; Health care providers; Patients	Patient; Health care provider	No	No

		education				
	Usual care	Usual care/Control, Reminders	Patients	Patient		
Perk, 1989	Continued long-term cardiac rehabilitation program	Education of patients, Team changes	Health system; Patients	Patient	No	No
	Control	Usual care/Control	NR	NR		
Phillips, 2005	Copy computerized reminders	Clinician education, Reminders	Health care providers	Residents; Nurse Practitioners; Physician Assistants; Attending Physicians; Pharmacists; Nutritionists; Health Educators; Social Workers	No	No
	Individual face-to-face feedback	Clinician education, Audit and feedback	Health care providers	Residents; Nurse Practitioners; Physician Assistants; Attending Physicians; Pharmacists; Nutritionists; Health Educators; Social Workers		
	Copy computerized reminders + Individual face-to-face feedback	Clinician education, Reminders, Audit and feedback	Health care providers	Residents; Nurse Practitioners; Physician Assistants; Attending Physicians; Pharmacists; Nutritionists; Health Educators; Social Workers		
	Control	Clinician education, Usual care/Control	Health care providers	Residents; Nurse Practitioners; Physician Assistants; Attending Physicians; Pharmacists;		

				Nutritionists; Health Educators; Social Workers		
Pill, 1998	Intervention	Education of patients, Promotion of self-management, Clinician education	Health care providers; Patients	Patient; General Practitioner + Nurse	No	No
	Usual care	Education of patients	Patients	Patient		
Radziewicz, 2009	Coping and communication support intervention	Case management, Promotion of self-management	Health system; Patients	Patient	Yes	No
	Usual care	Usual care/Control	NR	NR		
Reichard, 1996	Intensified conventional treatment	Education of patients, Promotion of self-management, Case management	Health system; Patients	Patient	No	Yes
	Standard treatment	Education of patients, Promotion of self-management	Patients	Patient		
Rothschild, 2012	Receive diabetes counselling from a Promotora	Promotion of self-management, Team changes, Education of patients, Motivational interviewing	Health system; Patients	Patient	No	No
	Receive diabetes education through mailings	Education of patients	Patients	Patient		
Rowley, 2000	Intervention among high-risk overweight and diabetic people	Education of patients, Promotion of self-management	Patients	Patient + Family	No	No
	Intervention in the wider community	Education of patients, Promotion of self-management, Social support	Patients	Patient + Family		
Skinner, 2000	Learn, Share & Live education program	Education of patients	Patients	Patient	No	No
	Control	Usual care/Control	NR	NR		
Stroebel, 2000	Intervention	Clinician education, Team changes, Education of patients, Continuous QI, Case management, Promotion of self-management	Health system; Health care providers; Patients	Physician; Patient	No	No

	Usual care	Clinician education	Health care providers	Physician; Patient		
Svetkey, 2009	Physician intervention	Clinician education, Audit and feedback	Health care providers	Physician	No	No
	Patient intervention	Education of patients, Promotion of self-management, Motivational interviewing	Patients	Patient		
	Physician + patient interventions combined	Clinician education, Audit and feedback, Education of patients, Promotion of self-management, Motivational interviewing	Health care providers; Patients	Physician; Patient		
	Usual care	Education of patients	Patients	Patient		
Tamone, 2012	Intervention	Education of patients, Promotion of self-management, Case management, Reminders	Health system; Patients	Patient	No	No
	Historical cohort	Usual care/Control	NR	NR		
van Wetering, 2009	INTERdisciplinary COMmunity based COPD management program (INTERCOM) program	Team changes, Education of patients, Promotion of self-management	Health system; Patients	Patient	No	No
	Usual care	Education of patients	Patients	Patient		
Weiss, 1984	Intervention	Team changes, Promotion of self-management, Case management, Financial incentives	Health system; Patients	Patient	No	No
	Control	Usual care/Control	NR	NR		
Wisse, 2010	Intervention	Promotion of self-management	Patients	Patient	No	No
	Usual care	Usual care/Control	NR	NR		
Xian, 2010	Get With the Guidelines-Coronary Artery Disease program	Financial incentives, Audit and feedback, Promotion of self-management, Team changes, Clinician education, Reminders,	Health system; Health care providers; Patients	Hospitals teams; Healthcare provider; Patient	No	No

		Electronic patient registry, Education of patients			
	Without the Get With the Guidelines-Coronary Artery Disease program	Usual care/Control	NR	NR	

Abbreviations: NR – Not reported
a – "the consistency and quality of targeted organizational members' use of the specific innovation". [Source: Klein KJ, Sorra JS. The challenge of innovation implementation. Acad Manag Rev. 1996;21:1055–1080. doi: 10.2307/259164].
b – KT intervention adapted or changed over time
