# Supplementary material for: Sustainability of knowledge translation interventions in healthcare decision-making: a scoping review
Source: Implement Sci. 2016 Apr 21;11:55. doi: 10.1186/s13012-016-0421-7 (PMC4839064; doi:10.1186/s13012-016-0421-7)
Supplement: Supplementary file 7 — Specific intervention details from included studies. (PDF 776 kb) [file 13012_2016_421_MOESM7_ESM.pdf]

Appendix 7. Specific intervention details from included studies

Allen-Ramey, 2002	The Anthem 'Healthy Solutions for Asthma' program included provider and patient components. For providers, brochure and speaker-based educational programs were offered to HMO and POS physicians and office-based registered nurse educators managing a high volume of patients with asthma. Educational dinners were held in three locations in Ohio from April through June, 1995. Dinner meetings included discussions of the NHLBI guidelines, the proper use of asthma medications and PFMs and co-management of asthma. Provider feedback about prescribing patterns was distributed using prescription claims data during the period October 1994 through March 1995. These reports illustrated the percentage of inhaled anti-inflammatories as a proportion of all inhaled agents prescribed by an individual physician along with others within the same specialty. The Anthem Pharmacy and Therapeutics Committee recommended as a target that 60% of all inhaled preparations identified on claims reports should be anti-inflammatory medication. Prescribing patterns were compared with individual physician values from the previous time period, with those of peers for the current time period and with clinical standards. Reports were distributed on a semi-annual basis. Between 1995 and 1996, physicians with high volumes of patients with asthma (more than 250) were also offered special ongoing asthma education sessions. Additionally, individual sessions were held with an asthma specialist to review cases and other educational materials. Other provider interventions included statewide mailings of articles, practice guidelines and informational articles to all physicians under contract with Anthem as part of a quarterly clinical newsletter. The goal of the patient intervention was to educate patients about asthma triggers and to enable self-management through education, appropriate medication and avoidance of trigger factors. Overall, policy changes, an asthma fair and a compliance program were implemented. At the beginning of the study, PFMs were not typically a funded benefit at Anthem. This policy was changed to make PFMs a funded item, available free-of charge for all enrollees with asthma. A brochure and health fair-based patient education program was held in selected urban areas of Ohio. The asthma fair held in May 1995 used brochures to address various topics. Brochures emphasized a collaborative approach to management between patient and provider and aimed to improve patient knowledge. Topics included the inflammatory nature of asthma, environmental effects on asthma, avoidance of the trigger factors of asthma, proper use of beta-agonists and inhaled and oral anti-inflammatory agents, and use of PFMs. Although the patient education program was offered primarily to patients in the HMO and POS plans, information was given to any Anthem member upon request. Patients were also given the option to enroll in a compliance monitoring program. Use of inhaled anti-inflammatory agents was monitored over a 6-month period by physicians using the pharmacy claims database. Participants received a patient identification card to be signed by pharmacists, monthly reminder letters for refills, monthly newsletters on asthma management, patient diaries, PFMs and asthma management kits including pillow covers and dusters.
Ambrosio, 1983	The intervention consisted mainly of the setting up of a Hypertension Clinic, Hypertension Register, involvement of the local GP's and existing health organizations, and general public education. The changes occurring in the random sample in 5 years were expected to reflect the changes in the whole community in variables such as the treatment rate of hypertension and mean levels of blood pressure, presumably related to the intervention. The findings in the study community had to be compared with those in a reference community in which similar population surveys, but no intervention, were performed.
Bailie, 2006	The cardiovascular and renal disease prevention programme (the Renal Treatment Program (RTP)) was based on best practice guidelines, and aimed to reduce the incidence of ESRD by controlling blood pressure using angiotensin converting enzyme inhibitors (ACEi) as the mainstay of treatment (Hoy et al., 2000). The RTP was managed by a specialist physician. Follow-up and treatment were carried out at the community health centres by a specialized team of trained nurses assisted by indigenous community health workers. The team's sole focus was the specific treatment activity of the programme. It is not clear to what extent the potential withdrawal of funding had been planned for at the commencement of the RTP. However, when RTP funding came to an end in late 1999, the management approach of the program was taken on by the then recently established Tiwi Health Board (THB) which sought to integrate treatment of the RTP's participants into the routine delivery of care in the community health centres. The withdrawal of the RTP occurred within the general timeframe (late 1998–1999) of a broader health reform initiative known as the Coordinated Care Trials (CCTs) which had commenced in 1998 with the formation of the THB. Key elements of these trials, in which the health centres hosting the RTP were participants, were the implementation of 'care coordination' based on best practice guidelines for the prevention and management of a range of common chronic conditions (including renal disease) and an electronic information system designed to support these guidelines (Robinson et al., 2001, 2003). The THB expressed concern that it was not sufficiently funded to sustain the RTP at the same level as had been possible with the specialized resources previously available. Some time after the handover, the Director of the RTP drew attention to declining levels of BP control among clients of the programme. In response, the Board supported an investigation of trends in chronic disease outcomes. Findings of the evaluation of the CCT had indicated that the translation of best practice care plans into effective and sustainable improvements in health care delivery, and in turn into measurable health gains is not a straightforward process (Robinson et al., 2001, 2002). This investigation of trends in chronic disease outcomes was based on the assumption that changes in organisational structures and systems might have impacted on health benefit for clients with high levels of need within the service framework provided by the CCT. As managed by the Board, participant follow-up was done by health centre staff responsible for general health care, including the high demands of acute care to participants of all ages, with one chronic disease nurse who worked with AHWs to promote active follow-up of cases in three community

	health centres. Advice on medical management decisions and monitoring of outcomes and performance were largely left to two resident GPs. Although advice was provided by personnel of the RTP during a transition period, there was no systematic redesign of work practice protocols for the Health Board's personnel. Numerous models for designating responsibilities were tried by the Board and abandoned or modified in the months after handover. There was some resistance among staff to adoption of the specific responsibilities for the targeted programme. Prompt feedback to practitioners and participants; Opportunistic follow-up by health center staff, supported by electronic reminder system; Assignment of chronic disease care plan(s)
Baker, 2001	In an attempt to support the provision of routine care to patients with diabetes, we at the Center for Health Services Research, Henry Ford Health System (Detroit), in association with a former colleague [R.E.W], developed a Web-based Diabetes Care Management Support System (DCMSS). In designing the system, we had three key goals. First, we wanted to ensure that current clinical practice guidelines pertaining to the care of patients with diabetes were readily available to practicing clinicians.' Second, using automated data sources, we wanted to construct registries of patients with diabetes that included pertinent clinical and administrative information to support proactive, population-based interventions. Third, we wanted to provide routine reports for continual feedback in a manner that allowed performance monitoring and benchmarking comparisons. Although this system was designed to be the necessary infrastructure for subsequent care management interventions, we wondered whether the system itself had any effect on the quality of care delivered to patients with diabetes. This article describes the resulting system and provides an evaluation of its effectiveness in improving the rates of routine monitoring and screening among patients with diabetes.<br><br>The web-based-DCMSS was implemented within a large multispecialty group practice that provides care to approximately 350,000 members of a nonprofit health maintenance organization (HMO) and to approximately the same number of individuals within a variety of fee-for-service financing arrangements. Within the medical group, all patients are asked to select a primary care physician (PCP). Selections are recorded in a computerized panel management system, with patients classified as being "aligned" with their selected PCP. At the time of the evaluation, the medical group was organized into five operational regions, each of which had its own medical director and administrative staff. In total, 190 PCPs for adults (general internists and family practitioners) staffed 31 ambulatory care clinics. Within the medical group, PCPs and other providers have access to patient information through an electronic medical record that is accessible via local work stations within ambulatory care clinics and inpatient hospital units. Both inpatient and outpatient care delivered by the medical group are documented in the electronic medical record, which includes encounter history, pharmacy information, test results, and dictated notes. Initial system design and development began in early 1997. By June 1997 a prototype was developed and meetings with user groups were held to solicit feedback from key clinical and administrative leaders. The initial system was implemented in fall 1997, at which time access was granted to a small group of beta testers. Input from these users was received, and enhancements to the system were made in spring 1998. Once the enhancements were completed, the DCMSS, the first web-based care management application available within the medical group's electronic medical record, was presented to each of the local quality improvement (QI) teams at one of its regularly scheduled meetings. Team members were responsible for ensuring that PCPs and other care providers at their local sites were aware of the system and how to use it. By fall 1998 each local team had been made aware of the system and its capabilities. Throughout the study period (January 1, 1998-October 31, 1999), we compiled monthly usage statistics that described which users initiated a DCMSS session and the number of sessions they initiated.
Baker, 2011	The intent of the Health Buddy Program is to augment and improve care management using a telehealth tool that gives providers an opportunity to communicate better with patients and thus improve the information available to care managers. For the demonstration, the program intervention was implemented at the medical practices of the Wenatchee Valley Medical Center in Wenatchee, Washington, and the Bend Memorial Clinic in Bend, Oregon, in conjunction with the developers of the program, Robert Bosch Healthcare and the American Medical Group Association. The program used the Health Buddy device, a handheld device with four buttons and a large, high-resolution color screen located in a patient's home and linked via telephone with care managers. Patients using the device received daily questions tailored to their diagnoses about such things as their symptoms, vital signs, knowledge, and health behavior. Patients' responses were uploaded to a web-based computer application that risk-stratified responses for review by the care manager. The application incorporated an "exception based" approach that aimed to identify the need for care management interventions based on deteriorating vital signs and symptoms and to identify gaps in patients' behavior and knowledge. After reviewing patients' information, care managers could contact patients who appeared to be at risk for deterioration or who required intervention to ensure that they received appropriate services. As part of the demonstration protocol, the clinics and program developers worked with CMS to define the group of beneficiaries to whom the program would be offered ("intervention patients"), using a two step process. First, CMS used Medicare records to identify traditional beneficiaries who lived in one of the counties primarily served by the two clinics. Second, CMS chose patients from this group who met specified selection criteria based on their claims data. A primary goal of CMS's selection algorithm was to identify patients who had evidence of congestive heart failure, chronic obstructive pulmonary disease, or diabetes mellitus—three conditions targeted by the CMS demonstration project. The selection algorithm required that intervention patients receive most of their care at the study clinics. It identified appropriate high-risk, high-cost patients while excluding others who would not be candidates for interacting with the program (see the online Appendix). CMS assigned patients to the intervention group at two different time points, creating two different cohorts. The first cohort was identified in early 2006 and consisted of 763 beneficiaries. To expand the study

	and account for attrition due to death and other causes, CMS identified a second cohort of 1,056 beneficiaries in early 2007. Care managers at each clinic contacted patients in the intervention group and invited them to join the study. Contacts took place over a period of several months, beginning in February 2006 and February 2007 for the first and second cohorts, respectively. Beneficiaries who accepted the invitation received a Health Buddy device at no charge to them. The device was connected to their telephone line. Once the device was active, the program prompted beneficiaries and their case managers to enter or receive information about their health status regularly. Approximately 37 percent of beneficiaries in the intervention group accepted the invitation, received the device, and entered information into the device at least once ("engaging" the program). Patients who used the device at least once supplied information on an average of 300 days over the study period. This research evaluates whether the program influenced health care spending. The intervention could have affected beneficiaries' health and spending through at least two channels. First, interacting with the program could improve beneficiaries' knowledge about their conditions and how to manage their health better. Second, the technology platform could improve care coordination. That is, care managers with more and better information might be able to help beneficiaries manage their conditions and improve their health, intervening before a patient needs to visit the emergency department or be admitted to the hospital. Armed with this information, other providers might also be able to improve their practice patterns.
Bakitas, 2004	In 1998, Project ENABLE, one of four funded cancer center/hospice collaborations of the RWJF Promoting Excellence program was conceived. The major program goal was to enable patients with advanced cancer and their families to take full advantage of palliative care services from the time of diagnosis.ENABLE provided an integrated approach to the management of life-limiting cancer aimed at alleviating the symptoms of disease and treatment, enhancing clinician and patient/family communication, offering support for families, friends and other caregivers, addressing emotional and spiritual needs of dying people and providing conceptual and administrative infrastructure for achieving EOL care consistent with patients' values and preferences. Project ENABLE encompassed three functions: (1) to provide a palliative care nurse or nurse practitioner to coordinate care of patients and their families across clinical specialties and community agencies, especially emphasizing symptom and quality-of-life assessment and management; (2) to offer a workshop series for patients and families focused on living with life-limiting illness; and (3) to integrate palliative and hospice care into routine oncology care. As a demonstration project, it was evaluated by the Dartmouth Committee for the Protection of Human Subjects and found to be exempt, however, procedures for confidentiality and anonymity of data management consistent with quality management and improvement procedures were followed.<br><br>**Palliative care nurse coordination and symptom and quality-of-life assessment**<br>Experienced oncology nurses, called palliative care coordinators (PCCs), who had strong associations with the three clinical sites (two master's prepared advanced practice nurses [APNs], and one registered nurse, supervised by an APN coordinator) were recruited, trained, and participated in the development of focus groups, patient recruitment, data collection procedures, and workshop curriculum. A variety of assessments were collected at different time points: (1) The Edmonton Symptom Assessment Scale (ESAS) was collected monthly; (2) the disease-specific and palliative care versions of the Functional Activities of Cancer Therapy (FACT) was collected quarterly; and (3) the Missoula Vitas Scale was collected once when the patient was considered hospice-eligible. Three to 6 months after the patient's death, a trained interviewer, located at NCCC, conducted a follow up, after-death 68-item closed-ended survey with next-of-kin or a caregiver involved with the patient throughout their course of illness. All but two interviews were conducted by telephone; two family members preferred to answer survey questions in person. After a 4-month pilot of study procedures, site specific project presentations to oncologists and oncology practice staff, and data collection on an historical control patient group the intervention was initiated (July/August 1998). PCCs identified potential participants by the mechanisms described above and then contacted eligible patients by phone, at a clinic appointment, or in the hospital within 2 weeks of identification. On the initial contact the PCC described Project ENABLE and offered the patients the opportunity to participate. The PCC met with each patient/family in conjunction with scheduled oncology appointments and/or communicated by telephone regularly as determined by the symptom assessment/ quality of life data collection schedule as well as the unique needs of the patient. Early in the course of illness, the PCC assisted the patient and family to identify a coordinated health care team that would carry them through their entire course of illness. The team, with patient/ family as the leaders, generally included the oncologist/ oncology nurse practitioner, primary care provider, and multidisciplinary resources (spiritual, social work, home care/hospice nurses, volunteers, friends, etc.) within the patients' local community (usually through the local hospice organization). The purpose of this activity, conducted within the workshop (see below), or individually if the patient could not attend, was to facilitate continuity, coordinate home care/hospice care and offer support and symptom management and care planning advice across the illness trajectory including family bereavement.<br><br>**Patient/family palliative care workshop: development and conduct**<br>Two members of the Project ENABLE team (M.K., K.N.) coordinated the development of a four session psychoeducational workshop series for patients and families focusing on important concepts of palliative care. Workshop development is described in detail elsewhere and briefly summarized here. The philosophy and content were solicited from patient, family and provider focus groups at each of the three sites. Six focus groups, one each with patients and families (*n* = 16) and clinical providers (*n* = 30), at each of the three clinical sites were used to design the workshop content and materials. Additionally, childbirth educators at DHMC were consulted regarding the design of recruitment strategies, session

	format, and the use of interactive, multimedia materials. Specific suggestions incorporated from their childbirth class model included offering the classes to all enrollees, encouraging family participation, focusing on key information for a new, but stressful life event, and "normalization" of the illness experience and death as part of life. Finally, a cancer caregiver educational program, developed by researchers from the University of Pennsylvania School of Nursing and funded by the Cancer Control Advisory Board of the Pennsylvania Department of Health, was also reviewed. Some components of this program had been adapted and reproduced by Ortho Biotech as the Strength for Caring Program. Selected Strength for Caring program materials were donated by and used with permission of Ortho Biotech. The final content was pretested in two pilot workshops and the curriculum was modified in response to the participants' evaluations. Modifications included expanding the session duration from 11/2 to 2 hours and increasing emphasis on nutrition, financial/insurance issues, and sexuality content. The primary aim of the workshop was to empower patients and families to "take charge" of the experience of living with life-limiting cancer, with specific skill development to aid participants in navigating through a complex health care system. "Charting Your Course: A Whole Person Approach to Living With Cancer" was selected as the workshop series title. The title, logo, promotional materials, and curriculum built on the metaphor of a seafaring journey. Topics included symptom management, sense of personal control, support networks, financial issues, community resources, spiritual issues, decision making and planning, communication with clinicians, stages of grief, listening skills, complementary therapies and impact of illness on families and friends. All participants were given a canvas bag containing patient education materials on common symptoms (predominantly American Cancer Society (ACS) and NCI patient education pamphlets), a calendar/symptom journal (a component of the Strength for Caring/Ortho Biotech program), and a looseleaf binder of written resources and interactive exercises developed by the Project ENABLE staff. The looseleaf format facilitated adaptation of materials to different sites and time frames and allowed for easy updating when new materials become available. The workshop format was interactive with an educational, rather than a support group, focus. Although information was central to the curriculum, a supportive environment was created in the context of facilitated sharing. Additional design features of the workshops were clear objectives for each session shared with participants, variation in presentation style with a minimum of lecture, reliance on handouts for most information, a relaxed atmosphere with ample time for discussion and introduction of EOL issues well into the series after establishing trust. The PCC and a hospice social worker (MSW) or hospice director at each site served as facilitators and were trained in workshop conduct by observing a complete series of workshops and participating with the patients and families in each activity. Other professionals with special expertise (e.g., a nutritionist, financial aid specialist, hospice director, etc.) presented parts of the curriculum. The workshops were held in conference rooms at each of the sites and light refreshments were available.
Barrera, 2001	The MLP is a multiple-behavior–change intervention that was evaluated initially with a predominantly European American sample of 279 postmenopausal women with type 2 diabetes in Lane County, Ore. Because postmenopausal women with diabetes are at particularly high risk for heart disease, the MLP had the joint goals of reducing the risk for heart disease and improving the self-management of diabetes. The program was modeled on intervention procedures that were found to be effective in reducing heart disease risk in middle-aged men and with women in the Women's Lifestyle Heart Trial. After an intense 6-month intervention period, the MLP extended intervention activities for an additional 18 months with the goal of improving the maintenance of its initial beneficial effects. The MLP is unique among diabetes lifestyle interventions in that it provides an intense, multi-component intervention intended to improve a number of outcomes, including those with special relevance to heart disease.<br><br>**Conceptual framework**<br>The conceptual model underlying the MLP specified four content areas that corresponded to diabetes and heart health risk factors: insufficient physical activity, unhealthful diet, unmanaged stress, and lack of social support. Hour-long segments of intervention sessions were devoted to each content area. Because of the small number of smokers enrolled in the MLP (and ¡Viva Bien!), individualized assistance was provided for smoking cessation. Social cognitive theory guided behavior change in the MLP and ¡Viva Bien! Intervention sessions were action oriented, including modeling of target behaviors and practice in performing those behaviors. Sessions were primarily group occasions for exercising, reducing stress, eating healthfully, providing and receiving social support, and observing peers who were doing the same. Limited time was devoted to didactics. Women received rewards for behavior change, largely in the form of recognition from staff and fellow participants and occasionally in the form of inexpensive prizes and certificates. Within this conceptual framework, intervention activities were hypothesized to have a crucial proximal effect on self-efficacy, problem solving, and social support—general psychological assets that would aid in improving physical activity, healthful eating, stress reduction, and smoking cessation.<br><br>**Program participants**<br>The MLP's efficacy was evaluated in a clinical trial that randomly assigned 279 women to either usual care (UC) from their existing health providers (*n* = 116) or usual care plus the MLP (*n* = 163). All participants were recruited from the primary care practices where participants received their medical care for diabetes. Fifty-one percent of the confirmed eligible primary care patients who were contacted began the program. At baseline, participants had an average BMI exceeding 35 kg/m2 and a mean age of 61 years (range 39–74), and 94% were non-Hispanic whites.

	**Intervention components**<br>Participants in the UC condition continued with their regular medical care and did not receive any of the MLP intervention activities. UC was directed at diabetes control, management of diabetes complications, and monitoring of other health factors such as cholesterol and hypertension. Participants assigned to the MLP condition began with a 2½-day non-residential retreat, during which the women were introduced to staff members and to each other, heard inspirational testimonials, and received an overview of all program components. The intensity of the retreat was designed to signal a distinct beginning to lifestyle change, elevate belief in the possibility of change, and build solidarity.<br>The retreat was followed by weekly 4-hour meetings throughout a 6-month period. Weekly meetings devoted 1 hour to each of the four components: physical activity, stress management, a Mediterranean Diet–style potluck, and support groups. An exercise physiologist, stress management leader, and support group leader were present at every program meeting. Sessions were conducted at a research site that had facilities for preparing foods, a large floor for conducting exercise and stress-management activities, and small rooms for support group meetings. Motivational techniques such as contests, self-monitoring, and group and individual rewards were used to encourage attendance. Small incentives (candles, refrigerator magnets, and pins) were given to women for recording adherence to program components. In addition, $100 cash prizes were given for excellent attendance. Of all the motivational techniques used, we believe the most effective was telephone calls from support group leaders and other group members when participants missed meetings.<br><br>*Dietary practices:* A registered dietitian taught participants about the Mediterranean-style alpha-linolenic acid–rich diet. The diet recommended more whole-grain bread; more root vegetables, green vegetables, and legumes; more fish and poultry; less red meat; daily fruit; and avoidance of butter and cream, which was to be replaced by olive and canola oil products. For the weekly meetings, participants brought dishes to share in potluck style. Meals were occasions to receive feedback on food preparation and portion control. Participants were asked intermittently to complete and bring to the weekly meetings a simple self-monitoring log of their adherence to components of the Mediterranean diet.<br><br>*Physical activity:* Participants were advised to build up to 1 hour of moderate aerobic activity per day, at least 3 days per week. They were encouraged to engage in a variety of physical activities, but walking was recommended for most. At the retreat and weekly meetings, participants could choose between 1-hour aerobic sessions led by an exercise physiologist or an outdoor walk led by trained exercise assistants. Resistance train exercise bands and dumbbell weights at the retreat and weekly meetings and were given a set to take home. Details of the physical activity recommendations were described and illustrated in the program guidebook and on a take-home exercise video.<br><br>*Stress management:* Participants were instructed in yoga, progressive deep relaxation, meditation, and directed or receptive imagery. Stress-management leaders were certified yoga instructors. Participants were asked to practice the techniques for at least 1 hour per day and were provided videotapes for home use.<br><br>*Support groups:* One professional with at least master's degree–level training in counseling psychology and one peer leader led each support group. Professional and peer leaders received extensive training in the supportive-expressive group therapy model used with the chronically and terminally ill. A clinical psychologist who was a research project staff member provided weekly supervision to group leaders.<br><br>*Maintenance procedures: 6–24 months.* After the first 6 months of the intervention, MLP participants were further randomized either to continue with group meetings (39 meetings spread during the next 18 months led by lay leaders) or to a personalized support-enhancement condition, which met just four times. The personalized-support condition used an interactive computer program designed to support improvements in the lifestyle behaviors acquired during the first 6 months. However, analyses of follow-up data revealed no meaningful differences between the two maintenance conditions, so they were combined and compared to the UC condition in all statistical analyses.
Behnke, 2003	Patients of the training group performed a 10-day hospital-based supervised training, while those of the control group did not (figure 1A). This was followed by the home-based training (figure 1B), whereby patients of the training group were instructed to practice 15 min of walking at home 3 times per day, in each session achieving 125% of the best 6-minute treadmill distance at discharge. Individual training modalities were established during a doctor's visit in the patient's home. The control group did not receive specific instructions for exercise, neither during the hospital stay nor at home. We aimed at integrating target points for walking that were helpful for the patient's daily needs (bakery, newsstand, etc.), expecting to improve its implementation into daily activities and to enhance motivation. During the first 3 months, patients of the training group were visited every 2 weeks by a doctor to check their health status and eventually adjust the walking distance. Afterwards, the contact was maintained by monthly phone calls. The only advice given was to perform regular exercise, as usual in clinical practice. Patients of the control group had no visits at home, but also monthly phone calls. The home-based programme was performed over 18 months, extending the initial 6-month trial, where only exercise performance and quality of life had been evaluated. Patients visited the laboratory for assessments of lung function, exercise capacity, quality of life (QOL) and overall dyspnea at 6, 12 and 18 months, whereby the 6-month data served as baseline for those of 12 and 18 months. To facilitate comparisons, we also show the data obtained at the start (day 0) and the end (day 10) of the hospital-based programme. As CRQ was not assessed on day 10, data from day

	0 and month 3 are shown. Both groups were instructed to document medication and, in case of the training group, activity in diaries; these were collected monthly by post, and fresh forms were sent back.<br><br>**Diaries**<br>All patients used forms for the documentation of medication, in particular β2-agonists as needed, doctoral visits, exacerbations and hospital admissions. Patients of the training group had additional forms to record walking distance and the time spent on this. Exacerbations were defined in terms of symptoms. We distinguished between „major“(dyspnoea, sputum purulence, sputum amount) and „minor“(wheeze, sore throat, cough, symptoms of a common cold) respiratory symptoms. An exacerbation related hospital admission was assumed if two major symptoms or one major and one minor symptom had increased within the two days before admission. At no time, however, we did interfere with the diagnostic and therapeutic decisions made by the GPs who took care for the patient.<br><br>**Assessments during Hospital Visits**<br>Lung function and exercise performance as well as quality of life scores were assessed in the same way as described previously. Parameters comprised total lung capacity (TLC), intrathoracic gas volume (ITGV), residual volume (RV), FEV1, vital capacity (VC), and diffusing capacity for carbon monoxide (TLCO). The baseline/transitional dyspnoea index (BDI/TDI) was used to quantify overall dyspnea. Patients also rated their sensation of dyspnea at rest on a modified Borg Scale. Quality of life was assessed by the Chronic Respiratory Disease Questionnaire (CRQ) using the dimensions „dyspnoea“, „fatigue“, „emotion“, and „mastery“. The scores of the four dimensions were added to obtain an overall score. All interviews were done by the same investigator and patients were aware of their previous answers, as recommended. Exercise tests were performed as self-paced treadmill tests [15] on a horizontal treadmill equipped with a speed control. To assess the 6- minute treadmill distance (6MTD), patients were instructed to walk as far as possible in 6 min; no further encouragement was given.
Belardinelli, 2012	**Exercise training protocol.**<br>Exercise sessions were performed under the supervision of a cardiologist and an exercise therapist, as previously described. The training program consisted of 3 sessions per week at the hospital for 2 months, then 2 supervised sessions the rest of the year. Every 6 months, patients exercised at the hospital, and then they returned to a coronary club, where they exercised the rest of the year. The coronary club is a nonprofit association of patients with cardiac disease promoting ET and healthy lifestyle changes. The intensity was chosen at 60% of peak VO2 for the first 2 months, and thereafter at 70% of peak VO2 for the following months until the end of the study. After each annual CPET evaluation, we calculated heart rate from peak VO2 to maintain training intensity at 70% of peak VO2. Trained patients were encouraged to exercise without supervision at home at least a third time, perform supervised sessions. Each session lasted approximately 1 h, beginning with a warm-up phase of calisthenics and stretching for 15 to 20 min, followed by 40 min of aerobic activity on a cycle ergometer, a treadmill, or both. Blood pressure was measured before the warm-up on a sitting position, at the middle of aerobic exercise, and at the fifth minute of recovery. Heart rate was monitored throughout the session. A written report of each session's activities and the patient's heart rate and blood pressure was completed by the supervisor. Patients also received counseling on smoking cessation, stress reduction, and diet. Before randomization, educational sessions were organized at the hospital with groups of patients to explain the objective of the study; the benefits of a healthy nutrition, physical activity, and smoking cessation; and the importance of medications prescribed to CHF patients. A printed summary of the session with examples of diet, exercise methodology, and calisthenics was given to all participants. Educational sessions were performed each year thereafter to participants in both groups until the end of the study.<br><br>**Usual care.**<br>Patients in the NT group were not provided with a formal ET program. They were instructed to continue their usual home daily physical activities, avoiding ET in a supervised environment. All patients were recommended to take the usual medications and received counseling on nutrition, stress reduction, smoking cessation, and physical activity. They also were instructed to avoid high intensity exercise. They were free to perform aerobic activities such as walking, cycling (home or outside), and swimming, avoiding a duration of longer than 30 min. We did not specify the intensity of exercise in the NT group, because of the potential of bias. However, we advised them to walk and to continue to perform usual physical activities. They were seen at the cardiology clinic every 3 months and were asked to call the supervisor every time they had a question or problem.<br><br>**Cardiopulmonary exercise testing.**<br>After a familiarization test, all patients performed CPET at the Lancisi Hospital before the protocol was started and every 12 months thereafter until the end of the study. Briefly, work rate was increased 1 or 2 W every 5 s in a ramp fashion until volitional fatigue or symptoms such as dyspnea, chest discomfort, or dizziness emerged. Patients pedaled on an electronically braked cycle ergometer (Ergometrics 800, Sensormedics, Yorba Linda, California) at a constant rate of 60 rpm. During exercise, patients breathed room air through a

	Rudolph mask and ventilation and gas exchange were measured breath by breath on a metabolic cart (Cosmed Quark PFT, Rome, Italy). Peak VO2 was the average oxygen uptake over the last 15 s of exercise. Maximum VO2 automatically was provided by the system on the basis of age, height, weight, gender, and race. Percentage of peak VO2 versus maximum VO2 then was calculated for each comparison. Ventilation relative to carbon dioxide output (VE/VCO2) slope was calculated as the slope between ventilation (y-axis) and VCO2 (x-axis) by linear regression analysis. Gas exchange ratio was the ratio between VCO2 and VO2.<br><br>**Echocardiography.**<br>M-mode and 2-dimensional echocardiographic studies were performed on study entry and every 12 months in all patients according to the recommendations of the American Society of Echocardiography. We used an ultrasound system with a phased-array 2.5-MHz transducer (Megas, ESAOTE, Rome, Italy). Details have been described previously.<br><br>**Quality of life.** QOL was assessed at baseline and every 12 months in both groups, as previously described. We used the Minnesota Living with Heart Failure Questionnaire, which is a patient self-assessment measure and consists of 21 items focused on patient perceptions concerning the effects of CHF on their physical, psychological, and socioeconomic lives.
Berg, 2007	In November 2000, registered nurses began calling identified members for program enrollment. For those who agreed to enroll, McKesson Health Solutions customized a self-management intervention plan that included risk stratification; formal scheduled nurse education sessions; 24-hour access to a nurse counseling and symptom advice telephone line; printed action plans, workbooks, and individualized assessment letters; medication compliance and vaccination reminders; and physician alerts about signs and symptoms of decompensation as well as notification to physicians of gaps between patient-reported practice and guideline recommendations. Risk stratification was determined from direct patient assessment of medical service utilization, self-management practices, medical history, medical management, and psychosocial factors. The tool employed Boolean logic and sorted patients into 3 categories, which determined the frequency of scheduled calls over the course of the year. Physician communications took place through letters, faxes, and phone calls. Two-way communication was encouraged from the physicians back to the DM nurse on recommendations for further counseling topics or clarification of patient-reported information. In addition, the DM nurse communicated with the health plans' case managers for provision and coordination of plan benefit issues such as durable medical equipment procurement, mental health visit coordination, transportation difficulties, or financial barriers to adhering to physician recommendations. Communications to physicians and case managers occurred regularly, usually after each scheduled patient call. The telephonic interventions took place primarily in the participants' residences; some participants did not have a telephone at home, in which case a convenient community-based alternative was employed. The American Diabetes Association guidelines, which are updated and published annually, form the basis of the intervention. Once patients were enrolled in the program, nurses conducted a telephonic assessment at intake, 6, 12, 18, and 24 months to assess each participant's knowledge, behavior, and health status related to his or her diabetes. The improvement in patients' knowledge, behavior, and health status was expected to lead to changes in their utilization of medical services. Initial assessments ranged from May 2000 through March 2002. The control group members received usual care from their providers. It is relevant to note that all members in the control and intervention groups were enrolled in a managed care plan that provided medical management services (ie, case management, provider networks).
Bocchi, 2008	This disease program management protocol was designed to have the following characteristics: inclusion of outpatients; intervention through education for patients and caregivers; medication management with optimized therapy based on guidelines and remote monitoring; delivery personnel with nurses, cardiologists, pharmacists, social workers, dietitians, dentists, and psychologists; face-to face individual/group communication; and telephone in-person communication. The intensity/complexity was long-term follow-up with repetitive education at 6-month intervals. The environment was hospital outpatient; the outcomes measured were clinical, quality of life, and adherence. A daily (except on weekends) telephone number was provided to patients for emergencies or questions about HF management.<br><br>*Interviewers and Education Classes*<br>Interviewers<br>• Duration: 30 min with any multidisciplinary component<br>• Personnel: nurses, pharmacists, social workers, dietitians, psychologists<br>• Design: face-to-face including all randomized intervention patients<br>• Schedule: early after randomization<br>Education classes<br>• Duration: 60 min

	• Personnel: multidisciplinary (nurse, pharmacist, social worker, dietitian, dentist, psychologist)<br>• Design: group class including all randomized intervention patients<br>• Schedule: early after randomization (four times) and during the follow-up<br>• Intervals: 1 week between the 1st and 2nd; 1 month between the 2nd, 3rd (1st reinforcement), and 4th (2nd reinforcement); and at 6-month intervals<br>Content of interviewers and educations classes<br>• Basic principles related to heart failure (HF)<br>• Causes and physiopathology of HF; causes of volume overload<br>• Rationale for pharmaceutical and no pharmaceutical therapy<br>• Importance of drug adherence, mechanisms of drug action<br>• Role of sodium, fluid restriction, and diet (unless otherwise specified by the patient's physician)<br>• Rationale for self-care behaviors, daily weight monitoring<br>• Avoidance of alcohol intake, cigarette smoking, and antiinflammatory drugs<br>• Instruction about exercise, work, and daily physical activities<br>• Signs of the onset of HF exacerbation<br>• Appropriate reaction to signs and symptoms (especially for syncope, tachycardia, hemoptyses, ascites, edema, fever, worsened dyspnea, chest pain,<br>• paroxysmal nocturnal dyspnea, nocturnal cough, new orthopnea, oliguria, anorexia, abdominal pain)<br>• Instruction about what to do if symptoms worsen or with a new symptom<br>• Potential drugs that can worsen HF (estrogen, corticosteroids, calcium antagonists, antiarrhythmic agents, etc)<br><br>*Monitoring*<br>• Telephone calls<br>• Initiation: after the 2nd group education class<br>• Intervals: scheduled at 14-day frequency (more or less according nurse's decision about necessity)<br>• Personnel: nurse trained in HF management<br>• Content: predetermined questionnaire<br>• Objective: to reinforce the education<br>• Monitor compliance/adherence, signs/symptoms of worsening HF, self-control mechanisms<br>• Adjustment of diuretic dosage by nurse if necessary<br>• Recommend unscheduled medical or emergency visit if worsening HF or noncompliance
Chin, 2007	**The Standard HDC**<br>We have previously described the HDC. In brief, each participating HC formed a HDC team that met regularly with the support of senior administrative leadership. Each center also created a registry of patients with diabetes to help track clinical care. A Model for Improvement developed by Associates in Learning called the Plan-Do-Study-Act cycle was introduced into each HC.This model adapts elements of continuous quality improvement into a process designed to improve the quality of care at an accelerated pace. The program is grounded in the MacColl Chronic Care Model, which aims to create practical, supportive interactions between an informed, activated patient and a proactive, pre- pared clinical team. Specific targets for QI are patient self-management, delivery system redesign, decision support, clinical information systems, leadership and health system organization, and community outreach. The Institute for Healthcare Improvement provided the centers initial instruction at a national learning session, and then regional cluster coordinators and Institute staff assisted through telephone conference calls, a computer listserv, feed- back on required monthly progress reports, patient registry and information systems support, and 3 regional meetings. At the regional and national learning sessions, team members and administrators from HCs met to learn QI techniques and share lessons among themselves.<br><br>**Improving Diabetes Care Collaboratively in the Community Project**<br>The 34 centers were then randomized into a standard- intensity arm and a high-intensity arm, stratifying by region and whether the HC had participated in the first or second Diabetes

	Collaborative.<br><br>*Standard-Intensity Arm*<br>The centers in the standard-intensity arm received continued interaction and support from the BPHC. This consisted of quarterly progress reports from the QI teams and senior leader-ship, conference calls with other centers and cluster coordinators, and a yearly in-person meeting with the other HCs.<br><br>*High-Intensity Arm*<br>The high-intensity arm included additional support in organizational change techniques and chronic care management, as well as interventions designed to facilitate behavioral change in patients to get them more actively involved in their care. Specifically, the high-intensity intervention consisted of the standard-intensity activities plus:<br><br>1. Four biannual 1.5-day learning sessions. The learning sessions covered QI topics, clinical subjects, and planning time for the center teams. The core elements were HC-led workshops in which HCs shared model programs, interventions, and lessons learned.<br>2. Training in patient-provider communication and behavioral change techniques. One diabetes QI team member from each HC attended a week-long train-the-trainer course, "Clinician- Patient Communication to Enhance Health Outcomes for Patients with Diabetes" at the Bayer Institute for Health Care Communication (now known as the Institute for Healthcare Communication), and then returned to his or her home HC to train its providers in a sequence of 4 two-hour or 8 one-hour learning sessions to be conducted over 4 months. These learning sessions were adapted from 2 standard Bayer Institute workshops, "Clinician Patient Communication" and "Choices and Changes" with emphasis on educating and facilitating behavior change in patients with diabetes using a Stages of Change model and principles of motivational interviewing.<br>3. Patient empowerment video, brochure, and processes of care card. We created English and Spanish versions of a 14-minute video designed to empower patients to play a more active role in their care and ask for 6 key processes of diabetes care (hemoglobin Ale test, cholesterol test, blood pressure measurement, dilated eye examination, foot examination, urine microalbumin test). The written materials and video were distributed to the centers to give and show to their patients.<br>4. Monthly conference calls. We held 16 one-hour conference calls with HC representatives to discuss issues important to the HCs, receive input and feedback for the different components of the intervention, and update the HCs on the project.
Chen, 2010	**Description of P4P Program of Study Group**<br>The P4P program financially incentivize participating physicians in Hawaii (additional 1.5– 7.5% of their base professional fees) to perform quality care processes. Only certain specialties, which were determined by the health plan, qualified for awards based on their quality scores. In addition, providers were only eligible for an award if they saw a minimum of five patients enrolled in the PPO setting who met the quality indicator denominator criteria. In 2001, an additional bonus of up to US$3,000 was added for practitioners who improved their quality scores over time. General characteristics of the P4P program have been described previously in detail.<br><br>The comparison group consisted of providers enrolled in a large commercial PPO health plan not located in Hawaii and without any performance based incentive program; these providers were enrolled in the health plan for at least 2 years during 2004 through 2007. In addition, the control group consisted of the same specialties as the study group and saw at least five patients who met denominator eligibility criteria. In other words, the control group would have been qualified to receive incentives for the P4P program had they been practicing in Hawaii. Of note, to calculate quality scores, 2 or 3 years of administrative data were needed. For example, although we had data from 2003 through 2007 for the comparison group, we can only calculate quality scores on years 200 through 2007 (4 years) for most indicators.
Cheng, 2012	Taiwan implemented a single-payer National Health Insurance (NHI) program in 1995; in 2008, approximately 98% of the hospitals nationwide were contracted by NHI, and more than 99% of all Taiwanese residents were covered under NHI. Diabetes mellitus was the fifth leading cause of death in Taiwan and accounted for 3% of the total expenditure of the NHI program in 2008. A previous report has suggested that patients with diabetes in Taiwan did not receive adequate care, especially recommended follow-up care. To enhance the medical management of diabetes, the NHI's P4P program for diabetes provides financial incentives to physicians based on the following criteria: whether patients receive follow up visits (including enhanced selfcare education), the provision of diabetes-specific examinations or tests and whether health care providers achieve a set of predetermined treatment goals. This study used a natural experimental design coupled with PSM to examine the longterm effects of a P4P program for diabetes care on health care utilization and expenses.<br><br>**Data Source**<br>This study used a natural experimental design with population-based longitudinal data to examine the long-term effects of a P4P program. The analysis was based on a 6-year panel

	of claims data for health care utilization from 2004 to 2009 in Taiwan. Using nationwide NHI claims data, we identified patients diagnosed with type 2 diabetes [International Classification of Diseases, 9th Revision—Clinical Modification, ICD-9-CM, Codes 250.xx (excluding 250.x1 or 250.x3)], who had at least 3 diabetes-related physician visits or at least 1 diabetes-related hospitalization for each year during the period from 2004 to 2009.<br><br>**The P4P Program**<br>Since 2001, the Bureau of the NHI has implemented a P4P program for diabetes care. Physicians who specialize in metabolic disorders or endocrinology or physicians who underwent a training program for diabetes shared care can voluntarily apply to participate in the P4P program. Participating physicians can then recruit individual patients into the program. The incentive payment is incorporated in the fees for the physician visit. In 2005, the P4P program paid participating physicians higher physician fees of New Taiwan (NT) $450 (in 2005, NT $33 =US $1) for comprehensive visits, which was included in 3 types of case management fees, that is, initial enrollment visits (NT $ 1845), comprehensive followup visits (NT $ 875), and an annual evaluation visit (NT $ 2245). Specific service items and full documents required for each of the visits included nutritional education, physical examinations, and laboratory tests. In addition to these case management fees, some of the examinations or test orders issued during each visit could be claimed for reimbursement by regular fee-for-services payment schemes.
Coleman, 2001	The group-visit intervention is discussed in greater detail elsewhere. This study builds on the pilot study, which was conducted in highly selected physicians' practices (the innovators of the primary care group-visit concept), based in a single ambulatory care facility. The present study selected physician practices that were more generalizable to the overall care delivery system in an attempt to translate efficacy into effectiveness. In brief, group visits are a new model of care that recognize the discordance between the expanding needs of older adult patients with chronic illness and the acute care orientation of the typical 15-minute office visit. The main goal of the group visit was to facilitate self-management of chronic illness through enhanced education, encouragement of self-care, peer and professional support, and attention to the psychosocial aspects of living with chronic illnesses. Each intervention physician practice team had one cohort of group-visit patients. The primary care physician, nurse, and clinical pharmacist constituted the core delivery team and were relieved of their other clinical responsibilities to attend each group visit. Additional ancillary providers, including a dietitian, social worker, and physical therapist, attended periodically. The group visits were held during regular clinic hours in the clinic conference room, and the patients took turns bringing refreshments. The clinic receptionist handled all of the scheduling, and the visit was subject to the usual outpatient visit co-pay. Group visits were held monthly with an average attendance of 8 to 12 participants per group. Caregivers and spouses were invited to attend. These visits had a standard format. The visit began with a brief warm-up and socialization period that was followed by a presentation on a specific health topic. Initially, the topics were the same for all groups and included such areas as normal aging, medication management, exercise, nutrition, and advanced directives. Subsequent topics were chosen based on group consensus. The next 25 minutes was devoted to health-promotion activities and included blood-pressure assessment, administration of such immunizations as influenza and pneumococcal vaccines, and medication refills. The group then reconvened for a brief question-and answer period on the topic that was presented. During this time, the next session and its health topic presentation was planned.
Corkery, 1997	Data collection instruments included a demographic data sheet, knowledge test, and patient self-report of behaviors rating scale. All tools were developed for this study and were available and administered in Spanish as necessary. The instruments were developed based on current literature and incorporated concepts considered critical to diabetes education. As such, they have high face validity. Further, experts in the field of diabetes education (diabetes nurse educators, physician endocrinologists) critiqued the instruments and agreed on the content domains. Thus, the instruments have high content validity. Content, reading level, and questionnaire structure were piloted before the study. Demographic data including age, country of origin, language spoken, educational level, literacy level, employment status, household composition, and patient-rated family support with their diabetes self-care regimen (no support, some support, good support) were obtained by the CHW at baseline. The knowledge test consisted of nine true-false responses and was given before and after the completion of the diabetes education program. A true-false format was used and was most reliable, since tests were administered orally because of high levels of illiteracy in this population. The self-report of behaviors rating scale was also administered orally and consisted of 10 questions related to performance of diabetes self-care practices. The patients' self-rated responses were based on a six-point Likert scale. The diabetes knowledge test and self-report of diabetes self-care practices were administered by the nurse at baseline and were re-administered upon completion of the diabetes education program. The mean time to complete diabetes education was 3.4 months (range 0.9-5.4). Self-care practices were again assessed at a follow-up in conjunction with a scheduled medical appointment, a mean of 7.7 months (range 6-16.2) post-study enrollment.<br><br>**Diabetes education**<br>The diabetes education program was conducted by the CDEs on an individualized, one-to-one basis in accordance with the American Diabetes Association (ADA) Standards for Diabetes Patient Education Guidelines and focused on attainment of self-care skills and behavior change strategies. Patients completed the education program when the ADA standards were met and when individualized learning objectives were completed. Thus, the time to complete the education program varied from patient to patient based on their

	individualized learning needs.<br><br>**CHW intervention**<br>The CHW was a bicultural, bilingual Hispanic-American of Puerto Rican heritage who lived in the East Harlem community and who had previously volunteered in a diabetes clinic. After collection of initial baseline demographic data, patients were randomized either to receive or not to receive CHW intervention. The CHW had no further contact with those patients assigned to the non-CHW intervention group. During the study period, the CHW acted as a liaison between the patients, their families, and health care providers for the CHW intervention group. The CHW attended clinic sessions with assigned patients. She served as Spanish interpreter, reinforced self-care instructions, reminded patients of upcoming appointments, and rescheduled missed appointments. For the non-CHW intervention group, encounters took place only between the nurse and the patient and, in some cases, the family member.<br><br>**Family member participation**<br>Patients were defined as having family participation in the education program if a family member attended most of the education sessions.
Daniel, 1999	**Interventions for behavioural change**<br>Activities included: aerobics and gentle exercise classes, walking group, `100 Mile Club', health events, cooking demonstrations, smoking cessation group, supermarket and restaurant tours, forums on diabetes, Diabetic Support Group and targeted education. These actions were to facilitate the development of skills for healthful living. Measures of programme contact with individual residents are not available, though summary participation scores were recorded. A media campaign involved educational articles in a local newspaper, and newsletters with tips for exercise, diet and weight loss and briefs on project events. Stories were carried by municipal newspapers and television and radio stations. Connections were established with and resources obtained from diabetes and heart and stroke disease organisations. Media were used to educate and empower community action, to promote supportive environments. As data were collected and preliminary analyses undertaken, public meetings were held to dis- cuss aggregate community level results. Attendees received or had updated booklets by which to monitor change in blood values, weight, fatness and fat distribution measures. Meetings were held 2 months following each of the three waves of data collection.<br><br>**Environmental support for behavioural interventions**<br>The Band Council sanctioned a monthly distribution<br>of flyers about diabetes, weight loss, proper diet and the importance of physical activity. Local businesses allowed posting and distributing information from their premises. Use of community facilities was granted for screening initiatives and activities. A notable action was the hiring by the Band Council, mid-way through the project, of a recreation coordinator to promote physical activity and create acceptable opportunities for exercise. The Medical Services Branch granted staff support for some activities. An Advisory Committee of eight residents met regularly with the project team, providing feedback on the intervention process.
Del Sindaco, 2007	**Disease management programme group**<br>The programme was based on a hybrid model, combining hospital clinic-based and home-based care. In each of the two participating teams, the members were a cardiologist experienced in geriatrics, two to four specialized nurses and the patient's primary care physician. According to the European Society of Cardiology guidelines, the components of the programme were: discharge planning, continuing education, therapy optimisation, improved communication with healthcare providers, early attention to signs and symptoms, and flexible diuretic regimen. A written list of recommendations, a weight chart, a contact number available 6 h/day, and an educational booklet were provided only to these patients. They were encouraged to present their discharge/visit summary and weight chart at all visits. Follow-up was based on hospital clinic visits, periodical nurse's phone calls and home or office primary physician visits. The cardiologists were the case managers, designed to document the treatment plan. Hospital visits occurred in the heart failure clinics within 7 to 14 days of discharge and at 1, 3 and each 6 months thereafter. At each visit, patients received reinforcement of education and optimization of therapy. Nurses made follow-up phone calls to patients, received the patients' calls and contacted patients when they did not present to scheduled visits. They could not modify therapy; however, they could recommend that the patient consults the cardiologist or primary care physician when the patient's status deteriorated abruptly or the patient experienced a significant problem requiring prompt attention. The nurses also played a pivotal role in education programme and coordination of patient management. The primary care physicians were asked to assess adherence to treatment, evaluate possible adverse drug reactions and identify and possibly treat at home signs of worsening clinical condition, concurrent infections or comorbidities and potential precipitating factors, as well as dietary regimen. They managed all problems not related to heart failure, received regular written updates from the hospital team and were notified of abnormal laboratory and clinical values.

	**Usual care**<br>After discharge, patients assigned to UC received all treatments and services ordered by their primary care physician and/or personal cardiologist. The baseline clinical evaluation and therapeutic plan were documented in the patient's chart. In this group, vital status and events were recorded by means of phone calls performed every 6 months.
Dennison, 2007	In a 5-year randomized clinical trial with 309 hypertensive urban African American men aged 21 to 54 years, the effectiveness of a more intensive educational/ behavioral/pharmacologic intervention provided by a nurse practitioner/community health worker/physician team was compared to less intensive information and referral intervention. Changes in behavioral factors, health care utilization, blood pressure (BP) control, left ventricular hypertrophy (LVH), and renal insufficiency were evaluated.<br><br>**More intensive intervention**<br>• Education<br>• Individualized NP/CHW/MD Team Care<br>• NP visits as needed at least every 2 to 3 months<br>• ARB Losartan 50 to100 mg (free)<br>• ± Hydrochlorothiazide (HCTZ) _12.5 to 25 mg (free)<br>• Additional HTN meds as needed<br>• Home visits annually<br>• Social support engaged and mobilized by CHW<br>• Transportation<br>• Social services referrals as needed<br>• Employment guidance<br>• Birthday and holiday cards, encouragement postcards<br><br>**Less intensive intervention**<br>• Education<br>• Provided list of community HTN care sources
Desouza, 2010	A shared medical appointment (SMA) is a model of care which involves multiple patients being seen as a group and is distinct from educational group sessions. It provides a unique way of delivering patient care in a group setting. Group clinics have been shown to improve outcomes in the setting of chronic diseases [4]. However, most of these studies were short-term and did not have controls. There is no data regarding the effectiveness of group medical clinics in maintaining diabetes control over longer periods when compared to a control of usual care. Our hypothesis was that group medical clinics are as effective or better than repeated visits to a primary care provider for the maintenance of long-term diabetes control. Hence, we studied the effectiveness of group medical clinics to maintain diabetes control in complex diabetes patients over 2 years when compared to usual primary care.<br><br>Patients with difficult to control or uncontrolled diabetes are referred to the diabetes clinic at the Omaha VA Medical Center. Their diabetes is controlled at the clinic and patients are then given the option of either going back to their primary care provider (PCP) or the group diabetes clinic (GDC) at the time of discharge. A retrospective review of the electronic medical records of patients who were discharged from the diabetes clinic between 2004 and 2008 was conducted. The total number of patients discharged from the diabetes clinic during this period were 68. Patients were included in the study if their A1c at discharge was <7 and had subsequent outcome data available every 6 months for a period of 2 years. The criteria were met by 56 patients. Twenty-nine patients had enrolled in the group diabetes clinic (GDC cohort) and 27 had resumed care with their primary care provider (PCP cohort). Demographic data such as gender, age, race/ethnicity, and type of diabetes were obtained. Primary outcomes included hemoglobin A1c (A1c), low-density lipoprotein (LDL), and blood pressure (BP). The outcomes were collected at the last diabetes clinic visit (baseline), the first PCP or GDC visit and then every 6 months for a total of 24 months. The GDC consists of 8–10 patients who attend 90-min group sessions every 3 months where their blood glucose, A1c, LDL, SBP, DBP, and weight are reviewed with the other group members and a physician. Medications are adjusted and lifestyle changes reinforced. A diabetes educator/dietician provides patient-centered education at every clinic. The mean number of visits to the PCP were 2/year vs. 4/year for the group clinic. The study was approved by the subcommittee for human studies which is the IRB for the VA.

Erfurt, 1990	At each site an external, hospital based health promotion program provider located near the plant was identified to provide smoking cessation and weight loss programs. These programs were carried out at the plant, during break periods, or before and after work hours on the employees' own time. The cost of the full-group programs ranged from $50 to $100 per participant. The plant paid two thirds of this cost and the employee paid one third. Treatment for high blood pressure was provided by each employee's own physician. Each of the four sites had an internal "wellness committee," comprised of plant representatives from management, labor, and the plant medical department, to help plan, coordinate, and carry out in-plant activities.<br><br>At Site 1, the "control site,'' the only activities carried out by the study were the initial screening in 1985 and the rescreening of a random sample of initially screened employees in 1988. This is not a "no intervention" site, but a "business as usual" site. The plant conducted some wellness-related activities independently of the study. Specifically, in 1986 Site 1 set up a physical fitness center with a full-time professional attendant on the premises during most of the plant's working hours. Also, as noted above, there was a nearby hospital-based wellness program identified for each of the four study sites, in order to insure that differences in program participation would not be attributable to availability of programs within the nearby community. Health improvement classes had been offered occasionally at all four participating plants prior to the study's inception. This was a normal activity for these plants. The study design for Site 1 did nothing to encourage or discourage classes during the study period. Finally, as part of a program that had been initiated several years prior to the study, employees with high blood pressure were encouraged to come to the plant medical department for monitoring.<br><br>At Site 2, the ''health education" site, the intervention design proceeds from an underlying assumption that people will make health behavior changes if they have proper information about their current health status, and ways to learn how to make appropriate behavior changes. The program interventions implemented at Site 2 resemble the most common model of wellness programming used in workplace health promotion programs: health risk appraisal followed by media promotion and health improvement classes. A part-time health educator was assigned to work with the wellness committee at Site 2, to assist with the media promotion of the program and to help coordinate the health improvement classes with the external health promotion provider. Classes were offered about twice a year. In addition, as at Site 1, monitoring for employees with high blood pressure was offered through the plant medical department throughout the study period.<br><br>The underlying assumption of the model tested at Site 3 was that behavior change requires not only information, but support, encouragement, and assistance in problem Solving, along with a broad range of types of services or interventions. The model allows individualizing of health improvement approaches, as the wellness counselors worked with individual employees. At Site 3, health education interventions identical to those offered at Site 2 were combined with outreach and follow-up counseling procedures modeled after those used in previous studies of hypertension control. All employees with one or more of the three targeted cardiovascular risk factors — high blood pressure, 20 percent overweight or more, and/or smoking cigarettes — were included in the follow-up caseload at Site 3. Clients were contacted approximately once every six months, by two part-time wellness counselors who had an office in the plant's medical department. Client records, however, were not merged with medical department records, but were maintained separately and were kept confidential from all company personnel. Outreach included mailings and phone calls to the employee's home, and memos and phone calls to his/her work station. During follow-up, the employees were counseled about their current risk status and were assisted in exploring how they might change their risks. More detailed information about this intervention is reported elsewhere."<br><br>The Site 3 model offered a "menu approach" to wellness interventions, an option that was not possible within the model used at Site 2, because it required the presence of wellness counselors. There were four options on the ''menu.'':<br>1. Guided self-help, in which the wellness counselor helps initiate the intervention, introduces self-help materials to the client, and then guides the client through the process via a series of in-person or telephone follow-up contacts;<br>2. One-to-one formal consultation, in which an instructor from the external provider of wellness classes/services works individually with the employee in a series of formal in-person sessions, with occasional contacts by phone;<br>3. Mini-group interventions of two to seven participants, in which the wellness program instructor offers a more interactive version of the formal classes to a small group; and 4. Full-group classes of eight or more participants, in which a series of formal sessions are offered by the wellness instructor. The menu was used by the wellness counselors and instructors to avoid having to turn away employees who signed up for classes which did not attract enough people to make up a full group. When employees signed up for a formal program such as smoking cessation, they were placed in the type of group available at the time — full group class, mini-group, or one-to-one. Employees who preferred to make changes on their own were seen by the wellness counselors periodically in guided self-help.

	Program interventions were most comprehensive at Site 4. In addition to follow-up counseling and health education activities offered on the menu described above, plant organization activities were added to the study procedures at Site 4. Plant organization activities were jointly developed and managed by the wellness counselors and the wellness committee, and included the creation of informal health networks and peer support groups such as buddy systems, specific interest health promotion groups such as walking clubs, and plant-wide health promotion activities such as weight loss contests and plant-wide "smoke-outs." One aspect of these plant organization activities, the development of communication networks, has been described more fully elsewhere." The underlying rationale for this model recognizes that many behaviors associated with CVD risk are, in the short run, pleasant and functional for reducing stress or managing social situations. Thus the model emphasizes encouraging people to improve their health and wellbeing, and learning positive substitutes for behaviors that create long-term health risks. Because plant organization is by definition developed and carried out jointly with the plant employees, the specific activities listed above were developed during the project period. However, the general form and rationale for the intervention were incorporated in the initial study design.<br><br>All of the screened employees at Site 4 were considered to be eligible for the follow-up caseload, regardless of their CVD risk status. Attempts were made to contact employees with the three targeted CVD risks, plus employees interested in health promotion activities, at least once every six months. Two part-time wellness counselors, who maintained an office located in the personnel area of the plant but independent of the personnel department, handled the follow-up and plant organization activities at Site 4.
Fihn, 2004	The intervention consisted of synthesizing accumulated information and reporting it to the primary care providers. At all visits of enrolled patients, summaries of all applicable target conditions (up to six) were delivered to the clinician along with the medical record. For each condition, there were three elements: a graph displaying trended physiologic and questionnaire data (Figure 1), a narrative interpretation (e.g., "shortness of breath most of the time"), and a randomly selected management "tip" consisting of a brief extract from a relevant national guideline (not necessarily applicable to that patient at that time). These reports were designed through exhaustive pretesting of clinicians from all sites. An expert panel of specialists and primary care physicians was convened to determine the content of the reports for each target condition. Clinicians also received detailed summaries showing how the health status and satisfaction of their patients had changed over time compared with the average changes for their firm and for all patients in ACQUIP (Figure 2). Accompanying these reports were lists of patients who had extremely poor results for each condition (e.g., exceptionally severe angina or very high levels of alcohol intake), along with contact information. Throughout the study, local investigators held several sessions to train intervention providers in the principles of quality improvement and in the interpretation of health status measures using standardized materials formatted to accommodate the special characteristics of their site. An 18-month pilot test of the intervention was conducted at three sites and involved over 10,000 patients.
Froehlich, 2002	A comprehensive educational program was instituted for the implementation of the ACC/AHA Task Force guidelines. These educational tools were intended to change the understanding of the role of stress testing in the preoperative cardiac evaluation and to develop a consensus pathway for patients scheduled for elective aortic surgery. To this end, Cardiology Division grand rounds lectures were held to explain the guidelines and algorithms and the medical literature supporting their design. Similar lectures were held for the Section of Vascular Surgery and the Department of Anesthesiology at the University of Michigan. After these traditional educational measures, we implemented techniques designed to put guideline information and reminders in the hands of clinicians at the time of clinical decision making. A General Medicine preoperative screening clinic was established for the evaluation of patients undergoing elective surgery. A data collection sheet was designed for use in this clinic and included an outline of the preoperative evaluation algorithm. Laminated pocket-sized copies of the algorithm were produced and disseminated to the attending staff and housestaff as part of a monthly orientation for the consultation teams (Appendix, online only). In this way, preoperative evaluation was performed by a more focused group of clinicians, all of whom had been exposed to the algorithms and had printed copies with them when clinical decisions were made. Immediately after guideline implementation, the same demographic and outcomes data were gathered for the first 100 patients undergoing aortic surgical procedures at the University of Michigan. This process was repeated 2 years after guideline implementation to evaluate long-term effects. Data were analyzed comparing immediate and delayed group patients with historical control patients. Demographic characteristics were compared, including clinical risk factors, resource use, and clinical outcomes.
Gaede, 2003	We undertook a randomized study — the Steno-2 Study — to evaluate the effect on cardiovascular disease of an intensified, targeted, multifactorial intervention comprising behavior modification and polypharmacologic therapy aimed at several modifiable risk factors in patients with type 2 diabetes and microalbuminuria; we compared this approach with a conventional intervention involving multiple risk factors.<br><br>The aim of dietary intervention was a total daily intake of fat that was less than 30 percent of the daily energy intake and an intake of saturated fatty acids that was less than 10 percent of the daily energy intake. Light-to-moderate exercise for at least 30 minutes three to five times weekly was recommended, and all smoking patients and their spouses were invited to participate in smoking-cessation courses. All patients were prescribed an angiotensin-converting– enzyme (ACE) inhibitor in a dose equivalent to 50 mg of captopril twice

	daily or, if such a drug was contraindicated, an angiotensin II–receptor antagonist in a dose equivalent to 50 mg of losartan twice daily, irrespective of the blood pressure level. They also received a daily vitamin–mineral supplement containing 250 mg of vitamin C, 100 mg of d-*a*-tocopherol, 400 µg of folic acid, and 100 µg of chrome picolinate. Initially, 150 mg of aspirin per day was given as secondary prevention to patients with a history of ischemic cardiovascular disease, and after October 1999, all patients received aspirin (unless there were contraindications). If patients were unable to maintain glycosylated hemoglobin values below 6.5 percent by means of diet and increased physical activity alone after three months, an oral hypoglycemic agent was started. As the initial step, overweight patients (defined as those with a body-mass index [the weight in kilograms divided by the square of the height in meters] above 25) received metformin (maximum, 1 g twice daily); lean patients, or overweight patients who had contraindications to metformin therapy, received gliclazide (maximum, 160 mg twice daily). As the second step, metformin was added to the regimen of lean patients and gliclazide to that of overweight patients if hyperglycemia was not controlled. If the glycosylated hemoglobin value exceeded 7.0 percent despite maximal doses of oral agents, the addition of neutral protamine Hagedorn (NPH) insulin at bedtime was recommended. When insulin was started, lean patients stopped metformin treatment and overweight patients stopped gliclazide therapy unless it was the only oral hypoglycemic agent given. The insulin dose was adjusted on the basis of the morning fasting blood glucose concentration. If the daily dose of insulin exceeded 80 IU at bedtime or there was no decrease in the glycosylated hemoglobin value, patients were switched to regimens in which regular and NPH insulin was given two to four times a day (Table 2). Arterial hypertension was also treated with a stepwise approach. As mentioned, all patients were prescribed an ACE inhibitor or an angiotensin II– receptor antagonist because of the presence of microalbuminuria. If a patient had hypertension, thiazides, calcium-channel blockers, and beta-blockers were added as needed. The combination of an ACE inhibitor and an angiotensin II–receptor antagonist could also be used. Isolated instances of raised fasting serum cholesterol concentrations or combined dyslipidemia were treated with statins (atorvastatin, with a maximum of 80 mg daily, or the equivalent). Fibrates were used for isolated cases of hypertriglyceridemia, defined by a fasting serum triglyceride concentration of more than 350 mg per deciliter (4.0 mmol per liter), or were added to statin treatment if the fasting serum triglyceride concentration was also elevated (350 mg per deciliter).
Gary, 2003	There were four arms: (1) usual medical care (control); (2) usual medical care _ NCM intervention; (3) usual medical care _ CHW intervention; (4) usual medical care _ NCM _ CHW (combined team intervention). Interventions began as soon as possible after randomization and continued up to the point of the 2-year outcome assessment visit.<br><br>**Usual medical care only**<br>Participants assigned to the usual medical care (control) group continued on-going care from their own health professionals. In addition, they received a quarterly newsletter, which contained information on various diabetes-related health topics and on-going trial communication (e.g., baseline demographic profile of all participants).<br><br>**Nurse case manager intervention**<br>The NCM was a registered nurse with a baccalaureate degree in training to be a certified diabetes educator. NCM interventions were 45-min face-to-face clinic visits and/or telephone contacts. The NCM coordinated care according to the American Diabetes Association (ADA) Clinical Practice Recommendations for all participants assigned to the nursing and the combined NCM/CHW group. She provided direct patient care, management, education, counseling, follow- up, referrals, and physician feedback and prompting, which included advising regimen changes and implementing changes under physician's orders. The goal was to conduct visits approximately three times per year, plus additional contacts as needed.<br><br>**Community health worker intervention**<br>The CHW was a local high school graduate who was enrolled in college part time, and had no formal training in health care before the study. CHW interventions were 45- to 60-min face-to-face home visits and/or telephone contacts. Unlike the NCM, the CHW did not directly implement therapeutic strategies (e.g., recommend change in medication doses). Rather, the CHW facilitated preventive care by offering to schedule appointments and visits, along with providing education. Her main responsibilities were to monitor participant and family behavior, reinforce adherence to treatment recommendations, mobilize social support, and provide physician feedback, which included reporting on identifiable problems such as high blood pressure readings or dietary habits. The goal was to conduct visits approximately three times per year, plus additional contacts as needed.<br><br>**Combined NCM/CHW intervention**<br>This intervention combined the individual activities of the NCM and CHW. In addition, the two interventionists conducted biweekly conferences to coordinate interventions and promote synergy. The goal was to conduct approximately three visits per year with the NCM and three visits per year with the CHW, plus additional contacts as needed.
Getpreechasw as, 2007	**Part I (August 2002-July 2003)**<br>Guideline of the Procedures is shown in Fig. l. A baseline measurement of outcome variables was carried out in August 2002 (Measure I). At the beginning of the present study in mid-2002, health personnel in the areas where the three PCU s serve were vigorously trained by the researchers on approaches to prevent and control hypertension using VHVs and

	FHLs. The researchers and health personnel then later trained VHVs and FHLs on possible interventions for prevention and control of hypertension over a year. Interventions used in Groups I and II included several community-initiated activities such as exercise clubs, health parks, and self-help groups. The outcomes variables were measured one year after the intervention activities had been initiated (Measure 2). After this, two intervention groups filled this hypertension program into their routine work.<br><br>**Part II (October- December 2004)**<br>A cost-effectiveness study was carried out to compare costs (operating costs and programming cost) of the program and the outcomes of the program in knowledge, eating behaviors and physical activities.<br><br>**Part III (November2005 -April 2006)**<br>This part was carried out to assess sustainability of the program. About three years after the program commencement, the third round of measurement of outcomes variables (Measure 3) took place in November 2005. In addition, levels of participation of VHVs and FHLs in Groups I and II were assessed by evaluation of satisfaction of hypertensive patients and other people in each group. In addition, focus group discussions were held among health personnel, representative of VHVs, and FHLs to collect their opinions regarding success, failure, and applicability of this kind of program in this and other wider contexts.
Giannuzzi, 2008	**EXPERIMENTAL INTERVENTION**<br>The multifactorial, continued educational and behavioral program was performed by a cardiac rehabilitation team composed of a specialist cardiac nurse, a physiotherapist, and a cardiologist (who was the supervisor). A clinical psychologist and occupational therapist could be recruited if needed. A letter was sent to patients' family physicians informing them of the study and inviting their collaboration in the study objectives. Comprehensive cardiac rehabilitation sessions with one-to-one support were held monthly from month 1 to month 6, then every 6 months for 3 years. Each session consisted of 30 minutes of supervised aerobic exercise, plus lifestyle and risk factor counseling lasting at least 1 hour and reinforcement of preventive interventions lasting approximately 30 minutes. To improve adherence to lifestyle modification and help patients adopt a positive role in the care of their own health, a booklet explaining how to deal with exercise, diet, smoking cessation, and stress management was distributed. The mutual support of family members (eg, spouses) was encouraged in ad hoc meetings together with the patients to make correct lifestyle habits more likely to be maintained in the long run. The intervention was aimed at individualizing risk factor and lifestyle management, and pharmacological treatments were based on current guidelines.3,5 The targets of the intervention strategy were to give up smoking, adopt a healthy Mediterranean diet, increase physical activity up to at least 3 h/wk at 60% to 75% of the mean maximum heart rate, maintain body mass index (BMI) (calculated as weight in kilograms divided by height in meters squared) of 25 or less, blood pressure of 140/85mmHg or lower (130/80mmHg for patients with diabetes mellitus [DM]), total cholesterol level of 200 mg/dL or lower, low-density lipoprotein (LDL) cholesterol level lower than 100 mg/dL, blood glucose level of 110 mg/dL or lower, and hemoglobin A1c (HbA1c) level lower than 7.0% in subjects with DM.3-5,20,21 Pharmacologi-cal treatments, including antiplatelet agents, angiotensinconverting enzyme (ACE) inhibitors or angiotensin-II receptor blockers (ARBs), _-blockers, statins, and omega-3 polyunsaturated fatty acids, were positively recommended to all patients. (To convert total cholesterol and LDL cholesterol to millimoles per liter, multiply by 0.0259; to convert HbA1c to a proportion of total hemoglobin, multiply by 0.01.)<br><br>**CONTROL GROUP**<br>A letter was sent to family physicians recommending secondary prevention goals. After the standard, post-MI CRP, patients in the usual care group reported to their reference center only to undergo the 6-month and then annual scheduled assessments. After each assessment, a copy of the results of laboratory and exercise tests was forwarded to the family physician who was responsible for any further medical decisions.
Gibson, 2011	**Program Intervention**<br>On January 1, 2006, the firm implemented a diabetes disease management program for those covered under its medical plan. The program was voluntary, so that people who did not want to participate could opt out. Like similar plans offered by most major employers, the program consisted of targeted mailings, a workbook about the disease, telephone outreach by a nurse, additional educational mailings, coaching, and periodic monitoring. An initial letter to employees explained the program's components, which did not change during the course of the study. Participants received additional communications reinforcing the diabetes management goals (such as testing for HbA1c, or glycemia) as well as medication adherence for the duration of the study. At the same time, the firm offered its employees and their dependents in two large, US-based units a diabetes value-based pharmacy design program. In all, 33,160 people were eligible for this program. The two programs were administered separately, and the vendor of the disease management program did not know which beneficiaries enrolled in the pharmacy program. The pharmacy program lowered coinsurance for all diabetes medications to 10 percent, from a tiered structure that had charged coinsurance ranging from 10 percent for generic medications to 35 percent for nonpreferred brand-name drugs. (See Appendix Exhibit 1 for a list of the diabetes

	medications.)<br><br>Our baseline year was 2005, before the intervention began. Each subsequent year (2006, 2007, and 2008) was in the post-intervention period. We included in our study all enrollees under age sixty-five who had at least four consecutive quarters of enrollment in the period 2005–08. We focused our analysis on the effects of the value-based pharmacy program in two groups of enrollees with diabetes: those who participated in the disease management program, and those who opted out of it. We considered these groups separately to avoid the possibility of selection bias, which could arise because patients with diabetes who chose to participate in disease management might be different from the patients who opted out. These variations in program implementation also allowed us to exploit the presence of a comparison group within the firm: the 59,038 enrollees in its medical plan who worked in business units where the value-based pharmacy plan was not offered. We matched enrollees in the value-based program and the disease management program with similar enrollees in the disease management program only. In addition, we matched enrollees with diabetes in the value-based program only to enrollees with diabetes who were not in either program. To match enrollees, we used a summarized propensity score. We first estimated a propensity score—or the probability of being in a specific program—based on certain variables for each enrollee. These were sociodemographic variables (age, sex, census region, residence in an urban or rural area, relationship to the employee, employee classification, employment status, median income of the ZIP code of residence, and percentage of college graduates in the ZIP code of residence); plan type; health status according to the Charlson Comorbidity Index, a measure of how many chronic conditions a person has, and the number of psychiatric diagnosis groups for each employee; and the length of enrollment by number of quarters. Then we matched enrollees in the programs with enrollees in the relevant comparison group, as described above, according to the propensity score. To obtain the best matches, we required a close match in propensity scores between the enrollees in the program and their counterparts from the comparison group. We constructed a panel data file with each enrollee as the cross-sectional unit and each calendar quarter as the unit of time. We captured enrollees' experience in quarterly increments through the end of their participation in a particular program or through the end of December 2008, whichever was later. We continued to collect data on enrollees who switched plans.<br><br>**Medication Use And Adherence**<br>We calculated the medication possession ratio—which can range from 0 percent to 100 percent—based on the percentage of days that an enrollee had his or her prescribed medication available within each quarter. We used the dates when prescriptions were filled and the number of days supplied on the prescription drug claims to determine how many days' medications were on hand. We calculated the ratio separately for oral antidiabetic medications and insulin. Information about filling prescriptions prior to 2005 was not available. Because the number of days that medications were on hand early in 2005 is probably the result of prescriptions filled in 2004, we did not include the possession ratios for the first quarter of 2005 in our analyses. Had we done so, our results would probably have understated patients' adherence to their medication regimens. As a second measure of drug use, we calculated the percentage of patients who had medication on hand for at least 80 percent of the days in each quarter. This level is generally accepted as a threshold for clinical benefits to occur. Patients at or above this level are considered to be adherent, while those below this level are considered to be nonadherent.<br><br>**Use Of Diabetes Guidelines**<br>We created a set of indicator variables to measure the percentage of enrollees receiving medical services recommended by the guidelines in each quarter. The services included three laboratory exams—tests for HbA1c, lipid tests, and urinalysis—and professional services such as visits to a primary care physician and eye exams. (See Appendix Exhibit 2 for a full list of the services.)<br><br>**Payments**<br>In this study we defined *payments* as the allowed or actual total reimbursement that the provider of care received. Payments could come from the patient; his or her health plan or employer; or another payer, such as a spouse's employer, through "coordination of benefits" procedures to determine which plan should bear the costs.<br>We calculated payments for all inpatient and outpatient medical services, prescription drugs, and any combination of medical services and prescription drugs. We also calculated payments for diabetes-related services and for all conditions, including diabetes. (See Appendix Exhibit 1 for a list of diabetes-related prescription drugs.)
Grosbois, 1999	Outpatient respiratory RP was conducted at a rehabilitation hospital. The program comprised three sessions per week over a period of 7 weeks, with as many as 6 patients per session being supervised by two physiotherapists and one pulmonary physician. Each session spanned a 2-hour period and consisted of the following components:<br><br>• Patient education about the course of the disease and optimal medications use in COPD;<br>• Coughing techniques;

	• Progressive muscle relaxation;<br>• Breathing retraining to improve thoracic and abdominal synchronization; and<br>• Exercise reconditioning.<br><br>Exercise reconditioning incorporated lower limb exercises such as walking and cycle ergometry. Endurance training intensity on the bicycle initially was targeted at a workload corresponding with the patient's ventilator threshold. Exercise intensity was increased gradually as tolerated and time span at this intensity level progressively extended from 30 to 45 minutes during the RP. During the sessions, oxygen saturation (SaO2) was monitored and additional oxygen was provided if SaO2 fell below 90%. After the completion of the program, all patients received instructions to continue the exercises at home once a day for 30 minutes. The intensity of these exercises were individualized based on postoutpatient respiratory RP performances and progress during training sessions. Patients were instructed to adjust the training intensity on heartbeat, as measured at ventilatory threshold during exercise testing at the end of RP. In addition, all patients were proposed to participate in a structured EM program, either once or twice a week. These EM sessions were supervised by one physiotherapist and spanned a 2-hour period in the structure where initial RP took place. The format of exercise training in the EM sessions was similar to initial RP, with the intensity and duration adjusted to the patients' performances toward the completion of initial RP and progress during training sessions.
Hedges, 2000	An educational program emphasizing patient, family, and provider recognition of AMI symptoms and desired actions was developed. 6 The intervention targeted community organizations and the general public, high-risk patients, and health professionals.5 The primary objective of the intervention was to reduce delay in seeking care among patients with signs and symptoms of acute cardiac ischemia. The intervention messages and target groups were based on review of the published literature and focus-group analyses performed prior to baseline data collection. The primary educational message emphasized recognition of chest pain and other ischemic symptoms, with action if symptoms persisted for 15 minutes or more. Multiple media channels were used to deliver the message to the general public.6 Physicians, nurses, paramedics, and other health care providers helped deliver the message to high-risk groups. After a four-month baseline period, the intervention was implemented in one randomly chosen community from each matched pair. The public education component of the intervention sequentially emphasized different themes (i.e., general awareness of AMI symptoms and the need for rapid action, development of a Heart Attack Survival Plan, AMI in women, variability of AMI symptoms, bystander response to heart attacks, and use of 9-1-1) to reinforce the primary message. These themes were sequentially introduced over an 18-month period by community interventionists to help sustain awareness of the campaign and primary message.
Hess, 2007	**Study 1: Research Staff Intervention (April 2002 to August 2003)**<br>The study design and participant flow are depicted in Figure 1. Trial participants were long-term customers (1 year of barbershop patronage) with persistent HTN after 3 sequential on-site screening visits. Condition assignment was nonrandom and based on staffing requirements; the enhanced intervention group was recruited from 1 large barbershop and a contemporaneous comparison group from 2 smaller shops. Both groups received written results of the 3 BP screenings and standard recommendations for interval medical follow up. For the next 8 months, the comparison group also received a continual supply of the American Heart Association brochure titled *High Blood Pressure in African Americans* (product code 50–1466), whereas the enhanced intervention group received the theory based intervention described below.<br><br>*Behavior Theory–Based Intervention*<br>Social cognitive theory drove the intervention depicted in Figure 2. With accurate electronic BP monitors and medical research personnel in the barbershop as environmental facilitators, intervention messages emphasized the gravity of the personal health risk from elevated BP and the need for a regular medical provider, prescription medication, and continuous BP monitoring for effective risk reduction. The intervention was conducted by black research assistants and medical/premedical students supervised by a black nurse who was either on-site or available by telephone to facilitate referral to community physicians. Based on individual insurance policies, participants were referred either to physician offices within walking distance of the barbershop or to a physician within their provider networks. Uninsured low-income participants were referred to the Parkland Health and Hospital System. Patients with undertreated HTN were referred back to their established providers. The main intervention tools were "BP report cards" giving customers and their providers on-going feedback about the need to initiate or intensify antihypertensive therapy and role model stories depicting successful risk reduction strategies adopted by other members of the target community with whom study participants could readily identify or real hypertensive customers in the intervention barbershop (please see the data supplement for these materials, available online at http://hyper.ahajournals.org). Discounted haircuts ($6 off the regular price of $12) were provided as an incentive for continued intervention participation.<br><br>*BP Measurement*

	All of the BPs were measured in the barbershops with validated electronic oscillometric monitors (Series 52 000, Welch Allyn) after 10 minutes of rest using an appropriately sized arm cuff with the participant seated in a barber chair. At each encounter, 4 consecutive BP readings were taken, and the last 2 readings were averaged to calculate a BP value; 2 sets of readings on separate days were averaged to calculate initial and final BP values for each participant.<br><br>**Study 2: Barber Intervention (December 2003 to March 2005)**<br>Under nurse supervision, the barbers in one of the previous comparison shops from study 1 conducted the intervention outlined in Figure 2, with minor refinements explained in the figure legend. All of the adult black male customers were eligible to participate in a continuous onsite BP monitoring and referral program. Training and supervision of barbers, as well as encounter forms and other intervention materials, are available in the online data supplement. Financial incentives to the barbers were \$3 per recorded BP and \$50 for each BP report card signed by a medical provider and returned to the barber with proof of a new BP prescription.
Higginbotham, 1999	**Program development and implementation**<br>Insights provided by A. Metcalfe's work were considered essential to understand local responses to the CHHB, to tailor program activities to local interests and to try and avoid reproducing past injustices by inadvertently using a `healthist' discourse that would simply transform Coalfields people from the historical image of the `uncouth' to the contemporary slur of the `unhealthy' (Metcalfe, 1993). Early consultation with the Coalfields community included a mail-administered survey of community needs (Higginbotham et al., 1993). A systematic random sample of 919 residents drawn from the federal electoral roll were mailed the questionnaire eliciting their degree of worry about 17 issues (e.g. crime, drugs, money, having a heart attack). Responses from the 435 Coalfields residents1 completing the needs survey, along with insights from a health ideology questionnaire administered later, were combined with the outcomes from further consultation with local opinion leaders and community health workers to guide the project. The Health Ideology Instrument (HII) was interviewer-administered to 402 adult Coalfields residents randomly selected from the federal electoral roll (Heading, 1996, pp. 274±305). The HII asked residents to indicate their strength of agreement with 21 pre- coded statements about food, dieting, body image and one's health. The instrument contains three subscales measuring distinct modes of responding to dominant health promotion discourse: Acceptance of, Pragmatism toward and Rejection of health messages. In 1990, the University researchers and program coordinator organised two widely publicised public meetings to announce the initiative and identify interested local leaders, as well as list targets and strategies for change and gain consensus on these. A steering committee formed to help guide the program and met monthly with the program coordinator. The coordinator supported the steering committee, assisted with the development and implementation of community activities, and worked at raising local awareness of heart disease rates and fundraising to support the program. A vital first step was merger with the new initiative of the existing healthy heart support group (begun in 1986), including its high profile community members (former mayor and retired mining union leaders).<br><br>**Process evaluation**<br>Process measures used to determine the impact of the CCHB focus on the number and range of program activities promoting knowledge and awareness of heart health, interest and participation in activities by local residents and the development and sustainability of activities.<br><br>Awareness raising & public relations (began 1986)<br>• Media releases<br>• Public displays<br>• Heart health promotions (e.g. heart week)<br>• Guest speaking at clubs, schools, worksites<br>• FM radio broadcasts (ongoing)<br>Mobilising community resources (1990)<br>• Local management committee<br>• Rehabilitation research<br>• Healthy communities program<br>• Community consultation meetings (yearly)<br>• Incorporating heart health into community events: fun run, motorcycle race, agricultural college events, tidy towns program, high school health expo.<br>Heart health rehabilitation (since 1992)

	• Heart Support - Australia, branch office (40 regular<br>• members)<br>• Rehabilitation exercise program (150 users)<br>• Telephone counsellors trained (>45 counsellors)<br><br>Promoting healthy lifestyles<br>• Cooking classes (since 1993; 200 users)<br>• Lowfat cooking class<br>• Healthy cooking for families<br>• Healthy cooking demonstration<br>• Supermarket tours (nutrition education)<br>• Weight control (1994; 20 users)<br>• Regular classes<br>• Classes targeted at the obese<br>• Gentle exercise (1993; >200 users)<br>• Tai Chi classes<br>• Regular classes with child care provided<br>• Walking for pleasure Ð walking trails book published<br>• Anti-smoking programs (1994)<br>• QUIT<br>• Adolescent QUIT<br>Institutional and environmental development<br>• Walk-in Heart Health Resource Centre (opened 1993)<br>• Schools (Launched 1992)<br>• Healthy heart schools network (15 primary and 2 high<br>• schools)<br>• Health promoting schools (alcohol awareness, sun protection, quit smoking programs at three high schools).<br>• Restaurants, clubs and Retailers (1993)<br>• 'Healthy eats' restaurant program (10 clubs and restaurants))<br>• Fast food outlet accreditation (six shops)<br>• Reduction in access of minors to cigarettes campaign<br>• Industry programs (1991)<br>• Aluminium smelter<br>• Garment manufacturing<br>Mobilising institutional resources<br>• University research and educational resources (1990)<br>• Regional assistance scheme funding (began 1994)<br>• CHHB ongoing funding by NSW Health Department (1996)
Hopper, 1984	The intervention was carried out for 18 months between January 1979 and June 1980. The 227 subjects were randomly allocated to Group A (n = 113), who continued to receive routine care, or to Group B (n = 114) who were offered home health aide services. Of the group offered aides, 44 (39 per cent) accepted and received aides throughout, 39 (34 per cent) continued to attend clinic but refused aide services, and the remaining 31 (27 per cent) withdrew altogether from clinic services at some point during the 18-month intervention.

	Withdrawal from all clinic services was similar in the group not offered aides (33, 29 per cent).* The four aides were Black women and city residents, trained by staff from the hospital's Medicine, Dietary, and Physical Therapy Departments, and the Medical Nurse Practitioner Program, which also provided supervision. The 13-month training program consisted of four months of didactic home health education, a six-month practicum of supervised home health visiting, and three months of additional intensive training in diabetes management including practicums in the podiatry and surgery clinics. Skills developed by aides included collecting physical and historical health data, performing limited diagnostic or therapeutic procedures (including monitoring blood pressure, weight and pulse), administering insulin, and performing routine patient care such as bathing and feeding. Additionally, aides were taught communication skills in order to present recommendations for basic health and hygiene and to convey basic medical and dietary instructions. Initial assessment included measures of fasting blood glucose levels (FBS) collected at each clinic visit (mean of 6 visits ± 3), a medical record review of health care utilization, an assessment of diabetic complications by medical nurse practitioners prior to intervention,** an aid checklist of services performed (collected at each visit), and a pre-intervention interview by research staff on social and attitudinal variables.
Hughes, 2010	**Exercise program, negotiated maintenance with telephone reinforcement**<br>All participants enrolled in the evidence-based 8-week Fit and Strong! program described above that combines flexibility/balance, aerobic walking, and strength training with health education for sustained behavior change. Eighteen instructors: (6 licensed physical therapists and 12 certified exercise instructors) were trained and implemented the program at 7 senior centers over the 4 years that Fit and Strong! was offered.<br><br>**Negotiated maintenance with telephone reinforcement (TR)**<br>Participants in the negotiated arm met with the Fit and Strong! instructor between weeks 6 and 8 to develop individualized, negotiated follow-up plans for physical activity maintenance. These meetings systematically explored participants' preferences for type, time, and location of follow-up physical activity. The protocol for participants in both the negotiated and mainstreamed study arms who received TR specified the receipt of 2 phone calls per month in months 3-6 post- Fit and Strong! and one phone call per month between months 7 and 18. During the month-18 phone call, participants were given a hotline number to call for assistance if needed during the following 6 months. All phone calls asked whether participants were still exercising, what they were doing, and explored barriers and facilitators to exercise. Phone discussions were brief, lasting about 10 to 20 minutes per call. TR was conducted by the master's-level project manager and 4 graduate students using instruments created by the research team to conduct and document each call. All study staff received training before administering calls. Maintenance of physical activity and associated outcomes were assessed at 2, 6, 12, and18 months.
Huizinga, 2010	We conducted a randomised controlled trial to assess the efficacy of a 2 year programme to prevent glycaemic relapse in patients with recently controlled type 2 diabetes. The study protocol has been previously described [10]. Following completion of a 3 month intensive diabetes improvement programme (DIP), participants with a ≥1% decrease in HbA1c were randomised to the following maintenance regimens: (1) routine follow-up in a primary care clinic (control); (2) routine follow-up with quarterly telephone contact; or (3) routine follow-up with monthly telephone contact.<br><br>The intervention consisted of a phone contact by a nurse practitioner with referral to a dietitian if nutrition self-care was perturbed. The nurse practitioners and dietitians were all certified in diabetes education (Certified Diabetes Educators [CDE] or Advance Diabetes Management– Board Certified [ADM-BC]) and had significant clinical experience in providing diabetes care. For the study, the nurse practitioners adhered to a set of intervention protocols and guidelines, which have been described in detail in a separate manuscript. Providers helped participants titrate medications, and identify and solve problems arising in self-care behaviours, including diet, exercise, self-monitoring of blood glucose and medication adherence. If no problems in self-care behaviour were identified, anticipatory planning, positive reinforcement and mutually established goal-setting were performed. If a problem in a self-care behaviour was identified and the participant was able to identify the source of the problem, then a standard problem solving paradigm was employed. If the source of the problem could not be identified, then the provider either engaged in motivational interviewing to further explore the problem or compensated for failure to identify the problem by altering another self-care behaviour as a temporary measure until the source of the original problem could be identified. Only the frequency of the intervention varied between treatment arms.
Inglis, 2006	**Usual Patient Management**<br>All patients were subject to usual levels of post-discharge planning as employed in the cardiology and medical units at the time of recruitment to the study (1995–1998). No\ restrictions were placed on the extent or the intensity of follow-up. Typical follow-up included an appointment with their primary care physician and the cardiology outpatient clinic within 14 days of discharge. All patients underwent regular outpatient-based review by a cardiologist at the hospital and attended their same primary care clinic.

	**Home-Based Intervention**<br>The rationale and specific details of the study intervention have been described more extensively in previous publications (also available on request from S.S.) and arose from our group's wider interest in the value of home-based, chronic disease management. Consistent with the reality of dealing with patients with multiple disease states and many different preventable reasons for recurrent events while recognizing the need to deal with the most "high-cost" users of the healthcare system, we specifically focused on CHF patients and designed an intervention that would deal with all their potential healthcare needs (hence our focus on all-cause events). Our eclectic approach was built on earlier research relating to comprehensive geriatric assessments, the application of a broad range of adult learning theories relating to life-long learning, and the principles of individual and community empowerment to facilitate self-determination (in this case, self-care). In essence, patients assigned to HBI received the same level of care as those assigned to UC plus the prospectively designated study intervention. As such, HBI comprised a structured home visit within 7 to 14 days of discharge, by a nurse and pharmacist or by a qualified cardiac nurse. During the home visit, patients underwent a physical examination and a review of their adherence to and knowledge of their condition and prescribed treatments as well as an assessment of their social support system. Factors likely to increase the immediate and longer-term probability of hospital readmission or death were identified. For example, home visits revealed that close to 40% of patients were found to have undiagnosed early clinical deterioration and often an impaired ability to recognize signs of an impending crisis. Moreover, up to 50% of patients exhibited poor self-care behaviors and/or were consuming potentially harmful agents (eg, a nonsteroidal anti-inflammatory agent). On the basis of this comprehensive home assessment, patients and their families (if appropriate) received a combination of remedial counseling, introduction of strategies designed to improve treatment adherence, introduction of a simple exercise regimen, and incremental monitoring by family/caregivers. Those with signs of clinical deterioration were immediately reviewed by their primary care physician or cardiologist, and remedial action was taken. Those with problems in managing their medications were referred for long-term support by their community pharmacist. Irrespective of the outcome, a comprehensive report was sent to the patient's primary care physician and cardiologist detailing both the assessment and any actions taken or recommended. All patients were subject to telephone follow-up over 6 months to ensure that patients were receiving appropriate levels of support, and the patient's physicians and/or community services were contacted to address any problems. Over the same time frame, 25% of patients initiated telephone calls for advice and/or to arrange an urgent Review. Both short-term (intensive) and long-term (predominantly routine and surveillance) management strategies were therefore applied as part of the HBI. The only clinically important differences between the form of HBI applied in these 2 studies (except the personnel involved) were that the first cohort received some additional information about their condition and treatment during their index admission (although this was shown to have minimal effect on outcomes) and, in the second cohort, 7 patients received repeat home visits if they survived a readmission within 6 months.
Jia, 2009	Within the Department of Veterans Affairs (VA), a transition from a hospital-based system to one that emphasizes ambulatory and patient-centered care has occurred. The national VA Care Coordination Home Telehealth (CCHT) program was developed as one transforming strategy to improve accessibility and provide timely and appropriate care for community-dwelling veterans with chronic diseases, such as diabetes mellitus (DM), and complex needs for multiple adverse conditions. This approach is achieved through care coordination by nurse practitioners or registered nurses and disease monitoring with the use of supportive home telemonitoring technology. Patients enrolled in the VA CCHT program receive a home telehealth device (e.g., a messaging device installed at home that requires basic landline telephone service and an electrical outlet). On a daily basis, CCHT enrollees answer scripted questions from the messaging device about their symptoms and health status. The care coordinators monitor patients' daily updates from the devices and take follow-up actions such as (1) placing a telephone call to the patient, (2) arranging a referral to the patient's physician or scheduling new appointments with VA clinicians as needed, (3) placing new orders for patient medications, (4) helping patients manage their medications, (5) reminding patients of their clinic appointments, and (6) aiding with technology difficulties. A detailed description of the CCHT program can be found elsewhere.<br><br>This was a retrospective matched treatment-control study. The treatment group consisted of 387 veterans who were diagnosed with DM and enrolled in the CCHT program at four VA medical centers (VAMCs) located in a single Veterans Integrated Service Network (VISN) in the southern region. The inclusion criteria for the CCHT program included (1) > 1× use of VA inpatient or emergency services within the 12 months prior to enrollment date, (2) noninstitutionalized at enrollment time, and (3) a telephone landline at home. The control group consisted of 387 VA DM patients from the same VAMCs as the CCHT program enrollees who had not received or been approached by the telehealth service; control subjects were chosen from a pool of patients by a propensity score. Patients in the treatment group were randomly matched on the basis of the CCHT programs' enrollment dates and patient care settings so that both groups (treatment and control) had the same allocations of enrollment and service period. Further, to improve the quality of the match between the treatment and control groups, we applied the propensity score method to enhance the balance between the different groups of patients. The propensity score is a single summary score of a patient's background characteristics and it represents the probability that a patient belongs to a naturally occurring treatment group. As such, it has a distinct advantage over standard matching techniques. For instance, it has been used extensively in medical and health services research that uses survey or observational data, and it has the potential to reduce selection bias. Three controls were randomly selected for each study participant of the treatment group to guarantee an adequately sized control group. The propensity score-based matching was performed by (1) estimating the probability that a patient is "chosen" into the treatment group as opposed to the control group, (2) separating the sample into

	quintiles of the predicted propensity scores distribution (<20%, 20%–40%, 40%– 60%, 60%–80%, and 80%–100%), and (3) randomly sampling the controls with a size equal to the number of cases within each quintile. The veterans were selected to fit our outcome models, and more detailed information about this method is published elsewhere.
Jovanovic, 2004	Blood glucose management<br>• Self-monitoring of blood glucose (SMBG): individual education and ongoing assessment to reach individually defined targets.<br>• Testing frequency: as needed to meet treatment goals, generally at least two times per day.<br>• Glucose records: maintained by participant; results reviewed each visit and compared with meter memory.<br>• Data review: SMBG trends/patterns identified; collaboration with primary care provider to modify treatment.<br>• Treatment: strategies individualized to meet goals.<br><br>Nutrition education and management<br>• Weight: measured at every visit.<br>• Treatment: individualized meal plans to meet nutrition and weight-management goals.<br>• Routine assessment: content, quantity, and timing of food intake; adjustments as needed.<br><br>Exercise<br>• Assessment of physical activity: at least quarterly.<br>• Exercise stress testing: when advisable/available.<br>• Exercise plan: incorporation of current activity preference and level; increased as tolerated.<br><br>Foot care<br>• Visual inspection/examination at least quarterly.<br>• Education: daily self-inspection and preventive care.<br>• Referrals for specialty care: as needed.<br><br>Monitoring of participant progress and retention<br>• Record sheets: developed to promote ongoing participant assessment and provider communication (monitor appointments, physical measurements, laboratory values, SMBG results, active problems, treatment, etc.).<br>• Retention strategies: *1*) interim visit telephone contact; *2*) appointment reminders/tracking/rescheduling; *3*) group education/social activities; *4*) holiday/special greeting cards.<br>• Written record of participant interactions: shared with primary care providers to ensure continuity and quality of care.<br>• Community support: family/significant others encouraged to attend appointments and events.<br>• Communication: bilingual study staff and native language print materials used when possible.<br>• Interpretation services: telephone company interpretation service; bilingual clinic staff assistance.<br>• Staff reassignment: as needed to optimize participant interactions, care, and retention.<br>• Ongoing self-management assessment: provide or refer for diabetes education, nutrition, and/or exercise guidance, psychosocial support, or community assistance resources.<br><br>Retinopathy prevention/treatment<br>• Retinal examinations and/or retinal photographs: at least yearly.<br>• Ophthalmologic follow-up: direct referral to an ophthalmologist; results obtained and forwarded to primary care provider.

	Nephropathy prevention/treatment<br>• Microalbumin: assessed at least yearly.<br>• Results: abnormal results flagged, primary care provider notified.<br>• Prevention/treatment: optimize blood glucose and blood pressure control; initiation of ACE inhibitors.<br><br>Hypertension management • Blood pressure measurements: at every visit.<br>• Target: _135/85 mmHg; more frequent monitoring and treatment if target exceeded.<br>• Treatment: diet and exercise modifications, weight management, pharmacological therapy.<br><br>Dyslipidemia management • Fasting assessment: at least yearly.<br>• Target: total cholesterol _200 mg/dl, LDL _130 mg/dl, triglycerides _150 mg/dl.<br>• Treatment: dietary and exercise modifications; pharmacological therapy as indicated.<br><br>Cardiovascular disease prevention/treatment<br>• Risk factor assessment: at least yearly.<br>• Smoking cessation: encouraged; referral to community programs.<br>• Weight management: encouraged; referral to community-based dietary counseling or programs.<br>• Aspirin and hormonal replacement therapy: initiated when appropriate.<br><br>Disenrollment from the study<br>• Criteria for early termination:<br>o withdrawal of consent;<br>o inability to keep appointments or respond to oral/written contact for 6 consecutive months;<br>o loss of Medi-Cal beneficiary status;<br>o geographic relocation;<br>o death
Kelso, 1996	**Educational Interventions**<br>On enrollment in the study, each patient received 1 hour of asthma education from one of the doctor of pharmacy investigators. Education was provided to individual patients versus group educational sessions. Educational content was consistent with the recommendations of the NIH, stressing that asthma is primarily an inflammatory condition and that inhaled antiinflammatory medication is absolutely critical to successful long-term management in moderate to severe disease. The four major components of asthma management were covered with each patient, including (1) environmental control, (2) objective monitoring at home with peak flow meters, (3) asthma education and partnership 1 between the patient and the health-care professionals in our clinic, and (4) medications. Throughout the initial encounter and subsequent visits, the concept of asthma prevention was stressed. Patients were told with enthusiasm, "Your asthma can be controlled: expect nothing less!" and the NIH National Asthma Education Program booklet with this title was given to each patient. Asthma triggers for each patient were reviewed, with techniques to eliminate them or minimize exposure. An Assess peak flow meter (HealthScan, Cedar Grove, NJ) was given to each patient with green (80-100% of personal best), yellow(50-80%), and red (<50%) zones established, including colored stickers placed directly on the peak flow meter. The investigator would always demonstrate use of the peak flow meter and have the patient demonstrate correct use on the initial and each subsequent visit. With each visit and telephone contact, the investigators made a concerted effort to maintain a partnership with the patient. As part of showing a genuine concern for each patient, active listening was an important part of our clinic. On each visit, we would ask the following question: "What is bothering you the most about your asthma right now?" Patients were involved in decision making with regard to therapy and how they felt about changes (eg, dosage adjustments). As part of self-management, patients were taught how to deal with increased symptoms early. In addition, each patient was given written instructions about crisis management (eg, procedure to follow if peak expiratory flow rate (PEFR) is in the "red zone"). Education with regard to medications received great emphasis in our program. Special care was given to stress the absolute importance of inhaled corticosteroids. Patients were told that their inhaled beclomethasone was *the key* to optimal long-term control of asthma, and that it must be used every day at the dose prescribed without ever missing a dose. An albuterol metered dose

	inhaler was given to each patient to prevent exercise-induced asthma and to treat symptoms "as needed." If two puffs of albuterol were needed more than three times daily, patients were instructed to contact the investigators for a dosage adjustment of their inhaled beclomethasone. Other medications that were required were explained to each patient verbally and in writing. Inhalation technique was carefully taught on the initial visit and checked on each subsequent visit. Teaching included discussion of each step, demonstrations by the investigators, and observation of each patient's technique on each visit. Patients were told that correct technique was vitally important to successful therapy. The frequency of clinic visits in the study group was initially every month, but as patients improved, we saw most patients every 2-3 months. Obviously, a few patients needed more frequent visits, whereas others did so well that visits were less frequent. Based on a review of hospital medical records in the control group, we could not determine the frequency of office visits.<br><br>**Therapeutic Interventions**<br>Inhaled beclomethasone (Beclovent, Allen & Hanburys, Research Triangle Park, NC) was the only oral inhalation corticosteroid used in this study and was always administered via an Aerochamber. Individualized dosing of the inhaled corticosteroid was viewed by the investigators as being of major importance in optimizing therapy for these patients. Each patient's dose was tailored based on symptom control as well as peak flow monitoring and inhaled albuterol requirements. The maximum dose used in our clinic was 40 puffs daily (42 µg/puff). Albuterol metered dose inhaler was used "as needed" and before exercise. An emergency supply of prednisone (40 mg daily for 3 days was based on the Mayo et al 4 study) was given to each patient. If the PEFR was in the "red zone" and two puffs of albuterol did not bring the PEFR back to at least the "yellow zone" in 15 minutes along with some subjective relief, the patient was to call the investigators immediately and start the emergency supply of prednisone. Early use of systemic corticosteroids was clearly shown to prevent further dangerous escalation of asthma symptoms in many patients, and is recommended by the NIH Guidelines. For routine clinic visits, patients were seen by one of the doctor of pharmacy investigators. Medication adjustments were done by NIH Guidelines. Objective assessment of asthma was done at each clinic visit as well as review of the PEFR patient diary, symptom frequency, and "as needed" albuterol requirement. For unusual presentations or if patients were not clinically stable, the pulmonologist investigator was always available to see patients. Other medications required by some patients included occasional antibiotics for sinusitis, histamine (H2) receptor antagonists for gastroesophageal reflux, and intranasal beclomethasone for allergic rhinitis. A low dose of sustained release theophylline (Uniphyl, Purdue-Frederick, Norwalk, CT) in the evening only was used as adjunctive therapy for nocturnal asthma only if tailored doses of inhaled beclomethasone did not eliminate nighttime or early morning symptoms. Oral beta agonists were not used in our patients. When the study was initiated, salmeterol was not available in the United States, and was not allowed until completion of the 2-year intervention.
Kim, 2011a	During the baseline evaluation, trained bilingual nurses obtained 3 BP measurements at 1-minute intervals, while demographic and psychosocial variables were measured via self-report. Participants were then assigned to the in-class to receive HBP-related education for a 6-week period. On completion of the 6-week education, participants were given a BP monitor equipped with a teletransmission system along with instructions. They were asked to measure their BP at home and transmit the BP data via phone during the following 6 weeks of the test period, during which it was made certain that all participants were able to measure and transmit their BP readings.
Kim, 2011b	At 3 months, patients were assigned to the less-intensive telephone counseling (LIC) groups. The LIC group received less-frequent, monthly counseling by a trained bilingual nurse for 12 months. Trained nurse counselors made the phone calls from a private office at a local community center. As a safety measure, nurses called the participants regardless of the counseling schedule if the transmitted BP readings were 160 ⁄ 100 mm Hg and contacted a consulting physician on the study team. When the BP readings were 180 ⁄ 110 mm Hg, nurses either called the patient immediately to ask him ⁄ her to visit a nearby emergency department or called his ⁄ her physician. Nurses followed-up with off-schedule phone calls and then with an additional call within a week for updates. During the counseling period, participants were instructed to measure their BP 3 times both on waking (AM reading) as well as before retiring to bed at night (PM reading). The participants were asked to perform both sets of triplicate measurements 2 a week. The transmission device automatically saved up to 200 BP measurements. Nevertheless, in order to facilitate timely screening of abnormal BP readings and feedback, participants were asked to send their measurements via telephone once a week. Transmitted BP data was used to generate BP reports, which showed the time and frequency of both the BP measurements and BP readings. BP reports were made available to both the participant as well as the nurse counselor throughout the 12-month counseling period.
Krishan, 1979	Details of the study design have been reported elsewhere." In brief, three noncontiguous rural Minnesota communities—Spring Valley, Owatonna, and Wabasha—participated in the program. The Spring Valley area represents a widely dispersed population that depends on several independent solo practices in this region. The town of Owatonna is served by solo and small group practices. In Wabasha, a small group practice provides medical care for a scattered farming population surrounding the rural town. Population densities for these three communities are 26, 63, and 33 persons per square mile, respectively–an indication of their rural character. As noted previously, different approaches to hypertension control were employed in the three communities. Screening for hypertension took place among all residents aged 30 to 69 years old in designated townships surrounding Spring Valley and Wabasha and among persons in a 50% random sample derived from Owatonna.

	Public education consisted of radio, television, and newspaper coverage. Special pamphlets were utilized to inform the residents of the hazard of high blood pressure, the availability of treatment, and the community program that was to be initiated. Continuing education sessions concerning hypertension were provided to all physicians in Owatonna and Wabasha with particular attention to morbidity, mortality, drug therapy, and long-term patient compliance. In Spring Valley, no special educational program was provided for community physicians. Local housewives in each community Were trained in standardized methods of interviewing and in blood pressure measurement." Korotkow phases 1 and 5 were used to establish levels of systolic and diastolic blood pressure, respectively. Questionnaires containing items such as the demographic and health profiles of the participants were pretested, and interviewers received detailed instruction. The initial screening activity was held in Wabasha over a weekend in April 1974, at a central location; after this Central screening, a house-to-house survey was undertaken in all three communities. All identified hypertensive persons were referred to the available sources of medical care. In Wabasha, an option of referral to a newly established centrally located Community Hypertension Clinic was provided. This experimental community based hypertension clinic was staffed by nurse practitioners who received intensive training in hypertension in the Hypertension Unit at the Mayo Clinic, in accordance with a protocol for initial evaluation and progressive step-wise drug therapy for hypertension. Two physicians from the Wabasha medical community agreed to undertake the primary responsibility of supervising the nurse practitioners. The overall management of hypertensive persons in this community was provided by the nurse-physician team, with the predominant role being played by the nurses in areas of adjustment of the drug regimen, and follow-up care. In summary, the interventions described above were as follows: Community Screening, Public education, Physician education, Community hypertension clinic<br><br>Referral for care from the central and household screening was recommended in persons whose levels were at or above 160 mm Hg systolic or 95 mm Hg diastolic, based on the average of the second and third readings. Patients who were already receiving treatment were advised to continue care with their local physicians (or, in Wabasha, if they preferred, at the Community Hypertension Clinic). Patients seeking care in the Community Hypertension Clinic were evaluated and placed on a management program. Drug therapy was recommended in all persons with average diastolic blood pressures of 95 mm Hg or more on two successive clinic visits. For those persons who had isolated systolic elevations or who had diastolic blood pressures in the range of 90 to 94 mm Hg, the judgment of the clinician, based on consideration of age, sex, family history, smoking habits, and co-morbidity, was considered paramount. The management program, therefore, included either drug intervention on dietary approaches including weight reduction and regulated salt intake
Chavannes, 2009	**Picasso Bocholtz study**<br>The Picasso Bocholtz study was a controlled clinical trial assessing the effects of IDM on health status in COPD patients from two comparable primary health care centers in the south of The Netherlands. Patients were followed up for two years, during which time the intervention group received an IDM program and the control group received usual care. Patients were included based on chronic respiratory complaints, postbronchodilator lung function testing, and adequate workup in case of more complex disease by a local pulmonologist, on indication by the patients' primary care physician. In the intervention setting, an integrated COPD management team was formed, including two physiotherapists, a respiratory nurse, a physician assistant, a dietician, a pharmacist, a supervising primary care physician, and a logistics manager. All team members contributed in their area of expertise to a standardized written treatment protocol, which included different elements of IDM, based on the joint American Thoracic Society/European Respiratory Society COPD standards.14 Examples included personalized physical activity training programmers, optimal medication prescribing and adherence monitoring, rapid action plans for exacerbations, and continuous self-management education.<br><br>**Kroonluchter cohort**<br>Based on the encouraging results of the Bocholtz study, the Kroonluchter integrated disease management program was implemented in a low socioeconomic status borough in Rotterdam. Since 2006, a total of 216 primary care patients with chronic respiratory complaints have been included after clinical assessment, including postbronchodilator lung function testing, confirmed eligibility according to GOLD (Global Initiative for Chronic Obstructive Lung Disease) criteria. A multidisciplinary dedicated team of primary care physicians, nurse specialists, and physiotherapists was formed and trained to establish a locally agreed collaborative protocol. Diagnostic workup in case of complex disease was provided by collaborating pulmonologists, after referral by the primary care physician. In cooperation with the patient, an individualized plan of action was designed, based on an explicitly formulated personal target, varying from "quitting smoking with guidance within six months" to "climbing a short flight of stairs without hindrance by feelings of dyspnea within six months". Based on disease burden and patient needs, an individual program was assembled, which could include self-management training and exacerbation management, an exercise training program, smoking cessation strategies, better medication use, and personalized disease education. In case of obesity or muscular depletion, referral to a dietician for dietary intervention was possible. Because of good local collaboration and arrangements for additional workup, patients could be referred to pulmonary physicians at short notice. In addition, extra attention was given to follow-up of patients after an exacerbation. Patients with an MRC dyspnea score . or patients known by their primary care physician to be

	inactive were assigned to a six-month COPD-specific training program run by specialized physiotherapists. Physical exercise training consisted of one month of individual training, followed by five months of group training. Training was focused on strength as well as endurance exercises, and was tailored to the individual abilities and limitations of the patient. Patients trained for one hour twice per week under supervision and were instructed to train one hour per week at home. After six months, there was a follow-up of one hour per week in order to sustain any effects over time.
Lawrence, 2008	The study population consisted of patients aged 18 to 65 years participating in DM programs for CVD or diabetes. Eligibility for the DM programs was determined using medical and pharmacy claims data to identify patients with chronic conditions. These patients then opted-in to the program by completing a health survey that collected self-reported health and lifestyle information. They were then stratified as high risk based on a scoring algorithm that takes into account their severity of illness as well as current knowledge of disease and self-management skills.<br><br>The LATE software (Pfizer Inc., New York, NY) consists of programmed queries run against a Microsoft Access database.<br>These queries create target patient lists based on predetermined adherence criteria. This software was integrated with BCBSSC's existing care management platform, a Webbased clinical decision support tool called InformaCare® (Pfizer Health Solutions, Santa Monica, CA), to document contacts and patient data. The approach was to integrate the LATE Program into ongoing care management processes in order to enable a timely and targeted intervention for medication adherence. The program began in November 2004 and we report results here through March 2006. To allow sufficient time for follow-up and completion of a LATE program task, only tasks initiated before December 2005 were included in this report. Five cardiovascular- and diabetes-specific classes of medications were the targeted focus of this particular initiative: lipid-lowering medications, angiotensin-converting enzyme inhibitors/angiotensin receptor blockers, beta-blockers, other hypertension classes, and oral hypoglycemic agents. Any prescription with a most recent fill date within the previous 120 days that was 60 days or more past the medication refill date was considered a "late" prescription. To operationalize the LATE Program, pharmacy claims data were obtained from BCBSSC's pharmacy benefits manager, Caremark, and uploaded on a weekly basis into InformaCare. Pharmacy claims data for both study and control group patients were then processed with the LATE software in order to identify lags in medication refills. For the purposes of this study, an episode of nonadherence was identified when a medication in any 1 of the 5 targeted drug classes was overdue for refill by at least 60 days. Prior to program implementation, the LATE software was assessed for utility using a sample of BCBSSC pharmacy claims data to ensure accuracy of the resulting reports. The study process flow is illustrated in Fig. 1. Each individual could have more than 1 episode of nonadherence identified because adherence was applied at the level of a unique prescription. That is, for each instance in which a study patient was identified as nonadherent, a DM "task" was created automatically in the InformaCare patient record. This task was an electronic reminder that prompted the care manager to address medication adherence during the next telephonic contact with the patient. Medication adherence was specifically addressed as part of the next regularly scheduled call with the patient, or a separate call was placed, as deemed appropriate by the nurse care manager. The reason for nonadherence was not captured. Once care managers completed the telephonic intervention with the patient, they designated the task "complete" in the InformaCare system. Only tasks identified as being completed in this way were included in this study.<br><br>The LATE program intervention consisted of direct telephonic interaction between care managers and patients, with a targeted focus on medication adherence. Care managers received training in how to respond to the medication nonadherence tasks appearing in InformaCare and were provided rationales and basic talking points for addressing the task with patients. Care managers also received inservice training sessions in health behavior change techniques in order to address patients' readiness to change behaviors related to medication use, and completed training in motivational interviewing and active listening techniques. They also reviewed common barriers to medication adherence and explored various resources available to overcome barriers including side effect management, mail order benefits, drug assistance programs, medication organizers, and reminder systems.
Montero, 2005	The 180 patients were randomized into 2 groups: one followed our cardiac rehabilitation program (RG) and the other, the control group (CG), received conventional treatment and served as a point of reference. The Hospital Ramón y Cajal CRP begins at 2 weeks post- discharge and consists of:<br>1. Three months supervised, individualized physical training.<br>2. Psychological program including behavior modification techniques, group therapy, and relaxation sessions.<br>3. Educational program on modifying lifestyle and controlling coronary risk factors.<br>4. Return to work counseling.<br><br>Physical training consisted of 3 1-hour sessions per week in the hospital gym. At each session, patients followed a table of physiotherapy and aerobic training on mats or an exercise bicycle. During training, heart rate was calculated individually following stress exercise treadmill tests (Bruce protocol) at the start and end of the program. The initial test served to filter enrolments and patients with ischemia and low exercise levels were excluded and indicated for coronary angiography. Patients with exercise tests without signs of ischemia had

	their target heart rate set at 75% of the maximum achieved for the first 6 weeks of training and 85% for the last 6 weeks. Supervised training was complemented by progressively increasing daily walks of £1 hour in duration, when patients tried to maintain the heart rate achieved during training. Walks were undertaken by patients individually and were unsupervised. The psychological program consisted of individualized evaluation of psychological profile using tests to evaluate anxiety (Hamilton), depression (Zung), and personality (Bortner), and Schultz method relaxation techniques practiced 2 days per week. Once a week patients underwent group therapy. Patients with greater deterioration received individualized treatment from psychologists and, if needed, from the unit psychiatrist. The educational program involved a weekly seminar for patients and immediate family to inform and advise about the illness and the need to modify coronary risk factors and lifestyle. Return to work counseling included social and vocational evaluation and recommendations on post-discharge return to work. Phase II CRP interventions were performed full- or part- time by staff of a multidisciplinary unit depending on the cardiology service and consisting of 4 cardiologists, 1 rehabilitation specialist, 2 psychologists, 1 psychiatrist, 2 nurses, 2 physiotherapists, and 1 social worker. Phase III of the program covered the rest of the patient's life. According to the discharge report, patients continued to take physical exercise 4-5 days a week, followed relaxation guidelines 2-3 times per day, and controlled risk factors. For 4 years, patients attended the hospital rehabilitation unit once a month for physical training and relaxation sessions to support them in fulfilling CRP recommendations. Following current unit norms, patients were later advised to continue taking exercise individually or in a gym following specific training norms in the discharge report. After post-AMI discharge, CG patients were advised to take standard secondary prevention measures: stop smoking, follow a low cholesterol diet, and take physical exercise, without following a structured CRP. Patients in both groups underwent periodic cardiologic outpatient clinic check-ups at 1, 3, 6, and 12 months during the first year, and once a year or according to need thereafter, up to the time of writing. To avoid differences in clinical management during follow-up, all patients were reviewed and treated by the 4 rehabilitation unit cardiologists. Medical treatment during follow-up was that considered adequate by the cardiologists responsible for clinical control of the patients.
Mildestvedt, 2008	In total, 266 patients enrolled on a 4-week CR programme at the Krokeide Rehabilitation Centre were invited to participate in the study. Approximately half of the patients were inpatients. The programme provided a multifactor focus on lifestyle changes. They participated in daily exercise groups and received information about the benefits of exercise training. To detect a difference in change of exercise mean score of 15% with 90% power at a 5% significance level, we needed 68 participants in each group. The estimates of standard deviations are based on results from a former study. Patients attending the rehabilitation Centre were recruited from hospitals and primary care physicians in the West Norway health region. Not all patients who have experienced a coronary event are offered cardiac rehabilitation. Patients were recruited both from secondary and primary healthcare settings and were accepted within few weeks of waiting if suitable for the intensive rehabilitation programme. Patients could attend 4 to 6 weeks after a myocardial infarction or 6 to 8 weeks after an aortocoronary bypass operation. Over a 2-year period ending in August 2002, 217 patients agreed to participate in the study. They were randomly assigned to one of two groups: the control group received the standard rehabilitation treatment, which included daily exercise training; the intervention group received the standard treatment plus an additional individualized self-efficacy and autonomy supportive intervention. Forty-one patients were lost to follow-up or left the rehabilitation programme for various reasons, leaving 176 patients eligible for 2-year follow-up analyses. Their data are presented in Table I. The 41 participants not answering the questionnaires or attending less than 2 weeks of the rehabilitation course were excluded. Intention to treat analyses was performed with replacing the lost variable at 24 months with the lowest score at inclusion or 6 months' follow up, a worst case scenario. The intervention group received two individual sessions during the rehabilitation stay and two follow-up telephone calls at 6 and 24 months, focusing on personally selected goals. The followup telephone consultations took place approximately at the same time as the participants received the postal questionnaires. The two sessions of individual counselling were of approximately one hour duration each. They were carried out by one of three different members of the staff; a psychologist, a nurse and a social worker. The same staff provided the standard treatment rehabilitation. They all had several years of practice as rehabilitation counsellors. We based our method on a structured intervention supported by written material, but where the content in this structure was provided by the patient in a respectful dialogue with the clinician. We aimed at helping the patient structure and focus his or her tasks by providing choice and respect for the patients' autonomy and responsibility. The intervention was based on cognitive behavior techniques and autonomy support from SDT. The overall structure of the consultations started with an introduction; followed by a problem solving phase; and a closing session with a plan for home work and appointments of follow-ups. During this intervention the patients were challenged in specifying their three most urgent lifestyle aims and the possible means necessary to reach their objectives. Increasing physical activities was one of nine possible lifestyle achievements to choose from. Exercise was stimulated by daily supervised training sessions. The group-based setting aimed to increase personal competence, both by own experience and by observing other patients performing relatively intensive cardiac fitness training. The training programmes try to motivate patients to overcome fears of increasing cardiac strain. In addition, group-based, didactic counselling was given, focusing on the rationale for exercise in the prevention of cardiac disease.
Morisky, 1983	The exit interview, conducted immediately following the patient's encounter with his or her medical provider, was an individualized 5-10 minute counseling session explaining and reinforcing the instructions of the practitioner whom the patient had just seen, and adapting the regimen to the patient's individual schedule, cueing behaviors (e.g., medication taking) to daily activities (e.g., toothbrushing). All 200 patients randomly assigned to this intervention received it. The second intervention consisted of an instructional session with an adult whom the patient identified as having the most frequent contact at home, usually a spouse. The education in this encounter discussed how the household member could help the

	patient adhere to the regimen and follow-up care. A total of 160 of the 200 patients randomly assigned to this intervention were interviewed. The third intervention was a series of three, one-hour group sessions led by a social worker which patients were invited to attend. The purpose was to provide group support, to strengthen the self-confidence of patients through discussions centering on hypertension management and compliance. The sessions used a broad range of action-related procedures (e.g., role-playing, behavioral rehearsal, problem clarification, cognitive restructuring). A total of 96 of the 200 patients randomly assigned to the small group intervention attended at least one session
Olson, 2009	Patients randomized to CPCRS care continued to receive the standard care delivered by the CPCRS for all CAD patients in the program. This care consisted of clinical pharmacy specialists telephoning patients to review the results of their annual FLP, blood pressure measurements, and medications and adherence; counseling on diet and exercise regimens; and making medication adjustments to maintain treatment goals. The specialists ordered follow-up laboratory tests through the electronic medical record. Additionally, all patients were mailed letters informing them of their results, were scheduled for a follow-up FLP, and received laboratory reminder letters. Each patient contact was documented in the electronic medical record for other healthcare providers to review and was estimated to take the clinical pharmacy specialist approximately 15 minutes. Patients randomized to usual care were discharged from the CPCRS at the time of study enrollment (September 1, 2005), and an FLP was ordered for 1 year in the future with results to be returned electronically to each patient's primary care physician. It was the responsibility of the physician to address the results as they became available and order follow-up laboratory tests, as needed, during the study period. Patients in the usual care group received laboratory reminder letters that were generated electronically using the HealthTrac database. However, unlike the CPCRS care group, patients in the usual care group received no contact or counseling by the CPCRS specialists or letters informing them of their FLP or blood pressure results. Other than the aforementioned reminder letters, physicians received no additional clinical assistance from CPCRS specialists with respect to the usual care patients. In rare instances, physicians were able to contact the CPCRS with specific questions regarding patients with significant medication side effects. Physicians did, however, have access to regional guidelines for the treatment of CAD risk factors.
Perk, 1989	**Continued long-term cardiac rehabilitation program (LTP):**<br>A two year comprehensive cardiac rehabilitation program, consisting of health education, follow-up at a post MI clinic and physical training in props is provided for myocardial infarction (MI) patients. We designed a long-term programme for those patients who wished to continue: once a month a 45 min group training session consisting of jogging, calisthenics, stretching and relaxation is offered at the department of physiotherapy. The sessions are held after working hours in order not to interfere with the working hours of the participants or with the daily activities at the department of physiotherapy. Two groups of patients participate with approx. 10-15 patients in each group. Necessary safety and resuscitation equipment is available. Occasionally physicians from the rehabilitation team join the training which gives a good opportunity for informal health education, encouraging the patients to keep up and active way of living and to abstain from smoking. The patients are regularly informed that training once a month does not substitute the individual training at home, but that it is a valuable way to keeping up their level of fitness. Patients who wish to participate may continue to do so, as long as they wish. The programme has no time limit. By using the available training facilities the programme operates on a low cost base. The salary for the supervising physiotherapist is paid by the Swedish Heart Patients Association. In December 1985 we performed a controlled study in order to evaluate the long term programme. The study group consisted of 20 patients participating regularly in long-term cardiac rehabilitation program (LTP) (defined as attending at least 3 out of 5 training sessions during a 5-month period).
Phillips, 2005	From 1999 through 2002, all residents (including all intervention arms) and faculty members were given yearly orientations about the trial and yearly lectures about management of type 2 diabetes; the importance of early provider action was emphasized. Each year, all residents were also given pocket cards that reiterated treatment goals and therapeutic thresholds and sent letters that provided feedback about the completeness of their visit notes.<br><br>**Computerized reminders.** Decision support included both a flowsheet to show sequential laboratory values, weight, blood pressure, and use of medications and recommendations for treatment and was provided for 85% of visits of patients in the Reminders groups; residents in this group acknowledged in anonymous questionnaires that they had received the reminders. The recommendations for management of hyperglycemia, hypertension, and dyslipidemia were based on IPCAAD algorithms developed by the endocrinologist and generalist leaders of the study and confirmed for clinical acceptability; use of the hyperglycemia algorithm in the diabetes clinic improved attainment of A1C goals (A1C <7%) . The algorithms took into account each patient's medications (as prescribed at the previous visit) and recent laboratory/clinical values (≥1 week before the visit) and recommended changes in dosage of current medications and/or new medications. Such individualized features are thought to make it most likely that providers will respond appropriately.<br><br>**Feedback on performance.** Feedback sessions between one of the endocrinologists and a resident were ~5 min in duration and scheduled every 2 weeks; missed sessions were made up later, and feedback was provided overall for 97% of scheduled sessions. The sessions specifically avoided consultative advice on management. Instead, feedback was based on IPCAAD report cards that showed individual provider actions or outcomes of the patients seen by that provider but did not identify specific patients. Emphasis was placed on

	achieving ADA goals and on acting when values were abnormal during visits, i.e., random capillary glucose > 150 mg/dl or systolic blood pressure (sBP) > 130 mmHg. We have found such glucose values to be highly predictive of A1C > 7% and intensification of therapy based on such values to be associated with very little risk of severe hypoglycemia. (Home glucose monitoring data are frequently not available; only 24% of visits in one Grady primary care site had data brought in.) The sessions were designed to be interactive and were scripted to elicit responses from the residents. Such "active" features are thought to be important in adult learning and should help change provider behavior.
Pill, 1998	**The controlled trial**<br>Over a 3-year period, an intervention team (GP, research nurse and clinical psychologist) was responsible for recruitment and training, and an evaluation team (sociologist and psychologist) was responsible for data collection and analysis. The unit of randomization was the practice, and the population was 33 South Glamorgan practices that had been committed for at least 2 years to an annual peer review clinical audit of diabetic care. Recruitment was carried out sequentially over 6 months by the GP member of the intervention team, the order of approach being determined by a random number list. Four of the 33 eligible practices declined to participate for organizational reasons (i.e. changing staff, new partners, previous research commitments). After recruitment, each practice was allocated by block randomization independently to each arm of the trial. All practices were asked to recruit at least 12 patients who met the following criteria: aged 18–70 years; diagnosed > 1 year; glycosylated haemoglobin >9% at last reading; (normal reference range 5.7–8.0); life expectancy of at least 3 years. The sample size was determined by the estimate that 100 patients would need to be randomized into each arm of the trial to detect a difference of 1% in mean Glyco-Hb level, with 80% power at the 0.05 level. All practices were given a recruiting pack with appropriate consent and information forms. The control practices were also provided with the standard British Diabetic Association leaflets to use with their patients and encouraged in the task of recruitment by bimonthly newsletters. Training for experimental practices was delivered at the surgery to the GP(s) and the practice nurse(s) most closely identified with the delivery of diabetic care. Simple visual aids were designed to assist the clinician in encouraging active patient participation. The core message was that the patient should be allowed to air their personal concerns about their condition, to select which particular topic they felt most relevant for discussion (taking into account their readiness to change) and if appropriate, to set a specific target for themselves. Following adult learning principles, training was tailored to the needs of the individual practices, with the amount of contact being determined by the clinicians. However, all experimental practices had at least two training sessions (i.e. 3 hours), comprising discussion, demonstration of the technology and often roleplay. Continuing support was provided by the research nurse, mostly visiting the practice nurses, newsletters every 3–4 months and two group meetings during the course of the study. - Patient data were collected in the subject's home by a psychologist who was blind to their experimental status. A questionnaire was left for self-completion, although assistance was provided if requested. The same data were collected again approximately 18 months later. Established and previously validated measures included the anglicized short-form SF-36 questionnaire," which was used to provide information on health status, and diabetes-specific measures of wellbeing and satisfaction with treatment." Seven new scales, based on the theoretical assumptions underpinning the intervention, were designed to test changes in specific attitudes and expectancies. These were piloted and tested for reliability in a separate sample before being included in the present study." Routinely collected clinical audit data were abstracted at recruitment, and approximately 9 and 18 months later. These included Glyco-Hb, BMI, blood pressure, clinical complications, medication use, presence of other conditions and lifestyle behaviours, e.g. smoking and alcohol use, and attendance rates at the surgery.<br><br>**The process study**<br>Clinicians' interest in the intervention was monitored by comments during informal contacts throughout the study, responses to follow up visits by the research nurse, feedback at the group meetings and a postal questionnaire on their use of the method (see Figure 1). Audiotaped consultations were analysed in order to assess clinician competence to apply the method 8 or 9 months after training. The experimental clinicians were asked to submit a recording which "demonstrated the use of the method they had been taught", one that they themselves felt was a fair reflection of what they could do. Control clinicians were requested to record "a typical interview". All tapes were numbered, transcribed and coded blind by the evaluation team. The coding framework, refined over several months to ensure reliability and validity, focused on whether the patient or doctor initiated the discussion of health behaviour, affirmed the maintenance of existing appropriate behaviour and initiated any suggestion of change. If change was raised, attention focused on whether the topic was pursued, who it was pursued by, and whether a specific target was agreed upon. Use of the intervention over time was assessed by telephone interview at the end of the study. Experimental clinicians were asked how they would describe the study to a colleague who knew nothing about it, to comment on any implementation problems and to say how much they were using it in practice.
Radziewicz, 2009	To facilitate the understanding of the variety of fidelity tools used in our RCT, we overview the components of the intervention. In the CCS intervention study, patients with advanced cancer and FCGs are screened for eligibility. If eligible, the patient/FCG is asked for his/her consent, and the patient is screened for distress, as defined by the National Comprehensive Cancer Network Practice Guidelines for Distress.16 The patient/FCG dyad is randomized into either the usual care or the intervention arm of the study. Research assistants, who were blinded to which group the patient/FCG dyads are randomized, interview them at baseline about quality of healthcare and quality of life. Outcomes are assessed through structured interviews conducted with patients and FCGs by a range of measurement tools and through patient medical chart review once at 3 months after enrollment and

	with surviving patients at 6, 12, and 18 months after enrollment. The patients and FCGs randomized into the intervention are randomly assigned to a primary CCSP, a nurse with mental health training serving as a coping and communication coach, for the duration of their participation. Coping and communication support practitioners are provided screening information on the patient's level of distress and are informed of the patients' and FCGs' health information processing style, for example, monitoring and blunting and level of depression and anxiety at the outset of the intervention. The CCSP arranges an initial face-to-face meeting with the patient and FCG to establish rapport, review which services can be provided to facilitate CCS, identify problems and issues, and determine potentially helpful intervention skills. The CCSP functions include facilitation of patient/FCG goals, assistance with resolution of problems, assistance with navigating the healthcare system, and support of the patient, as requested, in communicating with physicians. The CCSP is not part of the healthcare team and therefore functions without access to the medical record. Patients and FCGs have 24/7 direct telephone access to their primary or another covering CCSP as needed. At the initial visit and each subsequent contact, the patient and FCG determine their future contact preferences including the mode (telephone, e mail, or clinic visit) and frequency (if contact is desired) and who initiates the next contact. Patients remain in the intervention until death, and the FCG continues to have access to the intervention for up to 1 year after the death to ease bereavement. Patients are invited to use the CCSP as much or as little as desired, but an initial follow-up contact is made within 2 weeks of the initial meeting to ensure that the patient and FCG have had time to consider their preferences for future contact and their needs and goals. A patient with high distress (distress rating of *Q*4 using the National Comprehensive Cancer Network distress screening tool) is contacted minimally on a monthly basis by the CCSP unless the participant has expressed preference for less frequent contact. Patient and FCG preferences and engagement in the intervention were documented over time.
Reichard, 1996	The treatment programmes have been described, and consisted primarily of education and continuous tutoring. After 7.5 years' study the ICT patients had regular visits every 4 months. Many of the ST patients had frequent contact with the physician after the 7.5-year period, and were then given advice on how to further improve their treatment. The goal was to reduce the HbA1c to 7%, and the patients were informed that the study so far had shown that this was the level of HbA1c needed in order to halt the long-term complications. The intensification of treatment in the ST group thus consisted of information, education, close office contacts, home blood glucose tests and for most of the patients three or more injections daily. Two patients in the ST group but none in the ICT group, also began to use subcutaneous insulin infusion treatment with a pump.
Rothschild, 2012	**Community health worker intervention**<br>The MATCH intervention employed CHWs as culturally competent peer interventionists to enhance diabetes self-efficacy and increase rates of diabetes self-management behaviors. MATCH CHWs were Mexican-Americans, for whom Spanish was their first language, recruited from the same neighborhoods as study participants. CHW training and evaluation protocols have been described previously. The CHWs themselves did not have diagnosed diabetes. The MATCH curriculum taught seven core diabetes self-management behaviors, developed from the curriculum content recommended by the American Academy of Diabetes Educators, the "AADE-7". In order to help participants overcome barriers and begin to successfully implement these behaviors, the CHWs also coached participants in five general skills: brainstorming and problem-solving; using a journal or written record; modifying the home environment to support behavior change; seeking social support from family or friends; and stress management. Training was based on principles of social cognitive theory positing that individuals will make behavior changes only if they believe that they are capable of organizing and executing the specific behaviors. By facilitating problem-solving, providing performance feedback, and coaching participants through repeated efforts at implementing diabetes self-management behaviors, the CHWs were to help participants gain self-efficacy and make sustained changes. The curriculum was delivered in 36 individual visits, usually in the home of the participants, over a two year period. Individual home visits were selected rather than a group intervention in order to promote behavior changes in the milieu in which those behaviors most often take place. Visits were scheduled twice monthly during the first year (24 visits), and once a month in the second year (12 visits). This number of visits was selected because skill mastery occurs gradually over numerous attempts, and in stages going from acquisition to generalization and ultimately maintenance. Initial visits focused on building a relationship with the participant, with particular emphasis on identifying the participant's concerns and mastery of basic diabetes knowledge. These visits also introduced self-management techniques and an overview of the curriculum. By the fourth visit, CHWs began to work with one or two specific diabetes self-management issues at a time. Although core content was specified by study protocol, the specific training sequence was not; sequence of content was individualized by the CHW in response to challenges that the participant voiced at the time of the visit. The CHW then coached the participant in using self-management skills to help them successfully implement diabetes self-care behaviors. As the first year progressed, it was anticipated that participants would be better able to apply general skills to improving diabetes self-care. Repeated visits covering the same diabetes self-management behaviors were scheduled during the second year to provide participants with an opportunity to further increase self-efficacy and foster long-term maintenance of the targeted diabetes behaviors. Intervention fidelity is of critical importance in a behavioral clinical trial. In MATCH, this was ensured by requiring the CHWs to complete a documentation worksheet for each intervention visit. CHWs documented the diabetes behavior that was the primary focus of the visit, and the self-management strategies that were taught. All visits were audiotaped with the consent of the participants, and tapes reviewed by the project psychologist (CL) to ensure that the documentation worksheets were accurate and to provide on-going feedback to CHWs regarding intervention delivery and consistent application of behavioral self-management training techniques. Monthly control reports ensured that each participant received all of the components of the MATCH intervention.

	**Control condition**<br>Participants randomized to the control condition received a bilingual newsletter called "DiabetesAction". Thirty-six newsletters were mailed to control participants on the same schedule as the CHW intervention. Newsletters covered the seven diabetes self-management behaviors, and provided tips to promote problem-solving, social support, environmental restructuring, self-monitoring, and stress management.
Rowley, 2000	**Intervention among high-risk overweight and diabetic people:**<br>Interventions included formal and informal education sessions, regular physical activity groups and dietary changes such as cutting fat from meat before cooking, reducing intake of refined carbohydrate (sugar *per se* and carbonated beverages) and increasing consumption of fresh vegetables and fruit. Cooking classes and store tours were organised to help people identify healthy food choices. Physical activity was promoted with regular hunting trips, participation in sport (basketball or football, 2- 3 sessions per week) and regular walking groups (3-4 times per week, one hour per session). Several family groups began regular walking independently of the program. Informal education sessions about diabetes were an important component of the program. Weekly body weight and blood glucose checks were available for those who requested them.<br><br>**Intervention in the wider community:**<br>After two years, a growing awareness in the wider community of the importance of diet and physical activity for preventing chronic disease led the community to rename the program Looma Healthy Lifestyle. There was a strong emphasis on dissemination of messages about diet and physical activity to family members by those persons taking part in the high-risk intervention program. Health promotion activities, arising from the initial intervention and in which all community members were invited to participate, were undertaken to initiate normative change and enabling conditions. Details of initiatives are given below. Throughout the course of the intervention, technical advice, data analysis and feedback of results and advocacy to health and funding bodies was provided by researchers.<br><br>*Looma Diabetes Program/Looma Healthy Lifestyle*<br>• Appoint community members as diabetes workers.<br>• Looma Healthy Lifestyle Regular hunting trips for program participants.<br>• Nutrition education for diabetic people.<br>• Healthy cooking classes.<br>• Store tours to identify healthy food choices.<br><br>*Community Council:*<br>• Appointment of community member as store manager with mandate to improve food quality.<br>• Non-smoking policy in public buildings.<br>• Provision of vehicle for Looma Diabetes Program.<br>• Appointment of sport and recreation officer.<br>• Health promotion (sporting/art competitions).<br><br>*Community Store:*<br>• Improved quality and quantity of fresh produce.<br>• Availability of wholemeal bread and flour.<br>• Replacement of butter with margarine.<br><br>*Community Members:*<br>• Regular family walking groups.<br>• Activation of sporting teams.

Skinner, 2000	At each site, a steering committee was assembled from the STAES membership to plan and "host" the 3 core, half-day Learn, Share & Live education sessions. Sessions were led by health care professionals andwere designed to promote understanding about breast cancer and screening as well as to promote screening among participants' peers. As noted in Table 1, 32 and 47 women, respectively, attended at least 1 core education session at the index and replication sites. Following core sessions, women who had attended Learn, Share&Live chose from a menu of optional activities for disseminating among the wider STAES membership what they had learned through the program (for a detailed description of core sessions and follow-up activities, see Skinner et al.
Stroebel, 2000	Key implementation goals and measurements were based on an evidence-based hypertension treatment guideline developed by the Institute for Clinical System Improvement (ICSI; Minneapolis). ICSI, of which the Mayo Clinic is a member, is a nonprofit quality improvement (QI) organization that supports the development of evidence-based clinical guidelines for use within the member medical groups. The hypertension treatment guideline is consistent with sixth Joint National Committee on Detection, Evaluation and Treatment of High Blood Pressure (JNC VI) recommendations for BP management.<br><br>A guideline implementation team (GIT), led by two of the authors [R.J.S., S.K.H.], planned and oversaw the intervention. The guideline was provided to all physicians in the practice during division meetings and educational sessions and was available to physicians via the Mayo Clinic intranet. Broad agreement with the guideline's goals and recommendations was evident during the meetings. After the guideline recommendations were provided to the staff, the GIT developed an implementation plan to incorporate the recommendations into practice. The implementation plan involved a ream approach to hypertension management, with the LPN, RN, and MD all participating in a cooperative effort. In this model of care, patients who came to the clinic for an office visit were roomed by a LPN and had a standardized BP taken. If the BP was elevated (> 140 mm Hg/> 90 mm Hg), it was re-measured after five minutes. The LPN automatically referred patients with two elevated readings for a follow-up RN visit in one to two weeks. The appointment could be canceled or altered by the MD during the office visit if he or she deemed an alternative plan more appropriate. For example, the MD may have changed the medication during that visit and scheduled the patient for a return MD visit versus seeing the RN. If, however, the MD did not notice the elevated BP or did not have time to address the problem, the RN follow-up appointment would occur (Figure 1, p 626). The follow-up RN visit was 15-30 minutes long. Patients were educated on cardiac risk factors, lifestyle modification, and medication compliance. Patients were encouraged to set goals for improvement in these areas. Assessment included discussion of medication side effects and repeat standardized BP checks. All management decisions were reviewed with the MD in team meetings, which occurred at the end of a half day or before the clinic started the following day. The MD could decide to increase dosage, add medication, or observe the patient's progress. The RN would then schedule appropriate MD or RN follow-up visits until the BP was in control. The RN would phone the patient with the plan and document the encounter in the medical record. (The RN visit is a reimbursable service. For example, the Medicare coverage for a typical RN follow-up visit is $17.15.) Usual care in the division for a typical office visit included a standardized BP taken by the LPN or clinical assistant at the rime the patient was roomed. The BP \Vas then recorded in the chart or on a dictation template for review by the MD. Responsibility for recognition of uncontrolled hypertension and establishment of an appropriate follow-up plan rested entirely with the MD. Table 2 (p 627) summarizes the key differences between usual care and the interventions in the new model of care. Development of the ne\V 1nodel began in June 1997. In December 1997 five MD/RN/LPN teams began piloting the new program. The remainder of the division continued its usual practice. Since the initial results were quite encouraging, the model was expanded to ten additional teams in October 1998. The GIT evaluated implementation of the pilot program using small tests of change, monitoring the effectiveness of each critical step in the process. It reviewed a day of charts from the pilot teams to determine the percentage of uncontrolled hypertensive patients referred for follow-up. It also intermittently monitored the percentage of patients who kept follow-up appointments. These small tests of change facilitated prompt feedback to the pilot teams. The GIT exposed the other physicians in the division to the hypertension guideline's goals and recommendations, but did not involve these physicians in the practice redesign.
Svetkey, 2009	The Physician Intervention (MD-I) lasted 18 months and consisted of three elements:<br>1. Two training modules were provided on-line. The first module addressed the Seventh Joint National Committee on Prevention, Detection, Evaluation and Treatment of High Blood Pressure (JNC-7) guidelines and the second addressed lifestyle modification for BP control. Each module required approximately 45 minutes, included a quiz that gave immediate feedback, and provided Continuing Medical Education credit through Duke University. Participating physicians completed the modules within two weeks of randomization and before the patient intervention began.<br><br>2. Each physician in MD-I received an evaluation and treatment algorithm that summarized, on a color-coded, pocket-size laminated card, the major JNC-7 guidelines, including lifestyle guidelines and a decision tree.<br><br>3. The quality improvement (QI) procedure assessed clinical performance measures (CPMs) and provided quarterly feedback to physicians on adherence to JNC-7

	guidelines. At each MD-I site, participating physicians completed a CPM data form every time a HIP patient (in either Pt-I or Pt-C) had a clinic visit. The form recorded patient demographics, comorbidity, prior and current BP measurements, and actions taken during the visit. In addition, one day each month, MDs completed a CPM form on all adult patients treated during that day, whether or not the patient was a HIP participant, recording the same data as for study participants, but without patient identifiers.<br><br>These data were converted into personalized quarterly feedback reports that indicated: a) the proportion of hypertensive patients in the practice with adequately controlled BP, the change in that proportion over the course of the study, and comparison to the other participating MDs; b) the proportion of patients with diabetes or chronic kidney disease (CKD) who were at JNC-7 goal BP; c) the proportion of patients prescribed specific classes of medication based on JNC-7 guidelines; and d) the proportion who received lifestyle modification counseling based on physician self-report. The MD Control condition (MD-C) constituted "usual care". There was no attempt to change or monitor procedures already in place for quality improvement and physician education with regard to BP control. No performance data were collected from these physicians, and no performance feedback was given. The Patient Intervention (Pt-I) consisted of 20 weekly group sessions (n = 10–15 patients/ group) over approximately 6 months. All intervention sessions occurred at or near the patients' primary care clinic. The behavior goals of Pt-I included weight loss if overweight, the Dietary Approaches to Stop Hypertension (DASH) dietary pattern, increased moderate to vigorous physical activity, reduced sodium intake, and moderation of alcohol intake. In addition, the intervention promoted adherence to antihypertensive medication regimen. Pt-I was based on key theoretical constructs developed to guide health behavior change efforts, and on practical applications from previous trials. The intervention was designed to promote frequent self-monitoring, feedback, goal-setting, and social support, and used Motivational Interviewing techniques. Pt-I was conducted by two experienced behavioral interventionists who were trained and certified to deliver a group intervention focusing on diet and exercise and to use Motivational Interviewing techniques. They were assisted by community health advisors (CHAs). Two CHAs assisted with group sessions at each clinic and contacted participants who missed a session. After the initial 6 month intensive intervention, CHAs contacted participants by phone each month for 1 year to offer brief lifestyle counseling. The Patient Control condition (Pt-C) constituted usual care, comprising an individual visit with an interventionist to receive advice and written materials on lifestyle modification for BP control consistent with JNC-7 guidelines.
Tamone, 2012	An educational and phone call follow-up program started in April 2008 in the San Giovanni Battista Hospital of Torino and in other hospitals in the northwest of Italy to help patients during therapy with daily subcutaneous injections of either teriparatide or PTH(1–84). Women and men, examined in the previously reported hospitals, who presented the criteria for prescription of teriparatide or PTH(1–84) were eligible for inclusion in the study. Patients with contraindications to teriparatide or PTH(1–84), without indication for treatment, without a phone line at home, or not consenting for personal reasons were not included in the study. All consenting patients who started teriparatide or PTH(1–84) from April 2008 to April 2010 were enrolled in the educational and follow-up program (intervention group). At the beginning of the treatment, nurses explained the injection technique and trained patients on self-injection. Every 2 months nurses gave new drug pens to the patients; this guaranteed the surveillance of compliance. Patients received one phone call per week during the first month, then one phone call per month and per 3 months during the following 5 and 12 months, respectively. During each call, nurses helped patients to resolve any possible issue, scheduled the next visit, and, if applicable, collected adverse events information, dates, and reasons for treatment discontinuation. Patients also had the opportunity to call for possible questions or problems between the regularly scheduled phone interviews. All data were collected using a standard form. A physician visit was scheduled by nurses at 6, 12, and 18 months of treatment; in every visit, a physician evaluated the results from blood tests, discussed any possible issues with the patient, and, where applicable, renewed the therapeutic plan as requested by Italian regulations. We compared the intervention group with a historical cohort of patients, examined in the San Giovanni Battista Hospital of Torino or in the other hospitals in the northwest of Italy, who started therapy from November 2004 to March 2008 and who did not participate in any follow-up program (nonintervention group). Patients in the nonintervention group were not initially trained on drug self-injection, and every 6 months they would voluntarily submit to the visit for therapy plan renewal; the nurses did not book the visit. Every 2 months they had to come to the center to take the drug pen. In order to collect data on the causes and timing of discontinuation in the nonintervention group, we called the patients retrospectively. The choice to prescribe teriparatide or PTH(1–84) was a personal decision of the physician; the study protocol does not include any recommendation. In males and in patients taking steroids, teriparatide was prescribed in accordance with the European Medicines Agency and locale reimbursement criteria. All patients were supplemented with calcium and vitamin D.
van Wetering, 2009	**INTERCOM program**<br>The intervention consisted of an intensive four-month, standardized, supervised rehabilitation phase and a 20-month active maintenance phase. The program was designed to improve and subsequently maintain exercise capacity, to promote self-management skills and improve knowledge on COPD. Nutritional intervention and smoking cessation support were provided upon indication. The program was offered by local physiotherapists and dieticians in the proximity of the patient's home and by respiratory nurses from the hospital. Local care-givers were supervised by colleagues from the hospital. During the first four months, the patients visited the physiotherapists twice a week (30 min. per visit) for intensive exercise training consisting of endurance training (cycling and walking) and four specific exercises for upper and lower extremities to improve both strength and endurance without

	the use of special equipment. Patients were instructed to perform the same exercises twice a day during 30 minutes in their home environment in addition to walking and cycling outside. Furthermore all patients participated in an individualized education program that was structured using a patient education book. All smokers were assigned to the respiratory nurse for standardized smoking cessation counseling according to the Minimal Intervention Strategy for Lung patients. Nutritionally depleted patients received scheduled counseling (4 visits) by a dietician and nutritional supplements (Respifor®, Nutricia, Netherlands). During the 20-months active maintenance phase, patients visited the physiotherapist once a month to monitor exercise capacity and adherence to the training and to provide encouragement to continue the exercise training at home. After a patient had experienced an exacerbation, he/she was allowed to start six extra training sessions in three weeks at the physiotherapy practice. Nutritionally depleted patients visited the dietician four times in the maintenance phase i.e. after 6, 9, 12 and 24 months. The visits to the respiratory nurse were scheduled upon indication or request. The Usual Care group received pharmacotherapy according to accepted guidelines, a short smoking cessation advice by their chest physician, and if they were nutritionally depleted, a recommendation by their respiratory physician to eat more.
Weiss, 1984	**Care plan development.**<br>The assessment information for each person is summarized and presented at an interdisciplinary case conference. The interdisciplinary team consists of a nurse, social worker, physician, and other health professionals where appropriate (psychologist, physical therapist, occupational therapist, speech therapist). These professionals develop an individually tailored six-month plan of care for each participant that includes: a priority list of needs, the appropriate services, providers, locations, start date and the number of units of service recommended. Modifications and adjustments are included for the demonstration participants throughout the implementation of the care plan.<br><br>**Service coordination**<br>The implementation of the plan begins with the actual negotiation and participation of the client. The Project OPEN Service Coordinator contacts the designated service provider(s) and arranges for service delivery, i.e., the beginning date and the total amount of service to be delivered. Once the service delivery begins, the Service Coordinator continually monitors the client's condition as well as the quantity and quality of services received. This process includes consulting with the various providers to share information, to provide continuity, and to assure appropriate, quality care.<br><br>Reimbursement for services provided under contract (i.e., consortium members and other contracted services) is made on the basis of negotiated prospective rates. Other sources of funding include private insurance, Older Americans Act money, United Way and other philanthropic monies, and individual client contributions. Even though Project OPEN is not able to pay for all services rendered to its participants, it does include tracking of the actual costs of all services rendered, discussed below under "Research and Evaluation.” Since one of the primary goals of Project OPEN is to provide cost effective services, several cost containment measures are used. Perhaps the most important approach is the Service Coordinators' actual functions of assessment, care plan development, and service authorization, which all contribute to cost containment. As a broker, the Service Coordinator negotiates with the providers for the appropriate amount of services, and then monitors the cost and frequency of services used. In addition, the Service Coordinator acts as an advocate and educator in facilitating a heightened awareness among the clients regarding service costs, bills, and third party statements. Through Project OPEN'S negotiated contracts with the consortium agencies and other providers, a reduction in service costs occurs. Since the project contracts for services and refers a greater volume of people to the contracting agencies, agencies compete for the project's business. The results are lower rates. Cost saving measures occur among private physicians as well, by encouraging the practitioners to accept Medicare assignment. All waiver reimbursement occurs on an individual needs basis and not on a third party regional paper review, or diagnosis basis. The result is an increased awareness of costs and cost containment decisions. In short, cost containment is maintained through Project OPEN'S organizational structure and the design of the reimbursement system.
Wisse, 2010	Subjects were randomly assigned to either the intervention (n = 38) or control (n = 36) group. All physicians and investigators, except for the physical therapist, were blinded to subject randomization. The intervention group was stimulated to increase daily physical activity through regular, structured, and personalized exercise prescription by a physical therapist over the 2-y intervention period. The program was designed as a more intense and extended version of the PACE project (Physician-based Assessment and Counseling for Exercise). Subjects in the intervention group received two 1 h intake consults with a physical therapist. The therapist prescribed a personalized exercise intervention program based on the participants' baseline exercise pattern, medical condition, exercise tolerance, physical change motivation score and individual patient preferences. The program started with the prescription of simple tasks to increase habitual physical activity like a daily walk, climbing stairs or cycling. The goal was to achieve a physical activity level in which participants would exercise on a medium intense level for more than 160min per wk, divided over at least 3 sessions per wk. Two and 6 wks following the initial intakes, subjects received a 15min telephone call from the physical therapist. During 2-y of follow-up, subjects visited the physical therapist for a 30min consult alternated with a 15min telephone call every 6 wks. During all contacts, subjects were instructed and encouraged to achieve or maintain the intended activity prescription. The control group received no further instructions than the

	usual outpatient clinical care. Clinical measurements were performed at baseline and after 1 and 2 y of intervention. Physical activity status was assessed based on the Tecumseh/Minnesota scale. Quality of life (W-BQ12 scale), medication use, body weight, blood pressure and prevalence of diabetic complications were assessed. Fasting blood samples were collected to determine HbA1c, glucose, lipid levels and creatinin concentrations. A morning urine sample was collected to assess microalbuminuria.
Xian, 2010	Details of the GWTG Program have been previously published. In brief, the AHA launched the GWTG initiative focused on the redesign of hospital systems of care to improve the quality of care of patients with CAD, stroke, and heart failure. The GWTG-CAD program includes learning sessions, didactic sessions, best practice sharing, interactive workshops, post-meeting follow-up, and a Web-based patient management tool (Outcome, Cambridge, MA). This Web-based tool provides the opportunity for concurrent data collection, ongoing real-time feedback of hospital data, and clinical decision support to enable rapid cycle improvement. As an incentive, GWTG-CAD rewards hospitals using a performance recognition program. The program began in 2000. The length of participation of each hospital depended on the time it entered the program. Data collected include patient demographics, medical history, symptoms on arrival, in-hospital treatment and events, discharge treatment and counseling, and patient disposition. Participating institutions were instructed to submit consecutive eligible patients to the GWTG database using case ascertainment techniques similar to the Joint Commission. Outcome Sciences is the data collection and coordination center for GWTG programs. The Duke Clinical Research Institute, Durham, NC, serves as the data analysis center and has an agreement to analyze the aggregate deidentified data for research purposes.<br><br>To receive incentive payments established by Section 501 (b) of the Medicare Prescription Drug, Improvement and Modernization Act of 2003, eligible acute care hospitals in the United States began to routinely report quality performance data for certain clinical conditions including acute myocardial infarction (AMI), heart failure, and pneumonia in 2004.
