# Supplementary material for: Sustainability of knowledge translation interventions in healthcare decision-making: a scoping review
Source: Implement Sci. 2016 Apr 21;11:55. doi: 10.1186/s13012-016-0421-7 (PMC4839064; doi:10.1186/s13012-016-0421-7)
Supplement: Supplementary file 8 — Outcomes. (PDF 115 kb) [file 13012_2016_421_MOESM8_ESM.pdf]

Appendix 8. Outcomes

First Author, Year	Outcome Themes [Combined]	Outcome Levels [Combined]
Allen-Ramey, 2002	Disease severity, Pulmonary function, Knowledge, Health care utilization, Compliance, Employment	Patient level, Systems level
Ambrosio, 1983	Health care utilization, Blood pressure	Patient level
Bailie, 2006	Blood pressure, Disease severity	Both, Patient level
Baker, 2001	Health care utilization	Systems level, Patient level
Baker, 2011	Cost, Overall mortality and cause-specific mortality	Systems level, Patient level
Bakitas, 2004	Compliance, Satisfaction, Level of care	Patient level, Systems level
Barrera, 2011	Compliance, Cognition, Self-efficacy, Attitude, Diet, Function, Mental health, BMI + Glycemic control, BMI	Patient level
Behnke, 2003	Health care utilization, Quality of Life, Function, Pulmonary function	Systems level, Patient level
Belardinelli, 2012	Compliance, Function, Pulmonary function, CV health, Quality of Life, Safety, Health care utilization, Overall mortality and cause-specific mortality	Patient level
Berg, 2007	Health care utilization, Glycemic control	Systems level, Patient level
Bocchi, 2008	Compliance, Health care utilization, Diet, Quality, Feasibility , Overall mortality and cause-specific mortality, Health care utilization	Patient level, Systems level
Chen, 2010	Health care utilization, Glycemic control, Quality of Life	Systems level
Cheng, 2012	Health care utilization, Cost	Patient level, Systems level
Chin, 2007	Glycemic control, Cholesterol, Renal function, Health care utilization, Blood pressure	Systems level, Patient level
Coleman, 2001	Health status, Function, Health care utilization, Compliance, Cost, Satisfaction	Patient level, Systems level
Corkery, 1997	Compliance, Behaviour, Glycemic control	Patient level
Daniel, 1999	Glycemic control, BMI, Blood pressure, Function, Knowledge, Function, Cholesterol, Weight	Patient level
Del Sindaco, 2007	Health care utilization, CV health, Health care utilization, Overall mortality and cause-specific mortality, Health status, Quality of Life, Cost	Systems level, Both, Patient level
Dennison, 2007	Overall mortality and cause-specific mortality, Blood pressure, CV health, Renal function, Cholesterol, Disease severity, BMI, Health	Systems level, Patient level

	care utilization, Compliance, Blood pressure, Attitude, Barrier/facilitator, Behaviour	
Desouza, 2010	Glycemic control, Cholesterol, Blood pressure	Patient level
Erfurt, 1990	Compliance, Blood pressure , Health care utilization	Patient level, Systems level
Fihn, 2004	Health care utilization, Function, Mental health, Satisfaction, Blood pressure, Alcohol	Systems level, Both, Patient level
Froehlich, 2002	Health care utilization, Cost, Overall mortality and cause-specific mortality, CV health , CV health + Overall mortality and cause-specific mortality	Systems level
Gaede, 2003	BMI, Blood pressure, Smoking, Diet, Alcohol, BMI, Function, Glycemic control, Cholesterol, Renal function, CV health, Blood pressure + cholesterol + Glycemic control , Vision health, Nerve function, Glycemic control, Vision	Patient level, Systems level
Gary, 2003	Glycemic control, Cholesterol, Blood pressure, Diet, Function, BMI, Motivation	Patient level
Getpreechaswas, 2007	Knowledge, Attitude, Diet, Function, Mental health, Smoking, Alcohol, BMI, Satisfaction, Cost	Patient level, Systems level
Giannuzzi, 2008	Overall mortality and cause-specific mortality, CV health, Health care utilization, CV health + Overall mortality and cause-specific mortality, Function, Mental health, Diet, Cholesterol, Glycemic control, BMI, Smoking	Systems level, Patient level
Gibson, 2011	Utilization, Cost, Compliance	Patient level
Grosbois, 1999	Pulmonary function, Function	Patient level
Hedges, 2000	CV health, Overall mortality and cause-specific mortality, Health care utilization, Blood pressure	Systems level, Patients level, Both
Hess, 2007	Blood pressure	Patient level
Higginbotham, 1999	CV health, Overall mortality and cause-specific mortality, Health care utilization, Smoking, BMI, Blood pressure, Cholesterol, Compliance	Systems level, Both
Hopper, 1984	Glycemic control, Health care utilization, BMI	Patient level, both
Hughes, 2010	Compliance, Diet, Pain, Function, BMI, Mental health, Self-efficacy	Patient level
Huizinga, 2010	Glycemic control, BMI, Health care utilization, Satisfaction, Attitude	Patient level, System level
Inglis, 2006	Overall mortality and cause-specific mortality, Health care utilization, Cost	Patient level, Both, Systems level
Jia, 2009	Comorbidity, Health care utilization, Overall mortality and cause-specific mortality	Patient level, Systems level

Jovanovic, 2004	Glycemic control, Health care utilization, BMI, Blood pressure, Cholesterol	Patient level, Systems level, Both
Kelso, 1996	Health care utilization, Knowledge, Pulmonary function, Quality of Life, Safety	Patient level
Kim, 2011	Blood pressure, Mental health, Self-efficacy, Knowledge, Compliance	Patient level
Krishan, 1979	Blood pressure	Systems level
Chavannes, 2009	Pulmonary function, Quality of Life	Patient level
Lawrence, 2008	Compliance, Health care utilization	Systems level
Montero, 2005	Overall mortality and cause-specific mortality, Safety	Systems level
Mildestvedt, 2008	Function, Attitude, Motivation	Patient level
Morisky, 1983	BMI, Compliance, Blood pressure, Overall mortality and cause-specific mortality	Patient level
Olson, 2009	Cholesterol, Overall mortality and cause-specific mortality, Health care utilization, Compliance, Blood pressure	Systems level, Patient level, Both
Perk, 1989	Compliance, Safety, Employment, Blood pressure, CV health, Mental health, Health care utilization, Function, Attitude, Behaviour, Liver function, Overall mortality and cause-specific mortality, Motivation	Both,Patient level
Phillips, 2005	Glycemic control, Blood pressure, Cholesterol, Behaviour, Blood pressure + Cholesterol + Glycemic control	Patient level, Systems level
Pill, 1998	Glycemic control, BMI, Blood pressure, Compliance, Safety, Satisfaction, Health status, Health care utilization	Patient level
Radziewicz, 2009	Fidelity, Compliance, Health care utilization, Satisfaction	Systems level, Both
Reichard, 1996	Glycemic control, Vision health, Renal function, Blood pressure, Health care utilization, Nerve function, Cognition, BMI, Health status, Overall mortality and cause-specific mortality, Disease severity, Vision	Patient level, Systems level, Both
Rothschild, 2012	Glycemic control, Blood pressure, BMI, Health care utilization, Behaviour, Compliance	Patient level, Systems level
Rowley, 2000	BMI, Glycemic control, Diet + Function, BMI + Glycemic control + Cholesterol	Patient level, Both
Skinner, 2000	Compliance, Knowledge, Satisfaction, Barrier/facilitator	Patient level
Stroebel, 2000	Blood pressure, Health care utilization	Patient level

Svetkey, 2009	Blood pressure, BMI, Diet, Function, Glycemic control, Cholesterol	Patient level
Tamone, 2012	Compliance, Discontinuation	Both, Systems level, Patient level
van Wetering, 2009	Quality of Life, Pulmonary function, Function, BMI, Attitude	Patient level
Weiss, 1984	Health status, Function, Mental health, Satisfaction, Overall mortality and cause-specific mortality, Health care utilization, Level of care, Cost, Health care utilization	Patient level, Systems level, Both
Wisse, 2010	Health status, Function, Blood pressure, BMI, Glycemic control, Renal function, Cholesterol	Patient level
Xian, 2010	Health care utilization	Systems level
